# Supplementary material for: Risk assessment models for venous thromboembolism in pregnancy and in the puerperium: a systematic review
Source: BMJ Open. 2022 Oct 12;12(10):e065892. doi: 10.1136/bmjopen-2022-065892 (PMC9562726; doi:10.1136/bmjopen-2022-065892)
Supplement: Supplementary data [file bmjopen-2022-065892supp001.pdf]

APPENDICES

Pandor et al. Risk assessment models for venous thromboembolism in pregnancy and in the puerperium – a systematic review

Contents

Appendix S1	Literature search strategies
Appendix S2	List of excluded studies with rationale
Appendix S3	Simplified summary of widely evaluated generic RAMs, their associated characteristics and composite clinical variables

APPENDIX S1 LITERATURE SEARCH STRATEGIES

**Database searched:**	**Ovid MEDLINE(R) Epub Ahead of Print, In-Process & Other Non-Indexed Citations, Ovid MEDLINE(R) Daily, Ovid MEDLINE and Versions(R)**
**Platform or provider used:**	**Ovid SP**
**Date of coverage:**	**1946 to February 2021**
**Search undertaken:**	**February 2021**

1 Pregnant Women/ or exp Pregnancy Complications/ or exp Maternal Health Services/ or exp Fetal Monitoring/ or exp Prenatal Diagnosis/ or Perinatal Care/ or Labor pain/ or Analgesia, Obstetric/ or exp Obstetric Surgical Procedures/ or exp Postpartum Period/
2 (pregnan* or antenatal* or ante-natal* or prenatal* or pre-natal* or gestational* or matern* or perinatal* or peri-natal* or postnatal* or post-natal* or postpartum or post-partum or puerper* or obstetric).mp.
3 1 or 2
4 pulmonary embolism/ or thromboembolism/ or venous thromboembolism/ or venous thrombosis/ or upper extremity deep vein thrombosis/
5 (((venous or vein) adj (thrombosis or thromboses or thrombus or thromboemboli*)) or (dvt or vte) or ((pulmonary or lung) adj3 (embolism or emboli or embolus or emboliz* or thromboemboli*))).ti,ab.
6 4 or 5
7 editorial/ or news/ or exp historical article/ or anecdotes as topic/ or comment/ or case report/ or (letter or comment).ti.
8 randomized controlled trial/ or random*.ti,ab.
9 7 not 8
10 animals/ not humans/
11 exp animals, laboratory/
12 exp animal experimentation/
13 exp models, animal/
14 exp rodentia/
15 (rat or rats or mouse or mice).ti.
16 9 or 10 or 11 or 12 or 13 or 14 or 15
17 6 not 16
18 (risk* adj2 assess*).ti,ab.
19 ((score* or scoring) adj2 (tool* or system*)).ti,ab.
20 ((risk* or predict* or prognos*) adj4 (tool* or rule* or index* or indices or score* or scoring or scale* or model* or system* or algorithm* or stratif* or criteria or calculat*)).ti,ab.
21 department of health.ti,ab,au.
22 (guidance or guideline*).ti,hw,pt.
23 18 or 19 or 20 or 21 or 22
24 17 and 23
25 3 and 24

**Databases searched:**	**EMBASE**
**Platform or provider used:**	**Ovid SP**
**Date of coverage:**	**1974 to February 2021**
**Search undertaken:**	**February 2021**

1 exp pregnancy/ or maternal health service/ or exp pregnancy complication/ or exp fetus monitoring/ or exp prenatal diagnosis/ or exp perinatal care/ or exp obstetric analgesia/ or exp labor pain/ or exp obstetrics/ or obstetric analgesia/ or exp obstetric operation/ or puerperium/
2 (pregnan* or antenatal* or ante-natal* or prenatal* or pre-natal* or gestational* or matern* or perinatal* or peri-natal* or postnatal* or post-natal* or postpartum or post-partum or puerper* or obstetric or labo?r).mp.
3 1 or 2
4 lung embolism/ or exp venous thromboembolism/ or exp vein thrombosis/ or upper extremity deep vein thrombosis/
5 (((venous or vein) adj (thrombosis or thromboses or thrombus or thromboemboli*)) or (dvt or vte) or ((pulmonary or lung) adj3 (embolism or emboli or embolus or emboliz* or thromboemboli*))).ti,ab.
6 4 or 5
7 editorial/ or comment/ or case report/ or (letter or comment).ti.
8 randomized controlled trial/ or random*.ti,ab.
9 7 not 8
10 exp animal/ not exp human/
11 (rat or rats or mouse or mice).ti.
12 9 or 10
13 6 not 12
14 (risk* adj2 assess*).ti,ab.
15 ((score* or scoring) adj2 (tool* or system*)).ti,ab.
16 ((risk* or predict* or prognos*) adj4 (tool* or rule* or index* or indices or score* or scoring or scale* or model* or system* or algorithm* or stratif* or criteria or calculat*)).ti,ab.
17 department of health.ti,ab,au.
18 (guidance or guideline*).ti,hw,pt.
19 14 or 15 or 16 or 17 or 18
20 13 and 19
21 3 and 20

**Databases searched:**	**Cochrane CENTRAL Register of Randomised Controlled Trials & Cochrane Database of Systematic Reviews**
**Platform or provider used:**	**www.thecochranelibrary.com**
**Date of coverage:**	**Inception to February 2021**
**Search undertaken:**	**February 2021**

#1 MeSH descriptor: [Pregnancy] explode all trees
#2 MeSH descriptor: [Pregnancy Complications] 1 tree(s) exploded
#3 MeSH descriptor: [Maternal Health Services] explode all trees
#4 MeSH descriptor: [Fetal Monitoring] explode all trees
#5 MeSH descriptor: [Perinatal Care] explode all trees
#6 MeSH descriptor: [Labor Pain] explode all trees
#7 MeSH descriptor: [Analgesia, Obstetrical] explode all trees
#8 MeSH descriptor: [Obstetric Surgical Procedures] explode all trees
#9 MeSH descriptor: [Postpartum Period] explode all trees
#10 (pregnan* or antenatal* or "ante-natal*" or prenatal* or "pre-natal*" or gestational* or matern* or perinatal* or "peri-natal*" or postnatal* or "post-natal*" or postpartum or "post-partum" or puerper* or obstetric):ti,ab,kw (Word variations have been searched)
#11 MeSH descriptor: [Pulmonary Embolism] explode all trees
#12 MeSH descriptor: [Venous Thromboembolism] explode all trees
#13 MeSH descriptor: [Venous Thrombosis] explode all trees
#14 MeSH descriptor: [Upper Extremity Deep Vein Thrombosis] explode all trees
#15 ((venous or vein) near/2 (thrombosis or thromboses or thrombus or thromboemboli*)):ti,ab,kw OR ((dvt or vte)):ti,ab,kw OR ((pulmonary or lung) near/2 (embolism or emboli or embolus or emboliz* or thromboemboli*)):ti,ab,kw (Word variations have been searched)
#16 #1 or #2 or #3 or #4 or #5 or #6 or #7 or #8 or #9 or #10
#17 #11 or #12 or #13 or #14 or #15
#18 #16 and #17
#19 (risk* or predict* or prognos*):ti,ab,kw AND (tool* or rule* or index* or indices or score* or scoring or scale* or model* or system* or algorithm* or stratif* or criteria or calculat*):ti,ab,kw OR ((pulmonary or lung) near/3 (embolism or emboli or embolus or emboliz* or thromboemboli*)):ti,ab,kw (Word variations have been searched)
#20 (score* or scoring) near/2 (tool* or system*)
#21 guidance or guideline* or "department of health"
#22 #19 or #20 or #21
#23 #18 and #22

APPENDIX S2 LIST OF EXCLUDED STUDIES WITH RATIONALE

	Authors, year	Reason for exclusion
1.	Abdul Sultan et al., 2013 [1]	Not a RAM for predicting the risk of developing VTE in pregnancy or the puerperium
2.	Ahmadzia et al., 2019 [2]	Abstract of included full text study (Ellis-Kahana 2020)
3.	Alsayegh et al., 2016 [3]	No relevant/useable outcome data
4.	Bahl et al., 2010 [4]	Not a RAM for predicting the risk of developing VTE in pregnancy or the puerperium
5.	Banfield et al., 2013 [5]	No relevant/useable outcome data
6.	Bare et al., 2013 [6]	Not a RAM for predicting the risk of developing VTE in pregnancy or the puerperium
7.	Barros et al., 2017 [7]	No relevant/useable outcome data
8.	Barros et al., 2017 [8]	No relevant/useable outcome data
9.	Barros et al., 2020 [9]	No relevant/useable outcome data
10.	Barros et al., 2011 [10]	No relevant/useable outcome data
11.	Bastek et al., 2011 [11]	Not a RAM for predicting the risk of developing VTE in pregnancy or the puerperium
12.	Beckett et al., 2013 [12]	No relevant/useable outcome data
13.	Berkin et al., 2016 [13]	No relevant/useable outcome data
14.	Blondon et al., 2015 [14]	Not a RAM for predicting the risk of developing VTE in pregnancy or the puerperium
15.	Blondon and Hugon-Rodin 2017 [15]	Commentary
16.	Campbell 2013 [16]	No relevant/useable outcome data
17.	Cavazza et al., 2010 [17]	Abstract of included full text study (Cavazza 2012)
18.	Chauleur et al., 2017 [18]	Data overlap - patients included in Chauleur 2018 (included study)
19.	Chauleur et al., 2010 [19]	No relevant/useable outcome data
20.	Chauleur et al., 2018 [20]	No relevant/useable outcome data
21.	Cooley et al., 2016 [21]	No relevant/useable outcome data
22.	Creagh et al., 2014 [22]	Not a RAM for predicting the risk of developing VTE in pregnancy or the puerperium
23.	Creagh et al., 2013 [23]	Not a RAM for predicting the risk of developing VTE in pregnancy or the puerperium
24.	Crowley et al., 2013 [24]	Abstract of excluded full text study (Crowley 2017)
25.	Crowley et al., 2017 [25]	No relevant/useable outcome data
26.	Crowley et al., 2013 [26]	Abstract of excluded full text study (Crowley 2017)
27.	Cunningham et al., 2015 [27]	Not a RAM for predicting the risk of developing VTE in pregnancy or the puerperium

28.	Cutts et al., 2014 [28]	Not a RAM for predicting the risk of developing VTE in pregnancy or the puerperium
29.	Cutts et al., 2011 [29]	Not a RAM for predicting the risk of developing VTE in pregnancy or the puerperium
30.	Dargaud et al., 2015 [30]	Abstract of included full text study (Dargaud 2017)
31.	Dargaud et al., 2009 [31]	Data overlap - patients included in Dargaud 2017 (included study)
32.	Dargaud et al., 2009 [32]	No relevant/useable outcome data
33.	Davis and Hadpawat-Lee 2017 [33]	No relevant/useable outcome data
34.	Dentali et al., 2020 [34]	Not a RAM for predicting the risk of developing VTE in pregnancy or the puerperium
35.	Francis Kim et al., 2020 [35]	No relevant/useable outcome data
36.	Francis Kim et al., 2020 [36]	No relevant/useable outcome data
37.	Fuller et al., 2018 [37]	Not a RAM for predicting the risk of developing VTE in pregnancy or the puerperium
38.	Gassmann et al., 2020 [38]	Abstract of included full text study (Gassmann 2020)
39.	Gerhardt et al., 2016 [39]	Not a RAM for predicting the risk of developing VTE in pregnancy or the puerperium
40.	Gherghe et al., 2012 [40]	No relevant/useable outcome data
41.	Goffman et al., 2009 [41]	No relevant/useable outcome data
42.	Gomez et al., 2020 [42]	No relevant/useable outcome data
43.	Grille et al., 2015 [43]	No relevant/useable outcome data
44.	Goodfellow et al., 2017 [44]	No relevant/useable outcome data
45.	Grant et al., 2016 [45]	No relevant/useable outcome data
46.	Handa et al., 2015 [46]	No relevant/useable outcome data
47.	Harris et al., 2016 [47]	No relevant/useable outcome data
48.	Hayes-Ryan and Byrne 2011 [48]	No relevant/useable outcome data
49.	Hayes-Ryan and Byrne 2012 [49]	No relevant/useable outcome data
50.	Heath and Goodfellow 2016 [50]	No relevant/useable outcome data
51.	Henke and Pannucci 2010 [51]	Review
52.	Kazi et al., 2020 [52]	Not a RAM for predicting the risk of developing VTE in pregnancy or the puerperium
53.	Lacoss and Jheeta 2017 [53]	Not a RAM for predicting the risk of developing VTE in pregnancy or the puerperium
54.	Li et al., 2018 [54]	Not a RAM for predicting the risk of developing VTE in pregnancy or the puerperium
55.	Lindqvist 2018 [55]	Letter
56.	Lindqvist and Hellgren 2011 [56]	No relevant/useable outcome data
57.	Lindqvist et al., 2002 [57]	No relevant/useable outcome data

58.	Lou Mercade et al., 2017 [58]	No relevant/useable outcome data
59.	Marks and Maiti 2018 [59]	No relevant/useable outcome data
60.	Mcarthur et al., 2011 [60]	No relevant/useable outcome data
61.	Mpouzouki et al., 2013 [61]	No relevant useable outcome data
62.	Naidoo et al., 2019 [62]	No relevant/useable outcome data
63.	Nct 2018 [63]	Protocol
64.	Noone et al., 2013 [64]	No relevant/useable outcome data
65.	O'Connor et al., 2011 [65]	Not a RAM for predicting the risk of developing VTE in pregnancy or the puerperium
66.	O'Keefe et al., 2019 [66]	Not a RAM for predicting the risk of developing VTE in pregnancy or the puerperium
67.	Omunakwe et al., 2017 [67]	No relevant/useable outcome data
68.	Orfanelli et al., 2017 [68]	No relevant/useable outcome data
69.	O'Shaughnessy et al., 2017 [69]	No relevant/useable outcome data
70.	O'Shaughnessy et al., 2018 [70]	No relevant/useable outcome data
71.	O'Shaughnessy et al., 2019 [71]	No relevant/useable outcome data
72.	O'Sullivan et al., 2020 [72]	No relevant/useable outcome data
73.	O'Sullivan et al., 2009 [73]	Not a RAM for predicting the risk of developing VTE in pregnancy or the puerperium
74.	Ottawa Hospital Research Institute and Leo Pharma 2002 [74]	Protocol
75.	Palmerola et al., 2016 [75]	No relevant/useable outcome data
76.	Pannucci and Fleming 2017 [76]	Not a RAM for predicting the risk of developing VTE in pregnancy or the puerperium
77.	Pierce-Williams et al., 2018 [77]	No relevant/useable outcome data
78.	Potdar et al., 2006 [78]	No relevant/useable outcome data
79.	Rahim et al., 2020 [79]	Not a RAM for predicting the risk of developing VTE in pregnancy or the puerperium
80.	Righini et al., 2013 [80]	Not a RAM for predicting the risk of developing VTE in pregnancy or the puerperium
81.	Righini and Le Gal 2013 [81]	Not a RAM for predicting the risk of developing VTE in pregnancy or the puerperium
82.	Ryan 2019 [82]	No relevant/useable outcome data
83.	Saad et al., 2018 [83]	Abstract of included full text study (Tran 2019)
84.	Santos et al., 2015 [84]	No relevant/useable outcome data
85.	Schoenbeck et al., 2011 [85]	No relevant/useable outcome data
86.	Sellappan et al., 2012 [86]	Not a RAM for predicting the risk of developing VTE in pregnancy or the puerperium
87.	Shacaluga et al., 2017 [87]	No relevant/useable outcome data

88.	Tan and Wisdom 2006 [88]	No relevant/useable outcome data
89.	Tang and Marsden 2011 [89]	No relevant/useable outcome data
90.	Taylor et al., 2000 [90]	No relevant/useable outcome data
91.	Testa et al., 2010 [91]	No relevant/useable outcome data
92.	Testa et al., 2013 [92]	No relevant/useable outcome data
93.	Touhami et al., 2018 [93]	Not a RAM for predicting the risk of developing VTE in pregnancy or the puerperium
94.	Usoro et al., 2019 [94]	Not a RAM for predicting the risk of developing VTE in pregnancy or the puerperium
95.	Valdre et al., 2016 [95]	No relevant/useable outcome data
96.	Von Hawrylak 2018 [96]	No relevant/useable outcome data
97.	Zhang et al., 2020 [97]	No relevant/useable outcome data

REFERENCES (APPENDIX S2)

1. Abdul Sultan A, West J, Tata LJ, et al. Risk of first venous thromboembolism in pregnant women in hospital: population based cohort study from England. *BMJ (Clinical Research Ed)* 2013;347:f6099. doi: https://dx.doi.org/10.1136/bmj.f6099
2. Ahmadzia HK, Ellis-Kahana JK, Sparks AD, et al. 859: Predicting venous thromboembolism in obese pregnant women in a national study. *American Journal of Obstetrics and Gynecology* 2019;220(1 Supplement):S559-S60. doi: http://dx.doi.org/10.1016/j.ajog.2018.11.882
3. Alsayegh F, Al-Jassar W, Wani S, et al. Venous Thromboembolism Risk and Adequacy of Prophylaxis in High Risk Pregnancy in the Arabian Gulf. *Current Vascular Pharmacology* 2016;14(4):368-73.
4. Bahl V, Hu HM, Henke PK, et al. A validation study of a retrospective venous thromboembolism risk scoring method. *Annals of Surgery* 2010;251(2):344-50. doi: https://dx.doi.org/10.1097/SLA.0b013e3181b7fca6
5. Banfield DA, Page LM, Cotzias CS, et al. Postnatal risk assessment of venous thromboembolism (VTE). *Archives of Disease in Childhood: Fetal and Neonatal Edition* 2013;98(SUPPL. 1) doi: http://dx.doi.org/10.1136/archdischild-2013-303966.121
6. Bare LA, De Haan HG, Arellano AR, et al. A simple genetic thrombosis score of five single nucleotide polymorphisms is associated with risk of first venous thrombosis in pregnant women. *Blood* 2013;122(21)
7. Barros V, Hase E, Igai AM, et al. Cancer and venous thromboembolism in pregnancy. *Research and Practice in Thrombosis and Haemostasis* 2017;1(Supplement 1):54. doi: http://dx.doi.org/10.1002/rth2.12012
8. Barros V, Igai AM, Bortolotto MR, et al. Obesity and thromboembolic risk during hospitalization in pregnancy: Preliminary results. *Research and Practice in Thrombosis and Haemostasis* 2017;1(Supplement 1):1030.
9. Barros V, Igai A, Fernanda B, et al. Preventing maternal death and morbidity from venous thromboembolism (VTE): Results from a VTE risk score trial during hospitalization. *Research and Practice in Thrombosis and Haemostasis* 2020;4(SUPPL 1):1197-98. doi: http://dx.doi.org/10.1002/rth2.12393
10. Barros V, Oliveira A, Spadotto F, et al. Thrombosis risk score in pregnancy: Preliminary results from a model. *Journal of Perinatal Medicine* 2011;39(SUPPL. 1) doi: http://dx.doi.org/10.1515/jpm-2012-1008
11. Bastek JA, Dainty E, Srinivas SK, et al. Pulmonary embolism in pregnancy: Do the diagnostic algorithms from non-pregnant populations apply? *American Journal of Obstetrics and Gynecology* 2011;204(1 SUPPL.):S317. doi: http://dx.doi.org/10.1016/j.ajog.2010.10.834
12. Beckett V, Graham I, Keriakos R. Audit of prescribing and administration of thromboprophylaxis medication and stockings to in-patients of the obstetric and gynaecological wards at the

Jessops Wing, Sheffield teaching hospitals. *BJOG: An International Journal of Obstetrics and Gynaecology* 2013;120(SUPPL. 1):437-38. doi: http://dx.doi.org/10.1111/1471-0528.12301

13. Berkin JA, Lee C, Landsberger E, et al. Scorecard implementation improves identification of postpartum patients at risk for venous thromboembolism. *Journal of Healthcare Risk Management: The Journal of the American Society for Healthcare Risk Management* 2016;36(1):8-13. doi: https://dx.doi.org/10.1002/jhrm.21229
14. Blondon M, Harrington L, Boehlen F, et al. Pre-pregnancy BMI and delivery BMI as risk factors for postpartum VTE: A population-based, case-control study. *Journal of Thrombosis and Haemostasis* 2015;13(SUPPL. 2):106. doi: http://dx.doi.org/10.1111/jth.12993
15. Blondon M, Hugon-Rodin J. A clinical risk score to predict the incidence of postpartum venous thromboembolism. *Evidence-Based Medicine* 2017;22(3):98. doi: http://dx.doi.org/10.1136/ebmed-2017-110680
16. Campbell MJ. Full audit cycle assessing how current antenatal inpatients are risk assessed for venous thromboembolic (VTE) disease as an inpatient and antenatally at Ninewells Hospital, Dundee, Scotland, October 2012. *BJOG: An International Journal of Obstetrics and Gynaecology* 2013;120(SUPPL. 1):472. doi: http://dx.doi.org/10.1111/1471-0528.12301
17. Cavazza S, Rainaldi MP, Bonazzi F, et al. Thomboprophylaxis following cesarean section: A one site prospective pilot study to evaluate the application of a risk score model. *Pathophysiology of Haemostasis and Thrombosis* 2010;37(SUPPL. 1):A4. doi: http://dx.doi.org/10.1159/000318095
18. Chauleur C, Gris JC, Laporte S, et al. Implementation of risk score-guided prophylaxis in over 2000 pregnant women at risk of thrombotic events: Impact on morbidity. A quasi-experimental prospective study. *Thrombosis Research* 2017;151(Supplement 1):S107-S08.
19. Chauleur C, Gris JC, Laporte S, et al. Use of the Delphi method to facilitate antithrombotics prescription during pregnancy. *Thrombosis Research* 2010;126(2):88-92. doi: https://dx.doi.org/10.1016/j.thromres.2010.01.012
20. Chauleur C, Gris J-C, Laporte S, et al. Benefit of Risk Score-Guided Prophylaxis in Pregnant Women at Risk of Thrombotic Events: A Controlled Before-and-After Implementation Study. *Thrombosis and Haemostasis* 2018;118(9):1564-71. doi: https://dx.doi.org/10.1055/s-0038-1668524
21. Cooley SM, Donnelly JC, Deering M, et al. Thrombocalc: Personalized postpartum VTE risk assessment in a high-throughput environment. *American Journal of Obstetrics and Gynecology* 2016;214(1 SUPPL. 1):S26-S27.
22. Creagh MD, Davies B, Webborn A, et al. Practice using a programme for avoidance of venous thrombo-embolism (VTE) in pregnancy; Is post delivery risk assessment undertaken and

effective? *British Journal of Haematology* 2014;165(SUPPL. 1):44-45. doi: http://dx.doi.org/10.1111/bjh.12802

23. Creagh MD, Dehnel A, Rider L, et al. Does systematic risk assessment in pregnancy identify women at risk for venous thromboembolism and so avoid thrombosis? Experience of an 18 month programme based on national guidance. *Journal of Thrombosis and Haemostasis* 2013;11(SUPPL. 2):867.
24. Crowley MP, Noone C, Higgins JR, et al. Venous thromboembolism (VTE) prophylaxis in hospitalized obstetric patients in Ireland: A multicentre cross-sectional study. *Journal of Thrombosis and Haemostasis* 2013;11(SUPPL. 2):739.
25. Crowley MP, Noone C, Higgins JR, et al. A Multicentre Study of Thromboprophylaxis in Pregnancy. *Irish Medical Journal* 2017;110(5):567.
26. Crowley MP, Noone C, Higgins JR, et al. Venous thromboembolism (VTE) prophylaxis in hospitalized obstetric patients: A multicentre cross-sectional study. *Haematologica* 2013;98(SUPPL. 1):438.
27. Cunningham Y, MacDonald H, Coutts S. Bettering antenatal and postnatal risk assessment for venous thromboembolism - A quality improvement approach. *BJOG: An International Journal of Obstetrics and Gynaecology* 2015;122(SUPPL. 2):263. doi: http://dx.doi.org/10.1111/14710528.13383
28. Cutts BA, Tran HA, Merriman E, et al. The utility of the Wells clinical prediction model and ventilation-perfusion scanning for pulmonary embolism diagnosis in pregnancy. *Blood Coagulation & Fibrinolysis: An International Journal in Haemostasis and Thrombosis* 2014;25(4):375-8. doi: https://dx.doi.org/10.1097/MBC.0000000000000054
29. Cutts BA, Tran H, Merriman E, et al. The utility of the wells clinical prediction model and ventilation-perfusion scanning for pulmonary embolism in pregnancy. *Journal of Thrombosis and Haemostasis* 2011;9(SUPPL. 2):631. doi: http://dx.doi.org/10.1111/j.1538-7836.2011.04380_3.x
30. Dargaud YG, Rugeri L, Fleury C, et al. Individually tailored prophylaxis using a risk score for the management of pregnant women with increased risk of venous thromboembolism. *Blood* 2015;126(23):889.
31. Dargaud Y, Rugeri L, Vergnes MC, et al. A risk score for the management of pregnant women with increased risk of venous thromboembolism: a multicentre prospective study. *British Journal of Haematology* 2009;145(6):825-35. doi: https://dx.doi.org/10.1111/j.1365-2141.2009.07698.x
32. Dargaud Y, Rugeri L, Vergnes MC, et al. Management of pregnancies with high risk of thrombosis: A multicenter prospective study. *Journal of Thrombosis and Haemostasis* 2009;7(S2):1060-61. doi: http://dx.doi.org/10.1111/j.1538-7836.2009.03473-2.x

33. Davis B, Hadpawat-Lee A. Venous thromboembolism: Comparing risk assessment tools for postpartum prophylaxis in a teaching hospital. *Obstetrics and Gynecology* 2017;129(Supplement 1):114S.
34. Dentali F, Fontanella A, Cohen AT, et al. Derivation and Validation of a Prediction Model for Venous Thromboembolism in Primary Care. *Thrombosis and Haemostasis* 2020;120(4):692-701. doi: http://dx.doi.org/10.1055/s-0040-1701483
35. Francis Kim AP, Saleh M, Melendez Torres A, et al. Age and body mass index can screen for VTE risk at labor and delivery admission. *Obstetrics and Gynecology* 2020;135(Supplement 1):39S.
36. Francis Kim AP, Saleh M, Torres AM, et al. Impact of delivery-related factors on venous thromboembolism risk during labor. *Obstetrics and Gynecology* 2020;135(Supplement 1):128S.
37. Fuller GW, Nelson-Piercy C, Hunt BJ, et al. Consensus-derived clinical decision rules to guide advanced imaging decisions for pulmonary embolism in pregnancy and the postpartum period. *European Journal of Emergency Medicine: Official Journal of the European Society for Emergency Medicine* 2018;25(3):221-22. doi: https://dx.doi.org/10.1097/MEJ.0000000000000477
38. Gassmann N, Viviano M, Fontana P, et al. Comparison of recommended postpartum thromboprophylaxis and of absolute risk thresholds according to the RCOG, ACCP and ACOG guidelines. *Research and Practice in Thrombosis and Haemostasis* 2020;4(SUPPL 1):32-33. doi: http://dx.doi.org/10.1002/rth2.12401
39. Gerhardt A, Scharf RE, Greer IA, et al. Hereditary risk factors for thrombophilia and probability of venous thromboembolism during pregnancy and the puerperium. *Blood* 2016;128(19):2343-49. doi: http://dx.doi.org/10.1182/blood-2016-03-703728
40. Gherghe M, Moss H, Gibson J, et al. Risk assessment to reduce thromboembolic disease in obstetric practice: A regional audit. *Archives of Disease in Childhood: Fetal and Neonatal Edition* 2012;97(SUPPL. 1):A49. doi: http://dx.doi.org/10.1136/fetalneonatal-2012-301809.156
41. Goffman D, Fisher N, Kowenski J, et al. Utilization of a checklist to evaluate risk for postpartum venous thromboembolism. *American Journal of Obstetrics and Gynecology* 2009;201(6 SUPPL. 1):S297. doi: http://dx.doi.org/10.1016/j.ajog.2009.10.848
42. Gomez D, Orfanelli T, Awomolo A, et al. A comparison of pregnancy-specific risk scoring systems for venous thromboembolic pharmacoprophylaxis in hospitalized maternity patients. *The Journal of Maternal-Fetal & Neonatal Medicine: The Official Journal of the European Association of Perinatal Medicine, the Federation of Asia and Oceania Perinatal Societies, the International Society of Perinatal Obstetricians* 2020:1-8. doi: https://dx.doi.org/10.1080/14767058.2020.1832072

43. Grille S, Castro V, Turcatti P, et al. Prophylaxis for venous thromboembolic disease in pregnancy and postpartum period. *Haematologica* 2015;100(SUPPL. 1):626.
44. Goodfellow A, Heath S, George L, et al. Perinatal venous thromboembolism state based prevention strategy: Midwifery risk assessment tool. *Thrombosis Research* 2017;151(Supplement 1):S132.
45. Grant GH, Merriman JB, Hoffman MK. Implementation and efficacy of a formalized venous thromboembolism prevention strategy in the peripartum population. *American Journal of Obstetrics and Gynecology* 2016;214(1 SUPPL. 1):S227-S28.
46. Handa S, Singh S, Bhandal N. Retrospective audit of venous thromboembolism risk assessment in obstetric postoperative patients. *Anaesthesia* 2015;70(SUPPL. 3):72. doi: http://dx.doi.org/10.1111/anae.13136
47. Harris C, Sulmers C, Groesch K, et al. Venous thromboembolism: Padua prediction score in the obstetric patient. *Obstetrics and Gynecology* 2016;127(Supplement 1):88S. doi: http://dx.doi.org/10.1097/01.AOG.0000483795.00678.28
48. Hayes-Ryan D, Byrne B. Thromboprophylaxis in pregnancy the practical implications of guidelines. *American Journal of Obstetrics and Gynecology* 2011;204(1 SUPPL.):S315-S16. doi: http://dx.doi.org/10.1016/j.ajog.2010.10.829
49. Hayes-Ryan D, Byrne BM. Prevention of thrombosis in pregnancy: how practical are consensus derived clinical practice guidelines? *Journal of Obstetrics and Gynaecology: The Journal of the Institute of Obstetrics and Gynaecology* 2012;32(8):740-2. doi: https://dx.doi.org/10.3109/01443615.2012.693982
50. Heath S, Goodfellow A. Maternal venous thromboembolism (VTE) risk assessment. *Journal of Paediatrics and Child Health* 2016;52(Supplement 2):69. doi: http://dx.doi.org/10.1111/jpc.13194
51. Henke PK, Pannucci CJ. Venous thromboembolism risk factor assessment and prophylaxis. *Phlebology* 2010;25(5):219-23. doi: http://dx.doi.org/10.1258/phleb.2010.010018
52. Kazi S, McLeod A, Berndl A. Approach to venous thromboembolism risk in women with physical disability in pregnancy-multidisciplinary survey. *Research and Practice in Thrombosis and Haemostasis* 2020;4(SUPPL 1):1283-84. doi: http://dx.doi.org/10.1002/rth2.12393
53. LaCoss E, Jheeta S. How accurate is information documented on inpatient electronic venous thromboembolism risk-assessment forms? *Journal of Pharmacy and Pharmacology* 2017;69(Supplement 1):13-14. doi: http://dx.doi.org/10.1111/jphp.12850
54. Li C, Zuo Y, Karp D, et al. Identifying clinical and epidemiological risk factors associated with thrombosis and pregnancy morbidity in a large cohort of Chinese APS patients. *International Journal of Rheumatic Diseases* 2018;21(Supplement 1):126-27. doi: http://dx.doi.org/10.1111/1756-185X.13361

55. Lindqvist PG. Re: Postpartum venous thromboembolism prophylaxis may cause more harm than benefit: a critical analysis of international guidelines through an evidence-based lens: Postpartum thromboprophylaxis is cost-effective using the Swedish thromboprophylaxis algorithm. *BJOG: An International Journal of Obstetrics and Gynaecology* 2018;125(9):1194-95. doi: http://dx.doi.org/10.1111/1471-0528.15266
56. Lindqvist PG, Hellgren M. Obstetric thromboprophylaxis: the Swedish guidelines. *Advances in hematology* 2011;2011:157483. doi: https://dx.doi.org/10.1155/2011/157483
57. Lindqvist PG, Kublikas M, Dahlback B. Individual risk assessment of thrombosis in pregnancy. *Acta Obstetricia et Gynecologica Scandinavica* 2002;81(5):412-6.
58. Lou Mercade AC, Saviron Cornudella R, Cornudella Lacasa R. AnticoagObs: An application for antenatal and postpartum risk assessment and prophylaxis of venous thromboembolism. *Thrombosis Research* 2017;151(Supplement 1):S127.
59. Marks D, Maiti S. Venous thromboembolism risk assessment and prescribing on the postnatal ward at north manchester general hospital: A clinical audit. *BJOG: An International Journal of Obstetrics and Gynaecology* 2018;125(Supplement 3):25. doi: http://dx.doi.org/10.1111/1471-0528.15493
60. McArthur L, Whittaker B, Rajasri A, et al. Venous thrombo-embolic risk in pregnancy and the puerperium: Can self-assessment identify risk and guide further care? *British Journal of Haematology* 2011;153(SUPPL. 1):84-85. doi: http://dx.doi.org/10.1111/j.13652141.2011.08609
61. Mpouzouki A, Bowles L, Beski S, et al. A retrospective review of risk factors for thrombosis in women presenting with venous thromboembolism (VTE) during pregnancy and the puerperium. *Thrombosis Research* 2013;131(SUPPL. 1):S82. doi: http://dx.doi.org/10.1016/S0049-3848%2813%2970070-7
62. Naidoo P, Mothilal R, Snyman LC. Assessment and management of venous thrombo-embolism risk during pregnancy and the puerperium (SAVE): The South African cohort. *South African Medical Journal* 2019;109(3):186-92. doi: https://dx.doi.org/10.7196/SAMJ.2019.v109i3.13487
63. NCT. Pregnancy and Risk of Venous Thromboembolism. *https://clinicaltrialsgov/show/NCT03659708* 2018
64. Noone CM, O'Shea S, Crowley MP, et al. Venous thromboembolism (VTE) risk assessment & prophylaxis in irish hospitalized obstetric patients: A nationwide cross-sectional study. *Blood* 2013;122(21)
65. O'Connor C, Moriarty J, Walsh J, et al. The application of a clinical risk stratification score may reduce unnecessary investigations for pulmonary embolism in pregnancy. *The Journal of Maternal-Fetal & Neonatal Medicine: The Official Journal of the European Association of Perinatal Medicine, the Federation of Asia and Oceania Perinatal Societies, the International*

Society of Perinatal Obstetricians 2011;24(12):1461-4. doi: https://dx.doi.org/10.3109/14767058.2011.614652

66. O'Keefe D, Hui L, Ho P, et al. Evaluation of global coagulation assays for assessment of clotting function and risk of venous thromboembolism in pregnancy and post-partum. *Australian and New Zealand Journal of Obstetrics and Gynaecology* 2019;59(Supplement 1):68-69. doi: http://dx.doi.org/10.1111/ajo.13067
67. Omunakwe HE, Roberts LN, Patel JP, et al. Re: A comparison of the recommendations for pharmacologic thromboembolism prophylaxis after caesarean delivery from the major guidelines: Impact on thromboprophylaxis rates of implementing Royal College of Obstetricians and Gynaecologists' guidance for reducing the risk of ante- and postnatal venous thromboembolism. *BJOG: An International Journal of Obstetrics and Gynaecology* 2017;124(5):831-32. doi: http://dx.doi.org/10.1111/1471-0528.14001
68. Orfanelli T, Awomolo A, Gomez D, et al. A comparison of peripartum scoring systems of anticoagulation for venous thromboembolic prophylaxis. *Obstetrics and Gynecology* 2017;129(Supplement 1):112S.
69. O'Shaughnessy F, Donnelly JC, Cooley SM, et al. Thrombocalc: implementation and uptake of personalized postpartum venous thromboembolism risk assessment in a high-throughput obstetric environment. *Acta obstetricia et gynecologica Scandinavica* 2017;96(11):1382-90. doi: https://dx.doi.org/10.1111/aogs.13206
70. O'Shaughnessy F, Donnelly J, Cooley S, et al. Initiating postpartum pharmacological thromboprophylaxis: A comparison of international recommendations. *American Journal of Obstetrics and Gynecology* 2018;218(1 Supplement 1):S493-S94.
71. O'Shaughnessy F, Ni Ainle F, Jennifer D, et al. Preventing postpartum venous thromboembolism; impact of systematic VTE risk assessment. *Research and Practice in Thrombosis and Haemostasis* 2019;3(Supplement 1):225-26. doi: http://dx.doi.org/10.1002/rth2.12227
72. O'Sullivan C, Christensen K, Gallagher G, et al. Peripartum vte risk assessment in a tertiary centre: An audit. *Journal of Paediatrics and Child Health* 2020;56(SUPPL 1):143. doi: http://dx.doi.org/10.1111/jpc.14868
73. O'Sullivan C, Moriarty J, Walsh J, et al. Application of a clinical risk stratification score in pregnancy and the puerperium - Can unnecessary investigations for pulmonary embolism be avoided? *American Journal of Obstetrics and Gynecology* 2009;201(6 SUPPL. 1):S68. doi: http://dx.doi.org/10.1016/j.ajog.2009.10.161
74. Ottawa Hospital Research Institute, LEO Pharma. The STOP CLOT Pilot Study: Study of Low Molecular Weight Heparin in High Risk Cesarean Section: https://ClinicalTrials.gov/show/NCT00225108, 2002.
75. Palmerola KL, D'Alton ME, Brock CO, et al. A comparison of recommendations for pharmacologic thromboembolism prophylaxis after caesarean delivery from three major

guidelines. *BJOG: An International Journal of Obstetrics and Gynaecology* 2016;123(13):2157-62. doi: https://dx.doi.org/10.1111/1471-0528.13706

76. Pannucci C, Fleming K. The electronic medical record underestimates venous thromboembolism risk using the 2005 caprini score compared with face-to-face interaction. *Journal of Vascular Surgery: Venous and Lymphatic Disorders* 2017;5(1):152-53. doi: http://dx.doi.org/10.1016/j.jvsv.2016.10.028
77. Pierce-Williams R, Cohen I, D'Adamo C, et al. Venous thromboembolism prophylaxis after cesarean section: A quality improvement project. *Obstetrics and Gynecology* 2018;131(Supplement 1):104S-05S.
78. Potdar N, Jabbar B, Burrell SJ. Thromboprophylaxis after vaginal delivery: a district general hospital experience. *Journal of Obstetrics and Gynaecology: The Journal of the Institute of Obstetrics and Gynaecology* 2006;26(1):24-6.
79. Rahim MN, Williamson C, Kametas NA, et al. Using pregnancy to assess risk and predict women's health. *EClinicalMedicine* 2020;20:100292. doi: http://dx.doi.org/10.1016/j.eclinm.2020.100292
80. Righini M, Jobic C, Boehlen F, et al. Predicting deep venous thrombosis in pregnancy: external validation of the LEFT clinical prediction rule. *Haematologica* 2013;98(4):545-8. doi: https://dx.doi.org/10.3324/haematol.2012.072009
81. Righini M, Le Gal G. Predicting deep venous thrombosis in pregnancy: External validation of the 'left' clinical prediction rule. *Journal of Thrombosis and Haemostasis* 2013;11(SUPPL. 3):91. doi: http://dx.doi.org/10.1111/jth.12443
82. Ryan N. Maternal thromboprophylaxis assessment tool: An audit. *Irish Journal of Medical Science* 2019;188(Supplement 7):S53. doi: http://dx.doi.org/10.1007/s11845-019-02053-0
83. Saad A, Tran JP, Stribling S, et al. Performance of risk assessment models for thromboprophylaxis in an obstetric population. *American Journal of Obstetrics and Gynecology* 2018;218(1 Supplement 1):S48-S49.
84. Santos R, Barros VV, Igai AK, et al. Maternal death and Venous Thromboembolism (VTE) in patients admitted in a maternity of high risk: Results pre and post application of a risk score. *Journal of Thrombosis and Haemostasis* 2015;13(SUPPL. 2):679. doi: http://dx.doi.org/10.1111/jth.12993
85. Schoenbeck D, Nicolle A, Newbegin K, et al. The use of a scoring system to guide thromboprophylaxis in a high-risk pregnant population. *Thrombosis* 2011;2011:652796. doi: https://dx.doi.org/10.1155/2011/652796
86. Sellappan KV, Farr R, Hill P. An audit on risk assessment of women at antenatal booking appointment. *International Journal of Gynecology and Obstetrics* 2012;119(SUPPL. 3):S703-S04. doi: http://dx.doi.org/10.1016/S0020-7292%2812%2961724-X

87. Shacaluga A, Wallace P, Rayment R. Abandoning RCOG Guideline 37a: A risk worth taking? *BJOG: An International Journal of Obstetrics and Gynaecology* 2017;124(Supplement 1):142. doi: http://dx.doi.org/10.1111/1471-0528.9_14572
88. Tan EK, Wisdom SJ. Thromboprophylaxis post vaginal delivery: are we forgetting it? Audit on thromboprophylaxis prescription post vaginal births. *Journal of Obstetrics and Gynaecology: The Journal of the Institute of Obstetrics and Gynaecology* 2006;26(1):27-9.
89. Tang Z, Marsden PJ. Risk assessment and management of venous thrombo-embolism in obstetric women. *Archives of Disease in Childhood: Fetal and Neonatal Edition* 2011;96(SUPPL. 1) doi: http://dx.doi.org/10.1136/adc.2011.300163.65
90. Taylor GM, McKenzie CA, Mires GJ. Use of a computerised maternity information system to improve clinical effectiveness: thromboprophylaxis at caesarean section. *Postgraduate Medical Journal* 2000;76(896):354-6.
91. Testa S, Paoletti O, Ronca E, et al. Health care program for thromboembolism prevention in pregnancy. *Pathophysiology of Haemostasis and Thrombosis* 2010;37(SUPPL. 1):A76. doi: http://dx.doi.org/10.1159/000318097
92. Testa S, Passamonti SM, Paoletti O, et al. The pregnancy health-care program: A model for the prevention of venous thromboembolism in pregnancy. *Journal of Thrombosis and Haemostasis* 2013;11(SUPPL. 2):606.
93. Touhami O, Marzouk SB, Bennasr L, et al. Are the Wells Score and the Revised Geneva Score valuable for the diagnosis of pulmonary embolism in pregnancy? *European Journal of Obstetrics, Gynecology, and Reproductive Biology* 2018;221:166-71. doi: https://dx.doi.org/10.1016/j.ejogrb.2017.12.049
94. Usoro E, Sakurai H, Aedla N. Investigation and management of venous thromboembolism in pregnancy. *European Journal of Obstetrics Gynecology and Reproductive Biology* 2019;234:e105. doi: http://dx.doi.org/10.1016/j.ejogrb.2018.08.390
95. Valdre L, Lambertini I, Palareti G, et al. Comparison of strategies for preventing venous thromboembolism in high risk pregnant women according to national and international guidelines: Results of a prospective cohort study. *Blood Transfusion* 2016;14(Supplement 5):s747.
96. Von Hawrylak F. Implementing a venous thromboembolism risk assessment in an abortion service. *European Journal of Contraception and Reproductive Health Care* 2018;23(Supplement 1):43. doi: http://dx.doi.org/10.1080/13625187.2018.1442911
97. Zhang W, Shen J, Sun J-L. Risk scores, prevention, and treatment of maternal venous thromboembolism. *World Journal of Clinical Cases* 2020;8(11):2210-18. doi: https://dx.doi.org/10.12998/wjcc.v8.i11.2210

APPENDIX S3 SIMPLIFIED SUMMARY OF WIDELY EVALUATED GENERIC RAMS, THEIR ASSOCIATED CHARACTERISTICS AND COMPOSITE CLINICAL VARIABLES

Characteristics	Name of VTE risk assessment model			
	RCOG	ACOG	SFOG	Lyon score
General				
Author, year	Royal College of Obstetricians and Gynaecologists, 2015[1]	James et al., 2018[2]	Lindquist et al., 2008[3] and Lindqvist & Hellgren, 2011[4]	Dargaud et al., 2017[5]
Applicable cohort	All pregnant and postpartum women	All pregnant and postpartum women at risk	Pregnant women with moderate-high risk of VTE	Pregnant women with high risk of thrombosis
Design	Risk factor based with cumulative score	Risk factor based	Risk factor based with cumulative score	Risk factor based with cumulative score
Number of VTE risk variables	26	Not specified	23	15
When is pharmacological thromboprophylaxis recommended?	• Score ≥ 4 antenatally (from first trimester)<br>• Score 3 antenatally (from 28 weeks)<br>• Score ≥ 2 postnatally (at least 10 days).<br>• Antenatal hospital admission<br>• Prolonged hospital admission (≥ 3 days) or readmission to hospital within puerperium	• All women with acute VTE during pregnancy, or women with history of thrombosis or those at significant risk of VTE during pregnancy or the postpartum period such as those with thrombophilia	• Very high risk (high dose antepartum and at least 12 weeks postpartum)[a]<br>• Score ≥ 4 (antepartum and 6 weeks postpartum)<br>• Score 3 (after delivery [6 weeks])<br>• Score 2 (after delivery [7 days] or during immobilisation)	• Score ≥ 6 antenatally or postnatally<br>• Score between 3 and 5, from third trimester
**Pre-existing risk factors**				

Previous VTE (personal)	Yes (except a single event related to major surgery)	Yes	Yes	Yes (pregnancy related, CVT or massive PE or VTE in childhood [<16 years]; unprovoked or oestrogen related; transient risk factor induced)
Recurrent VTE	No	Yes	Yes	Yes (personal history; residual venous thrombi with clinical signs of PTS, recent <2 years)
Previous VTE provoked by specific event	Yes (major surgery)	Yes (surgery, trauma or immobility AND additional major thrombotic risk factors)[b]	No	No
Family history of VTE	Yes (unprovoked or estrogen related)	Yes (first degree with thrombophilia)	Yes (first degree <60 years)	Yes (severe or recurrent)
Thrombophilia e.g. Factor V Leiden and Factor II mutations; protein C, protein S and antithrombin deficiency; antiphospholipid syndrome (with or without VTE)	Yes (various forms	Yes (various forms)	Yes (various forms)	Yes (various forms)
Medical comorbidities	Yes (3 points for any individual comorbidities)	No	Yes (inflammatory bowel disease)	No
Age	Yes (>35 years)	No	Yes (>40 years)	Yes (>35 years)
Obesity	Yes (≥30kg/m^2; ≥40kg/m^2)	No	Yes (>28kg/m^2 in early pregnancy)	Yes (≥30kg/m^2)

Parity	Yes (≥3)	No	No	No
Smoker	Yes	No	No	No
Varicose veins	Yes (gross)	No	No	No
Hyperhomocysteinemia	No	No	Yes (homocysteine >8 μmol/L in pregnancy	No
Mechanical heart prosthesis	No	No	Yes	No
Chronic warfarin prophylaxis	No	No	Yes	No
**Obstetric**				
Pre-eclampsia	Yes (current pregnancy)	No	Yes	No
ART/IVF	Yes (antenatal only)	No	No	No
Multiple pregnancy	Yes	No	No	Yes
Caesarean section	Yes (elective/in labour)	No	Yes	No
Mid-cavity or rotational operative delivery	Yes	No	No	No
Prolonged labour (> 24 hours)	Yes	No	No	No
Postpartum haemorrhage	Yes (>1 litre or transfusion)	No	No	No
Preterm birth	Yes (<37 weeks, current pregnancy)	No	No	No
Stillbirth	Yes (current pregnancy)	No	No	No
Abruptio placenta	No	No	Yes	No
**Transient factors**				
Any surgical procedure	Yes (pregnancy or puerperium except immediate repair of the perineum)	No	No	No
Hyperemesis	Yes	No	No	No

Ovarian hyperstimulation syndrome	Yes (first trimester only)	No	No	No
Systemic infection	Yes (current)	No	No	No
Immobility	Yes (current and dehydration)	No	Yes	Yes
**Other**				
'Other risk factors'	No	No	Yes (according to clinical decision)	No

[a] Thromboprophylaxis initiated as early as possible (sometimes before pregnancy). Only women with antithrombin deficiency, chronic warfarin prophylaxis, recurrent VTE, antiphospholipid syndrome with VTE, and those with mechanical heart prosthesis are included in this group

[b] First-degree relative with a history of a thrombotic episode, or other major thrombotic risk factors (e.g., obesity, prolonged immobility, caesarean delivery

ACOG, American College of Obstetricians and Gynaecologists; ART/IVF Assisted reproductive technology/ In vitro fertilization; RCOG, Royal College of Obstetricians and Gynaecologists; SFOG, Swedish Society of Obstetrics and Gynecology; VTE, venous thromboembolism

REFERENCES (APPENDIX S3)

1. Royal College of Obstetricians & Gynaecologists. Thrombosis and Embolism during Pregnancy and the Puerperium, Reducing the Risk (Green-top Guideline No. 37a) 2015 [Available from: https://www.rcog.org.uk/en/guidelines-research-services/guidelines/gtg37a/ accessed January 2022.
2. James A, Birsner M, Kaimal A, et al. ACOG Practice Bulletin No. 196. Summary: thromboembolism in pregnancy. *Obstet Gynecol* 2018;132:243-8.
3. Lindqvist PG, Torsson J, Almqvist A, et al. Postpartum thromboembolism: severe events might be preventable using a new risk score model. *Vascular Health and Risk Management* 2008;4(5):1081-7.
4. Lindqvist PG, Hellgren M. Obstetric thromboprophylaxis: the Swedish guidelines. *Advances in Hematology* 2011;2011:157483. doi: https://dx.doi.org/10.1155/2011/157483
5. Dargaud Y, Rugeri L, Fleury C, et al. Personalized thromboprophylaxis using a risk score for the management of pregnancies with high risk of thrombosis: a prospective clinical study. *Journal of Thrombosis and Haemostasis* 2017;15(5):897-906.
