# Supplementary material for: Risk assessment models for venous thromboembolism in pregnancy and in the puerperium: a systematic review
Source: BMJ Open. 2022 Oct 12;12(10):e065892. doi: 10.1136/bmjopen-2022-065892 (PMC9562726; doi:10.1136/bmjopen-2022-065892)
Supplement: Author's manuscript [file bmjopen-2022-065892.draft_revisions.pdf]

BMJ Open is committed to open peer review. As part of this commitment we make the peer review history of every article we publish publicly available.

When an article is published we post the peer reviewers' comments and the authors' responses online. We also post the versions of the paper that were used during peer review. These are the versions that the peer review comments apply to.

The versions of the paper that follow are the versions that were submitted during the peer review process. They are not the versions of record or the final published versions. They should not be cited or distributed as the published version of this manuscript.

BMJ Open is an open access journal and the full, final, typeset and author-corrected version of record of the manuscript is available on our site with no access controls, subscription charges or pay-per-view fees (http://bmjopen.bmj.com).

If you have any questions on BMJ Open's open peer review process please email info.bmjopen@bmj.com

BMJ Open

# Risk assessment models for venous thromboembolism in pregnancy and in the puerperium: a systematic review

| | |
|---|---|
| Journal: | *BMJ Open* |
| Manuscript ID | bmjopen-2022-065892 |
| Article Type: | Original research |
| Date Submitted by the Author: | 23-Jun-2022 |
| Complete List of Authors: | Pandor, Abdullah; The University of Sheffield, ScHARR<br>Daru, Jahnavi; Queen Mary University of London, Institute of Population Health Sciences<br>Hunt, Beverley; Guy's and St Thomas' NHS Foundation Trust, Department of Haematology<br>Rooney, Gill; The University of Sheffield, ScHARR<br>Hamilton, Jean; The University of Sheffield, ScHARR<br>Clowes, Mark; The University of Sheffield, ScHARR<br>Goodacre, Steve; The University of Sheffield, ScHARR<br>Nelson-Piercy, Catherine; Guy's and St Thomas' NHS Foundation Trust<br>Davis, Sarah; The University of Sheffield, ScHARR |
| Keywords: | OBSTETRICS, HAEMATOLOGY, Maternal medicine < OBSTETRICS, Anticoagulation < HAEMATOLOGY |

SCHOLARONE™
Manuscripts

*I, the Submitting Author has the right to grant and does grant on behalf of all authors of the Work (as defined in the below author licence), an exclusive licence and/or a non-exclusive licence for contributions from authors who are: i) UK Crown employees; ii) where BMJ has agreed a CC-BY licence shall apply, and/or iii) in accordance with the terms applicable for US Federal Government officers or employees acting as part of their official duties; on a worldwide, perpetual, irrevocable, royalty-free basis to BMJ Publishing Group Ltd ("BMJ") its licensees and where the relevant Journal is co-owned by BMJ to the co-owners of the Journal, to publish the Work in this journal and any other BMJ products and to exploit all rights, as set out in our licence.*

*The Submitting Author accepts and understands that any supply made under these terms is made by BMJ to the Submitting Author unless you are acting as an employee on behalf of your employer or a postgraduate student of an affiliated institution which is paying any applicable article publishing charge ("APC") for Open Access articles. Where the Submitting Author wishes to make the Work available on an Open Access basis (and intends to pay the relevant APC), the terms of reuse of such Open Access shall be governed by a Creative Commons licence – details of these licences and which Creative Commons licence will apply to this Work are set out in our licence referred to above.*

*Other than as permitted in any relevant BMJ Author's Self Archiving Policies, I confirm this Work has not been accepted for publication elsewhere, is not being considered for publication elsewhere and does not duplicate material already published. I confirm all authors consent to publication of this Work and authorise the granting of this licence.*

For peer review only

**Complete manuscript title:**

# Risk assessment models for venous thromboembolism in pregnancy and in the puerperium: a systematic review

**Short title: Predicting VTE in pregnancy**

**Authors:**

Abdullah Pandor, ScHARR, The University of Sheffield, Sheffield, UK

Jahnavi Daru, Institute of Population Health Sciences, Queen Mary University of London, UK

Beverley J Hunt, Guy's and St Thomas' NHS Foundation Trust, London, UK

Gill Rooney, ScHARR, The University of Sheffield, Sheffield, UK

Jean Hamilton, ScHARR, The University of Sheffield, Sheffield, UK

Mark Clowes, ScHARR, The University of Sheffield, Sheffield, UK

Steve Goodacre, ScHARR, The University of Sheffield, Sheffield, UK

Catherine Nelson-Piercy, Guy's and St Thomas' NHS Foundation Trust, London, UK

Sarah Davis, ScHARR, The University of Sheffield, Sheffield, UK

**Corresponding author:** Abdullah Pandor, BSc (Hons), MSc, ScHARR, The University of Sheffield, Regent Court, 30 Regent Street, Sheffield, S1 4DA, telephone +44 (0) 114 222 0778, email: a.pandor@sheffield.ac.uk

**Word count: 3576**



For peer review only - http://bmjopen.bmj.com/site/about/guidelines.xhtml

## ABSTRACT

### OBJECTIVES

To assess the comparative accuracy of risk assessment models (RAMs) to identify women during pregnancy and the early postnatal period who are at increased risk of VTE.

### DESIGN

Systematic review following Preferred Reporting Items for Systematic Reviews and Meta-Analyses guidelines.

### DATA SOURCES

MEDLINE, EMBASE, Cochrane Library and two research registers were searched until February 2021.

### ELIGIBILITY CRITERIA

All validation studies that examined the accuracy of a multivariable RAM (or scoring system) for predicting the risk of developing VTE in women who are pregnant or in the puerperium (within 6-weeks post-delivery).

### DATA EXTRACTION AND SYNTHESIS

Two authors independently selected and extracted data. Risk of bias was appraised using PROBAST tool. Data were synthesised without meta-analysis.

### RESULTS

Seventeen studies, comprising 19 externally validated RAMs and one internal validated model met the inclusion criteria. The most widely evaluated RAMs were the RCOG guidelines (6 studies), ACOG guidelines (2 studies), SFOG guidelines (2 studies) and the Lyon score (2 studies). In general,




For peer review only - http://bmjopen.bmj.com/site/about/guidelines.xhtml

estimates of sensitivity and specificity were highly variable with sensitivity estimates ranging from 0% to 100% for RAMs that were applied to antepartum women to predict antepartum or postpartum VTE and 0% to 100% for RAMs applied postpartum to predict postpartum VTE. Specificity estimates were similarly diverse ranging from 28% to 98% and 5% to 100%, respectively.

**CONCLUSIONS**

Available data suggest that external validation studies have weak designs and limited generalisability, so estimates of prognostic accuracy are very uncertain.

**PROSPERO REGISTRATION**

Abdullah Pandor, Steve Goodacre, Gill Rooney, Mark Clowes, Sarah Davis. A systematic review of risk assessment models for venous thromboembolism in pregnancy and in the puerperium. PROSPERO 2020 CRD42020221094 Available from:
https://www.crd.york.ac.uk/prospero/display_record.php?ID=CRD42020221094

**KEYWORDS**

Pregnancy, postpartum period, risk assessment, systematic review, venous thromboembolism

For peer review only

## ARTICLE SUMMARY

### Strengths and limitations of this study

- A number of risk assessment models for venous thromboembolism (VTE) in pregnancy & puerperium have been developed using a variety of methods and based on a variety of predictor variables.
- This systematic review provides a comprehensive review of risk assessment models for predicting the risk of developing VTE in women who are pregnant or in the puerperium (within 6 weeks post-delivery).
- The newly developed PROBAST tool was used to evaluate the risk of bias and applicability of the available evidence
- Heterogeneity in the included studies (participants, inclusion criteria, clinical condition, outcome definition and measurement) and variable reporting of items precluded meta-analysis.
- Limitations of the existing evidence and areas of future research are highlighted.



For peer review only - http://bmjopen.bmj.com/site/about/guidelines.xhtml

## INTRODUCTION

Venous thromboembolism (VTE) remains an important cause of maternal morbidity and mortality in the developed world.[1] Whilst uncommon, VTE complications can occur at a rate of 1 to 2 per 1000 deliveries and can develop at any time during pregnancy.[2-4] The risks substantially increase during the post-partum period (6 weeks post-delivery)[5] and can be as high as 60-fold in some individuals compared with age-matched non-pregnant women.[6] Preventative treatment with low-dose anticoagulation (thromboprophylaxis) has the potential to reduce the risk of symptomatic and asymptomatic VTE in pregnancy and the postpartum period.[5] Consequently, various prominent international guidelines recommend targeted thromboprophylaxis for pregnant and puerperal women deemed to be at high risk of VTE.[5, 7-13] However, these expert-based consensus guidelines vary substantially with regards to the threshold of risk (based on certain risk factors) and the timing, dose and duration of pharmacological thromboprophylaxis.

Risk assessment models (RAMs) have been developed to help stratify the risk of VTE during pregnancy and the early postnatal period. These models use clinical information from the patient's history and examination to identify those with an increased risk of developing VTE who are most likely to benefit from pharmacological thromboprophylaxis. Inappropriate use of VTE prophylaxis may not reduce VTE rates and may cause unnecessary harm especially through bleeding and bruising.[14] Whilst RAMs could improve the ratio of benefit to risk and benefit to cost, it is unclear which VTE RAM are best  applied to guide decision-making for thromboprophylaxis in clinical practice and thereby optimise patient care.



For peer review only - http://bmjopen.bmj.com/site/about/guidelines.xhtml

The aim of this systematic review was to identify primary validation studies and determine the accuracy of individual RAMs that identify pregnant and postpartum women at increased risk of developing VTE who could be selected for thromboprophylaxis.

## METHODS

A systematic review was undertaken in accordance with the general principles recommended in the Preferred Reporting Items for Systematic Reviews and Meta-Analyses (PRISMA) statement.[15] This review was part of a larger project on Thromboprophylaxis in pregnancy and after delivery[16] and was registered on the International Prospective Register of Systematic Reviews (PROSPERO) database (CRD42020221094).

### Eligibility criteria

All studies evaluating the accuracy (e.g. sensitivity, specificity, C-statistic) of a multivariable RAM (or scoring system) for predicting the risk of developing VTE were eligible for inclusion. We primarily sought and selected studies that included validation of the model in a group of patients that were not involved in the development of the prediction model. Although the included studies could have reported derivation of the model (for internal validation), we only used the external validation data to estimate accuracy, where appropriate. The study population of interest in our review consisted of pregnant and postpartum (within 6 weeks post-delivery) women who are at increased risk of developing a VTE and receiving care in both hospital, community, and primary care settings. Studies that focussed on non-pregnant women were excluded as these patient groups have VTE risk profiles that differ markedly from the obstetric population.

### Data sources and searches



For peer review only - http://bmjopen.bmj.com/site/about/guidelines.xhtml

For peer review only

Several electronic databases including MEDLINE (with MEDLINE In-process and Epub Ahead of Print), EMBASE and the Cochrane Library were searched from inception to February 2021. The search strategy used free text and thesaurus terms and combined synonyms relating to the condition (e.g. VTE in pregnant and postpartum women) with risk prediction modelling terms.[17] No language or date restrictions were used. Searches were supplemented by hand-searching the reference lists of all relevant studies (including existing systematic reviews); forward citation searching of included studies; contacting key experts in the field; and undertaking targeted searches of the World Wide Web using the Google search engine. Further details on the search strategy can be found in the Appendix S1 (supplementary information).

**Study selection**

All titles were examined for inclusion by one reviewer (GR) and any citations that clearly did not meet the inclusion criteria (e.g. non-human, unrelated to VTE in pregnancy and the puerperium) were excluded (for quality assurance a random subset of 20% was checked by a second reviewer). All abstracts and full text articles were then examined independently by two reviewers (GR and AP). Any disagreements in the selection process were resolved through discussion or if necessary, arbitration by a third reviewer (JD) or the wider group (BH, CNP, SG) and included by consensus.

**Data extraction and quality assessment**

For eligible studies, data relating to study design, methodological quality and outcomes were extracted by one reviewer (GR) into a standardised data extraction form and independently checked for accuracy by a second reviewer (AP). Any discrepancies were resolved through discussion, or if this was unsuccessful, a third reviewer's opinion was sought (JD). Where

For peer review only



For peer review only - http://bmjopen.bmj.com/site/about/guidelines.xhtml

multiple publications of the same study were identified, data were extracted and reported as a single study.

The methodological quality of each included study was assessed using PROBAST (Prediction model Risk Of Bias ASsessment Tool).[18, 19] This instrument includes four key domains: participants (e.g., study design and patient selection), predictors (e.g., differences in definition and measurement of the predictors), outcome (e.g., differences related to the definition and outcome assessment), and statistical analysis (e.g., sample size, choice of analysis method and handling of missing data). Each domain is assessed in terms of risk of bias and the concern regarding applicability to the review (first three domains only). To guide the overall domain-level judgement about whether a study is at high, low or an unclear (in the event of insufficient data in the publication to answer the corresponding question) risk of bias, sub-domains within each domain include several signalling questions to help judge with bias and applicability concerns. An overall risk of bias for each individual study was defined as low risk when all domains were judged as low; and high risk of bias when one or more domains were considered as high. Studies were assigned an unclear risk of bias if one or more domains were unclear, and all other domains were low.

**Data synthesis and analysis**

Due to significant levels of heterogeneity between studies (study design, participants, inclusion criteria) and variable reporting of items, a meta-analysis was not considered possible. As a result, a pre-specified narrative synthesis approach[20, 21] was undertaken, with data being summarised in tables with accompanying narrative summaries that included a description of the included variables, statistical methods and performance measures (e.g. sensitivity, specificity and C-statistic [a value between 0.7 to 0.8 and >0.8 indicated good and



For peer review only - http://bmjopen.bmj.com/site/about/guidelines.xhtml

excellent discrimination, respectively; and values <0.7 were considered weak]),[22] where applicable. All analyses were conducted using Microsoft Excel 2010 (Microsoft Corporation, Redmond, WA, USA).

**Patient and public involvement**

Patients and the public were not involved in the design or conduct of this systematic review.

## RESULTS

### Study flow

**Figure 1** summarises the process of identifying and selecting relevant literature. Of the 2268 citations identified, 16 studies[23-38] investigating 19 unique externally validated RAMs met the inclusion criteria. Only one of these studies[35] presented data on model development and external validation (this study used UK Clinical Practice Research Data linked to Hospital Episode Statistics to develop a risk prediction model and externally validated using Swedish medical birth registry data). The remaining studies focused on external validation with no description of the initial derivation methodology.[23-34, 36-38] Due to the lack of model derivation studies with external validation, we also identified and included one internal validation study for completeness (i.e. prediction model development without external validation).[39] This study used a boot-strap validation approach to capture optimism in model performance[40, 41] when applied to similar future patients. Most of the full-text articles (n=97) were excluded primarily based on not using a RAM for predicting the risk of developing VTE during pregnancy or the puerperium, having no useable or relevant outcome data or an inappropriate study design (e.g., reviews, commentaries, or study protocols). A full list of excluded studies with reasons for exclusion is provided in Appendix S2 (supplementary information).



For peer review only - http://bmjopen.bmj.com/site/about/guidelines.xhtml

**[INSERT FIGURE 1]**

## Study and patient characteristics

The design and participant characteristics of the 17 included studies are summarised in **Table 1**. All studies were published between 2000 and 2020 and were undertaken in North America (n=4),[24, 37-39] Southeast Asia (n=1),[33] Europe (n=10),[23, 25-30, 32, 34, 36] South America (n=1),[31] and one study was multi-country.[35] Sample sizes ranged from 52[31] to 662,387[35] patients in 14 observational cohort studies (6 prospective[25, 27, 28, 31, 33, 36] [all single centre] and 8 retrospective[24, 26, 29, 30, 34, 35, 37, 39] [2 of which were multicentre] in design). Sample sizes in two, single centre case control studies[32, 38] ranged from 76[38] to 2421[32] patients and one study used a non-randomised multicentre study design.[23] The mean age ranged from 27.8 years[39] to 34 years[25, 29] (not reported in 7 studies).[24, 27, 32, 34, 36-38]

The majority of studies were conducted across antenatal and postnatal periods,[23, 27-29, 31, 34, 36, 38] or postpartum period only[24-26, 30, 32, 33, 35, 37, 39] and generally included women at increased risk of VTE.[23-25, 28, 29, 31-33, 38, 39] One study excluded women with a history of VTE[35] and six studies[26, 27, 30, 34, 36, 37] included all pregnant women who delivered. Thromboprophylaxis was employed in about half (n=9)[23, 25, 28-31, 33, 35, 36] of the studies, with the proportion receiving thromboprophylaxis ranging from 3%[35] to 100%.[23, 28] The remaining studies did not report data on thromboprophylaxis use.



For peer review only - http://bmjopen.bmj.com/site/about/guidelines.xhtml

## Table 1: Study and population characteristics

| Author, year | Country | Design | Single/ multi-centre | Sample size | Population | Period | Mean age (years) | VTE prophylaxis | RAMs evaluated | Target condition, definition (risk period) | Incidence |
|---|---|---|---|---|---|---|---|---|---|---|---|
| Antepartum and postpartum following vaginal and caesarean delivery | | | | | | | | | | | |
| Bauersachs et al., 2007[23] | Germany | P,NRS | Multi | 810 | Women at increased risk of VTE (due to thromboembolic status and prior VTE) | March 1999 to December 2002 | 30.8 | 100% | •EThIG | Antepartum and postpartum VTE, symptomatic (NR) | 0.62% (antepartum: 0.25%; postpartum: 0.37%) |
| Chauleur et al., 2008[27] | France | P,CS | Single | 2685 | All women who delivered | July 2002-June 2003 | NR (median, 29) | NR | •STRATHEGE | Antepartum and postpartum VTE (NR) | 0.34% (antepartum: 0.19%; postpartum: 0.15%) |
| Dargaud et al., 2017[28] | France | P,CS | Single | 445 | Women at increased risk of VTE (due to thromboembolic status and prior VTE) | January 2005-January 2015 | 33 | 100% | •Lyon | Antepartum and postpartum VTE, not defined (pregnancy and 3 months postpartum) | 1.35% |
| Dargaud et al., 2005[29] | France | R,CS | Single | 116 | Women at increased risk of VTE (due to thromboembolic status and prior VTE) | 2001-2003 | 34 | 53% | •Lyon | Antepartum and postpartum VTE, not defined (NR) | 0.86% (antepartum only) |
| Hase et al., 2018[31] | Brazil | P,CS | Single | 52 | Hospitalised pregnant women with cancer | 1 December 2014 to 31 July 2016 | 31 | 57.7% | •RCOG (modified) | Antepartum and postpartum VTE, not defined (pregnancy and 3 months postpartum) | Unable to estimate - no VTE |
| Shacaluga et al., 2019[34] (correspondence) | Wales | R,CS | Single | 42,000 | All managed pregnancies | 2009-2015 | NR | NR | •All Wales •RCOG | Antepartum and postpartum VTE, not defined, (NR) | 0.08% (antepartum: 0.04%; postpartum: 0.04%) |
| Testa et al., 2015[36] | Italy | P,CS | Single | 1719 | All pregnant women enrolled in Pregnancy Healthcare Program | January 2008 to December 2010 | NR (median 33) | 4.6% | •Novel (Testa) | Antepartum and postpartum VTE (NR) | Unable to estimate - no VTE |
| Weiss et al., 2000[38] | USA | CC | Single | 19 cases: 57 control[a] | Women with (confirmed cases) and without (unmatched control) VTE | 1987-1998 | NR | NR | •Novel (Weiss) | Antepartum and postpartum VTE, not defined (pregnancy and 6 weeks postpartum) | - |
| Postpartum only following vaginal and caesarean delivery | | | | | | | | | | | |
| Chau et al., 2019[26] | France | R,CS | Single | 1069 (time period 2012: 557; 2015: 512) | All women who delivered | February-April 2012 and February-April 2015 | 2012: 29 2015: 29 | NR | •Novel (Chau) | Postpartum VTE, not defined (8 weeks) | 2012: 0.18% 2015: 0.20% |
| Ellis-Kahana et al., 2020[39b] | USA | R,CS | Multi | 83,500 | All obese women (BMI >30kg/m$^2$) who delivered | 2002-2008 | 27.8 | NR | •Novel (Ellis-Kahana) | Postpartum VTE (NR) | 0.13% |
| Gassmann et al., 2020[30] | Switzerland | R,CS[c] | Single | 344 | All women who delivered | 1-31 January 2019 | 32.2 | 24% | •RCOG •ACOG •ACCP | Postpartum VTE, not defined (3 months) | Unable to estimate - no VTE |



For peer review only - http://bmjopen.bmj.com/site/about/guidelines.xhtml

| | | | | | | | | | | | |
|---|---|---|---|---|---|---|---|---|---|---|---|
| | | | | | | | | | •ASH | | |
| Lindqvist et al., 2008[32] | Sweden | CC | Single | 37 cases: 2384 control | All women with (confirmed cases) and without (unselected population-based control) VTE | 1990-2005 | NR | NR | •SFOG (Swedish guidelines) | Postpartum VTE (NR) | - |
| Sultan et al., 2016[35] | England (derivation)[d] and, Sweden (validation) | R,CS | Multi | 662,387 (validation cohort)[d] | All women (with no history of VTE) who delivered | 1 July 2005 to 31 December 2011 | 30.32 | 3% | •Novel (Sultan) •RCOG[d] •SFOG (Swedish Guidelines) | Postpartum VTE (6 weeks) | 0.08% (validation cohort) |
| Tran et al., 2019[37] | USA | R,CS | Single | 6094 | All women who delivered after 14 weeks | 01 January 2015 to 31 December 2016 | NR | NR | •RCOG •Padua •Caprini | Postpartum VTE (6 months) | 0.05% |
| Postpartum following caesarean delivery | | | | | | | | | | | |
| Binstock & Larkin, 2019 (abstract)[24] | USA | R,CS | Single | 2875 | Postpartum women following CD | 2011 | NR | NR | •Novel (Binstock) •RCOG | Postpartum VTE, not defined (NR) | 0.38% |
| Cavazza et al., 2012[25] | Italy | P,CS | Single | 501 | Postpartum women following CD | 2007-2009 | 34 | 53.5% | •Novel (Cavazza) | Postpartum VTE, symptomatic, not defined (90 days) | 0.20% |
| Lok et al., 2019[33] | Hong Kong | P,CS | Single | 859 | Postpartum women following CD | May 2017 to April 2018 | 32.9 | 3.3% | •Novel (Lok) •RCOG •ACOG | Postpartum VTE, symptomatic, not defined (NR) | Unable to estimate - no VTE |

Abbreviations: ACCP, American College of Chest Physicians; ACOG, American College of Obstetricians and Gynaecologists; ASH, American Society of Hematology; CC, case -control; CD, caesarean delivery; CS, cohort study; NR, not reported; NRS, non-randomised study; P, prospective; R, retrospective; RCOG, Royal College of Obstetricians and Gynaecologists; SFOG, Swedish Society of Obstetrics and Gynecology; VTE, venous thromboembolism

[a] Retrospective case-control study of pregnant and postpartum women but data reported for antepartum period only due to low number of postpartum VTE events (n=2)

[b] Internal validation study (i.e. prediction model development without external validation)

[c] Prospective cohort study with retrospective analysis, thus classified as retrospective cohort study

[d] RCOG was applied to an English derivation cohort, n=433,353, incidence, 0.07% (312 events)

For peer review only



For peer review only - http://bmjopen.bmj.com/site/about/guidelines.xhtml

### VTE definition and case ascertainment

Only a few studies[23, 27, 32, 36] defined the VTE endpoint (DVT and or PE) as being confirmed by objective testing. Of the remainder, three studies[35, 37, 39] had no objective confirmation of VTE and 10 studies[24-26, 28-31, 33, 34, 38] did not report the methods for diagnosis confirmation. Although 9 studies[23, 24, 27, 29, 32-34, 36, 39] did not report the VTE risk period, the majority of the remaining studies utilised the RAMs to predict the occurrence of VTE up to 3 months after delivery.[25, 28, 30, 31] Despite differences in study design, study participants, definitions, different criteria for the use of thromboprophylaxis and differences between doses of low molecular weight heparin (LMWH), the reported overall incidence of VTE in pregnancy and the puerperium was <1.3%.

### RAMs

The studies included in this review evaluated 19 externally validated RAMs[23-38] and one internally validated risk model.[39] While most RAMs focused solely on the estimate of thromboembolic risk, RAMs varied in design, structure, threshold, dosage and duration for pharmacological prophylaxis. In addition, the individual predictors and their weighting varied markedly between RAMs. The most commonly used tools were the Royal College of Obstetricians and Gynaecologists (RCOG) guidelines (6 studies),[24, 30, 33-35, 37] American College of Obstetricians and Gynaecologists (ACOG) guidelines (2 studies),[30, 33] Swedish Society of Obstetrics and Gynecology (SFOG) guidelines (2 studies)[32, 35] and the Lyon score (2 studies).[28, 29] A simplified summary of their associated characteristics and composite clinical variables is provided in Appendix S3 (supplementary information).

### Risk of bias and applicability assessment



For peer review only - http://bmjopen.bmj.com/site/about/guidelines.xhtml

The overall methodological quality of the 17 included studies is summarised in **Table 2 and Figure 2**. The methodological quality of the included studies was variable, with most studies having high or unclear risk of bias in at least one item of the PROBAST tool. The main risk of bias limitations was related to patient selection factors (arising from retrospective data collection,[24, 26, 29, 30, 32, 34, 37-39] unclear exclusions/ incomplete patient enrolment,[24, 26, 27, 31-34, 36, 38, 39] or unclear criteria for patients receiving VTE prophylaxis)[23, 30, 35]; predictor and outcome bias (due to a general lack of details on the definition[24-26, 28-31, 33, 34, 38] and methods of outcome determination[24, 26, 28-31, 33, 34, 37-39] and whether all predictors were available at the models intended time of use[23, 24, 29, 31, 32, 34, 36-39] or influenced by the outcome measurement)[23-28, 30-39] and analysis factors (low event rates,[23-31, 33-37, 39] unclear handling of missing data,[23-29, 31-34, 36-39] and failure in reporting relevant performance measures such as calibration and discrimination.[23-34, 36-38]

Assessment of applicability to the review question led to the majority of studies being classed either as unclear (n=13)[23, 26-30, 32, 34-39] or high (n=4)[24, 25, 31, 33] risk of inapplicability. These assessments were generally related to patient selection (highly selected study populations e.g. selected women at increased risk of VTE, caesarean delivery only, single disease pathologies, single site settings), predictors (inconsistency in definition, assessment or timing of predictors) and outcome determination.



For peer review only - http://bmjopen.bmj.com/site/about/guidelines.xhtml

# Table 2. Summary of each study's risk of bias and applicability concern using the PROBAST tool – Review authors' judgements

| Author, year | Risk of Bias | | | | Applicability | | | Overall | |
|---|---|---|---|---|---|---|---|---|---|
| | Participant selection | Predictors | Outcome | Analysis | Participant selection | Predictors | Outcome | Risk of bias | Applicability |
| Bauersachs et al., 2007[23] | ? | ? | + | - | ? | ? | + | - | ? |
| Binstock 2019[24] | ? | ? | ? | - | - | ? | ? | - | - |
| Cavazza 2012[25] | - | ? | ? | - | - | + | ? | - | - |
| Chau 2019[26] | ? | ? | ? | - | ? | ? | ? | - | ? |
| Chauleur 2008[27] | ? | ? | ? | - | ? | ? | ? | - | ? |
| Dargaud 2017[28] | ? | ? | ? | - | ? | ? | ? | - | ? |
| Dargaud et al., 2005[29] | - | ? | ? | - | ? | + | ? | - | ? |
| Ellis-Kahana 2020[39] | - | ? | ? | - | ? | ? | ? | - | ? |
| Gassmann et al., 2020[30] | ? | ? | ? | - | ? | ? | ? | - | ? |
| Hase et al., 2018[31] | ? | ? | ? | - | - | ? | ? | - | - |
| Lindqvist et al., 2008[32] | - | ? | ? | - | ? | ? | ? | - | ? |
| Lok et al., 2019[33] | ? | ? | - | - | - | + | ? | - | - |
| Shacaluga et al., 2019[34] | - | ? | ? | - | ? | ? | ? | - | ? |
| Sultan et al., 2016[35] | - | ? | + | + | + | ? | + | - | ? |
| Testa et al., 2015[36] | ? | ? | ? | - | ? | ? | ? | - | ? |
| Tran et al., 2019[37] | - | ? | ? | - | ? | ? | ? | - | ? |
| Weiss & Bernstein, 2000[38] | - | ? | ? | - | ? | ? | ? | - | ? |

+ indicates low risk of bias/low concern regarding applicability; –, indicates high risk of bias/high concern regarding applicability; and ? indicates unclear risk of bias/unclear concern regarding applicability



For peer review only - http://bmjopen.bmj.com/site/about/guidelines.xhtml

[INSERT FIGURE 2]

**Predictive performance of VTE RAMs (summary of results)**

**Table 3** and **Table 4** shows the sensitivity and specificity of RAMs that were applied to antepartum women to predict antepartum or postpartum VTE or applied postpartum to predict postpartum VTE, respectively, with the results grouped by RAM. However, any meaningful comparisons between these alone is difficult, without considering the models' corresponding discrimination and calibration metrics, which were not universally reported. Only one external validation study considered model discrimination and calibration. In this study by Sultan et al.,[35] their recalibrated novel risk prediction model (also known as the Maternity Clot Risk) provided good discrimination and was able to discriminate postpartum women with and without VTE in the external Swedish cohort with a C statistic of 0.73 (95% CI: 0.71 to 0.75), and calibration, of observed and predicted VTE risk, close to ideal (calibration slope of 1.11 [95% CI: 1.01 to 1.20]). In the remaining studies, interpretation was further limited by marked heterogeneity, which was exacerbated when different thresholds were reported by different studies evaluating the same model. In general, model accuracy was generally poor, with high sensitivity usually reflecting a threshold effect, as indicated by corresponding low specificity values (and vice versa).



For peer review only - http://bmjopen.bmj.com/site/about/guidelines.xhtml

## Table 3. Performance of RAMs applied antepartum to predict VTE

| Risk assessment models | Threshold or cut-off | Endpoint | Data source | Performance measures | | | | | |
|---|---|---|---|---|---|---|---|---|---|
| | | | | TP | FP | FN | TN | Sensitivity (95% CI) | Specificity (95% CI) |
| **Predicting either antepartum or postpartum VTE** | | | | | | | | | |
| All Wales (1 study) | NR | VTE | Shacaluga et al., 2019[34] | 25 | NR | 9 | NR | 0.74 (0.57, 0.85) | NR |
| EThIG (1 study) | High/very high risk | VTE | Bauersachs et al., 2007[23] | 5 | 580 | 0 | 225 | 1.00 (0.57, 1) | 0.28 (0.25, 0.31) |
| Lyon (2 studies) | Risk Score ≥3 | VTE | Dargaud et al., 2017[28] | 5 | 282 | 1 | 157 | 0.83 (0.44, 0.97) | 0.36 (0.31, 0.4) |
| Lyon | Risk Score ≥3 | VTE | Dargaud et al., 2005[29] | 1 | 56 | 0 | 59 | 1.00 (0.21, 1) | 0.51 (0.42, 0.6) |
| RCOG (modified) (1 study) | Risk Score ≥3 | VTE | Hase et al., 2018[31] | 0 | 34 | 0 | 18 | unable to estimate - no VTE | 0.35 (0.23, 0.48) |
| STRATHEGE | Risk Score ≥3 | VTE | Chauleur et al., 2008[27] | 0 | 54 | 9 | 2622 | 0.00 (0, 0.3) | 0.98 (0.97, 0.99) |
| Testa 2015 (1 study) | Risk Score ≥2.5 | VTE | Testa et al., 2015[36] | 0 | 85 | 0 | 1634 | unable to estimate - no VTE | 0.95 (0.94, 0.96) |
| **Predicting antepartum VTE** | | | | | | | | | |
| EThIG (1 study) | High/very high risk | VTE | Bauersachs et al., 2007[23] | 2 | 583 | 0 | 225 | 1.00 (0.34, 1) | 0.28 (0.25, 0.31) |
| Lyon (1 study) | Risk Score ≥3 | VTE | Dargaud et al., 2017[28] | 1 | 286 | 1 | 157 | 0.50 (0.09, 0.91) | 0.35 (0.31, 0.4) |
| STRATHEGE (1 study) | Risk Score ≥1 | VTE | Chauleur et al., 2008[27] | 0 | 54 | 4 | 2627 | 0.00 (0, 0.49) | 0.98 (0.97, 0.99) |
| Weiss 2000 (1 study) | Risk Score ≥2 | VTE | Weiss et al., 2000[38] | 4 | 3 | 15 | 54 | 0.21 (0.09, 0.43) | 0.95 (0.86, 0.98) |
| **Predicting postpartum VTE** | | | | | | | | | |
| EThIG (1 study) | High/very high risk | VTE | Bauersachs et al., 2007[23] | 3 | 582 | 0 | 225 | 1.00 (0.44, 1) | 0.28 (0.25, 0.31) |
| Lyon (1 study) | Risk Score ≥3 | VTE | Dargaud et al., 2017[28] | 4 | 283 | 0 | 158 | 1.00 (0.51, 1) | 0.36 (0.31, 0.4) |
| STRATHEGE (1 study) | Risk Score ≥1 | VTE | Chauleur et al., 2008[27] | 0 | 54 | 5 | 2626 | 0.00 (0, 0.43) | 0.98 (0.97, 0.98) |

Abbreviations: CI, confidence interval; FN, false negative; FP, false positive; NR, not reported; RCOG, Royal College of Obstetricians and Gynaecologists; TN, true negative; TP, true positive; VTE, venous thromboembolism



For peer review only - http://bmjopen.bmj.com/site/about/guidelines.xhtml

## Table 4. Performance of RAMs applied postpartum to predict VTE

| Risk assessment models | Threshold or cut-off | Endpoint | Data source | Performance measures | | | | | |
|---|---|---|---|---|---|---|---|---|---|
| | | | | TP | FP | FN | TN | Sensitivity (95% CI) | Specificity (95% CI) |
| **Predicting postpartum VTE following vaginal and caesarean delivery** | | | | | | | | | |
| ACCP (1 study) | NR | VTE | Gassmann et al., 2020[30] | 0 | 34 | 0 | 310 | unable to estimate - no VTE | 0.90 (0.86, 0.93) |
| ACOG (1 study) | NR | VTE | Gassmann et al., 2020[30] | 0 | 30 | 0 | 314 | unable to estimate - no VTE | 0.91 (0.88, 0.94) |
| ASH (1 study) | NR | VTE | Gassmann et al., 2020[30] | 0 | 0 | 0 | 344 | unable to estimate - no VTE | 1.00 (0.99, 1) |
| Caprini (1 study) | Risk Score ≥2 | VTE | Tran et al., 2019[37] | 3 | 5780 | 0 | 311 | 1.00 (0.44, 1) | 0.05 (0.05, 0.06) |
| Caprini | Risk Score ≥3 | VTE | Tran et al., 2019[37] | 1 | 3066 | 2 | 3025 | 0.33 (0.06, 0.79) | 0.50 (0.48, 0.51) |
| Caprini | Risk Score ≥4 | VTE | Tran et al., 2019[37] | 0 | 1257 | 3 | 4834 | 0.00 (0, 0.56) | 0.79 (0.78, 0.80) |
| Padua (1 study) | Risk Score ≥4 | VTE | Tran et al., 2019[37] | 0 | 50 | 3 | 6041 | 0.00 (0, 0.56) | 0.99 (0.99, 0.99) |
| RCOG (3 studies) | NR | VTE | Gassmann et al., 2020[30] | 0 | 138 | 0 | 206 | unable to estimate - no VTE | 0.60 (0.55, 0.65) |
| RCOG | Risk Score ≥2 | VTE | Tran et al., 2019[37] | 1 | 3837 | 2 | 2254 | 0.33 (0.06, 0.79) | 0.37 (0.36, 0.38) |
| RCOG | ≥2 low risk factors or 1 high risk factor | VTE | Sultan et al., 2016[35] | 197 | 149205 | 115 | 283836 | 0.63 (0.58, 0.68) | 0.66 (0.65, 0.66) |
| SFOG (2 studies) | Risk Score ≥2 | VTE | Lindqvist et al., 2008 | 18 | 111 | 19 | 2273 | 0.49 (0.33, 0.64) | 0.95 (0.94, 0.96) |
| SFOG | ≥2 risk factors | VTE | Sultan et al., 2016[35] | 109 | 41145 | 412 | 620721 | 0.21 (0.18, 0.25) | 0.94 (0.94, 0.94) |
| Chau, 2019 (1 study[a]) | Risk Score ≥3 (2012 data set) | VTE | Chau et al., 2019[26] | 0 | 101 | 1 | 456 | 0.00 (0, 0.79) | 0.82 (0.78, 0.85) |
| Chau, 2019 | Risk Score ≥3 (2015 data set) | VTE | Chau et al., 2019[26] | 0 | 113 | 1 | 393 | 0.00 (0, 0.79) | 0.78 (0.74, 0.81) |
| Ellis-Kahana, 2020 (full model) (1 study[b]) | Risk Score >3 (high risk) | VTE | Ellis-Kahana 2020[39] | 68 | 7942 | 41 | 75449 | 0.62 (0.53, 0.71) | 0.90 (0.90, 0.91) |
| Ellis-Kahana, 2020 (without antepartum thromboembolic disorder) | Risk Score >3 (high risk) | VTE | Ellis-Kahana 2020[39] | 63 | 9926 | 46 | 73465 | 0.58 (0.48, 0.67) | 0.88 (0.88, 0.88) |
| Sultan, 2016 (1 study[c]) | ≥2 risk factors: top 35% (threshold: 7.2 per 10,000 deliveries) | VTE | Sultan et al., 2016[35] | 355 | 231480 | 166 | 430386 | 0.68 (0.64, 0.72) | 0.65 (0.65, 0.65) |
| Sultan, 2016 | ≥2 risk factors: top 25% (threshold: 8.7 per 10,000 deliveries) | VTE | Sultan et al., 2016[35] | 310 | 164976 | 211 | 496890 | 0.60 (0.55, 0.64) | 0.75 (0.75, 0.75) |
| Sultan, 2016 | ≥2 risk factors: top 20% (threshold: 9.8 per 10,000 deliveries) | VTE | Sultan et al., 2016[35] | 278 | 131921 | 243 | 529945 | 0.53 (0.49, 0.58) | 0.80 (0.80, 0.80) |
| Sultan, 2016 | ≥2 risk factors: top 10% (threshold: 14 per 10,000 deliveries) | VTE | Sultan et al., 2016[35] | 185 | 66053 | 336 | 595813 | 0.36 (0.32, 0.40) | 0.90 (0.90, 0.90) |
| Sultan, 2016 | ≥2 risk factors: top 6% (threshold: 18 per 10,000 deliveries) | VTE | Sultan et al., 2016[35] | 158 | 41096 | 363 | 620770 | 0.30 (0.27, 0.34) | 0.94 (0.94, 0.94) |
| Sultan, 2016 | ≥2 risk factors: top 5% (threshold: 19.7 per 10,000 deliveries) | VTE | Sultan et al., 2016[35] | 139 | 32980 | 382 | 628886 | 0.27 (0.23, 0.31) | 0.95 (0.95, 0.95) |
| Sultan, 2016 | ≥2 risk factors: top 1% (threshold: 41.2 per 10,000 deliveries) | VTE | Sultan et al., 2016[35] | 47 | 6576 | 474 | 655290 | 0.09 (0.07, 0.12) | 0.99 (0.99, 0.99) |
| **Predicting postpartum VTE following caesarean delivery only** | | | | | | | | | |
| ACOG (1 study) | Risk Score ≥3 | VTE | Lok et al., 2019[33] | 0 | 0 | 0 | 859 | unable to estimate - no VTE | 1.00 (1, 1) |



For peer review only - http://bmjopen.bmj.com/site/about/guidelines.xhtml

| | | | | | | | | | |
|---|---|---|---|---|---|---|---|---|---|
| RCOG (2 studies) | NR | VTE | Binstock & Larkin, 2019 (abstract)[24] | 11 | 2692 | 0 | 172 | 1.00 (0.74, 1) | 0.06 (0.05, 0.07) |
| RCOG | Risk Score ≥3 | VTE | Lok et al., 2019[33] | 0 | 649 | 0 | 210 | unable to estimate - no VTE | 0.24 (0.22, 0.27) |
| Binstock, 2019 (1 study) | NR | VTE | Binstock & Larkin, 2019 (abstract)[24] | 11 | 2635 | 0 | 229 | 1.00 (0.74, 1) | 0.08 (0.07, 0.09) |
| Cavazza 2012 (1 study) | Moderate/high/very high | VTE | Cavazza et al., 2012[25] | 0 | 268 | 1 | 232 | 0.00 (0, 0.79) | 0.46 (0.42, 0.51) |
| Lok, 2019 (1 study) | Risk Score ≥3 | VTE | Lok et al., 2019[33] | 0 | 28 | 0 | 831 | unable to estimate - no VTE | 0.97 (0.95, 0.98) |

Abbreviations: ACCP, American College of Chest Physicians; ACOG, American College of Obstetricians and Gynaecologists; ASH, American Society of Hematology; CI, confidence interval; FN, false negative; FP, false positive; NR, not reported; RCOG, Royal College of Obstetricians and Gynaecologists; SFOG, Swedish Society of Obstetrics and Gynecology; TN, true negative; TP, true positive; VTE, venous thromboembolism

[a] Data discrepancy in paper – text states analysis included 1069 women: 557 in the 2012 time frame and 512 in the 2015 time frame; however, data in tables suggest 558 women included in the 2012 time frame and 507 in the 2015 time frame.
[b] Internal-validation study. Full risk prediction model: C-statistic, 0.817 (95% CI: 0.768, 0.865) with Hosmer-Lemeshow P-value = 0.297; Model without antepartum thromboembolic disorder: C-statistic, 0.778 (95% CI: 0.729, 0.826) with Hosmer-Lemeshow P-value = 0.114
[c] Sultan et al.,[35] final risk prediction model in external Swedish cohort: C-statistic, 0.73 (95% CI: 0.71 to 0.75) and calibration slope, 1.11 (95% CI: 1.01 to 1.20)

For peer review only



For peer review only - http://bmjopen.bmj.com/site/about/guidelines.xhtml

## DISCUSSION

### Summary of results

This systematic review identified 19 externally validated RAMs (and one internally validated risk model) that aimed to predict the risk of VTE in pregnant and postpartum women and who could be selected for thromboprophylaxis. Although various risk models (based on a variety of predictor variables) are being used, most of these lacked rigorous development and evaluation. The predictive accuracy of the RAMs was highly variable, and the substantial risk of bias concerns and the general lack of methodological clarity and unclear applicability make meaningful comparisons of the evidence difficult.

### Interpretation of results

Despite the development and use of various RAMs to predict the risk of developing VTE in women who are pregnant or in the puerperium (within 6 weeks post-delivery), VTE remains the leading cause of direct maternity mortality in the UK (MBRRACE-UK report 2021). Several explanations for this are possible: the risk assessment tools are inadequate; the application of these tools is incomplete or inaccurate; the underlying VTE risks of the pregnant population (increasing age, BMI and comorbidities) are changing from when the RAMS were developed; or all three problems are operating.

The use of thromboprophylaxis was reported in nine studies[23, 25, 28-31, 33, 35, 36] (ranging from 3%[35] to 100%)[23, 28] This may lead to underestimation of predictive accuracy if a given RAM were to predict VTE events that were subsequently prevented by thromboprophylaxis. In the remaining studies (n=8) where thromboprophylaxis use was not reported (n=8), further analysis of its impact upon the performance of the RAMs was not possible. This also suggests



For peer review only - http://bmjopen.bmj.com/site/about/guidelines.xhtml

that the degree to which thromboprophylaxis reduces the risk of VTE in those who received it cannot be accurately estimated.

**Comparison to the existing literature**

To our knowledge, there are no previous systematic reviews on this topic. However, recently several large registries have been interrogated in an attempt to derive robust prediction rules for this population, albeit with some methodological concerns. Sultan et al.,[35] developed (using a large English-based registry database covering 6% of the population) and validated (using a Swedish national database registry) a risk prediction tool to estimate the absolute risk of VTE in postpartum women according to their individual risk factor combinations. Despite the low incidence of VTE in both cohorts (<0.08%), their model showed good discrimination in the external cohort and poor sensitivity at predicting those at risk of experiencing VTE. In addition, their model lacked some important VTE risk factors (e.g. thrombophilia, antepartum immobilisation), and possibly underestimated the risks due to diagnosis limited to diagnostic coding (e.g. varicose veins, severity of comorbidities) and the use of thromboprophylaxis in both cohorts.[42] Ellis-Kahana et al.,[39] also derived (using a large national database from the US) a risk prediction model for VTE in obese pregnant women and indicated strong discrimination. However, this model still requires external validation.

**Strengths and limitations**

This systematic review has several strengths. It is the first systematic review to evaluate RAMs for predicting the risk of developing VTE in women during pregnant and the puerperium periods, and was conducted with robust methodology in accordance with the PRISMA statement[15] and the protocol was registered with the PROSPERO register. Clinical experts, in addition to the core review team, were involved and consulted throughout as



For peer review only - http://bmjopen.bmj.com/site/about/guidelines.xhtml

advisors and to assess the validity and applicability of research findings during the review processes.

The main limitations of this study related to the observational nature of the studies reviewed and their own limitations. Most of the included risk prediction studies were retrospective cohorts. Retrospective cohort studies of large health database registries are limited by poor data quality and failure to accurately ascertain outcomes and case–control designs are prone to bias including uncontrolled confounding, temporal and selection bias.[43] Conversely, better quality data may be obtained with prospective cohorts, but smaller sample sizes will lack statistical power. In addition, most of the external validation studies evaluated predictive performance of risk models that were not statistically derived (i.e. without model development and internal validation). This process is vital, as risk models with only external validation may be subject to overfitting and optimism.[40] Similarly, the absence of model performance measures such as calibration or discrimination hinders the full appraisal of models.[41]

Due to the high levels of heterogeneity between studies, we were unable to undertake any meta-analysis or statistical examination of the causes of heterogeneity due to the small number of external validation studies per risk model. Potential sources of heterogeneity include variation in study design, the study population, risk model implementation, outcome definition and measurement, and the use of thromboprophylaxis. As a result, we reported descriptive statistics to provide a better understanding of the evidence base applicable to the subject matter, and shortcomings regarding reliability and validity of the data. Finally, assessments on study relevance, information gathering and validity of articles were unblinded and could potentially have been influenced by pre-formed opinions. However, masking is



For peer review only - http://bmjopen.bmj.com/site/about/guidelines.xhtml

resource intensive with uncertain benefits in protecting against bias decisions.[44]

**Implications for policy, practice and future research**

VTE risk assessment is challenging for numerous reasons. Many risk factors for VTE are pre-existing and non-modifiable (such as parity and inherited thrombophilia). These are then often combined with evolving risk factors which can change over the course of a pregnancy or postnatal period. Despite wide scale awareness of VTE being a major contributor to maternal mortality, numerous challenges with VTE risk stratification have been highlighted. In the UK, the MBRRACE-UK report (Saving Lives, Improving Mothers' Care 2018)[45] shows that doctors and midwives find existing risk scoring systems difficult to apply consistently in clinical practice. There is a need for development of a RAM that is simpler and more reproducible. NICE Guidelines on the use of thromboprophylaxis (NG89)[46] concluded that the tool described by Sultan et al.[35] showed poor sensitivity compared to their pre-specified target of 90% sensitivity. However, this high level of sensitivity may not be realistic because there is evidence that only 70% of women having antenatal pulmonary embolism had any identifiable classic risk factors suggesting that sensitivity rates above 70% may not be achievable.[47] In addition, a high sensitivity rate is usually associated with a lower specificity rate and the overall balance of benefits and harms may be undesirable if that means exposing a high proportion of women to thromboprophylaxis.

Despite lack of evidence, many guidelines and clinical care bundles include the use of RAMs to guide VTE prophylaxis. Recently published ACOG guidelines state that the most RAMs have not been validated prospectively in the obstetric population and that current usage of such models is based on extrapolations from non-pregnant women, who differ biologically from pregnant women. The practice bulletin emphasises the need for more research to



For peer review only - http://bmjopen.bmj.com/site/about/guidelines.xhtml

identify optimal models.[37] Although further research is clearly needed the routine use of thromboprophylaxis may present a barrier to generating accurate and precise estimates of the prognostic accuracy of RAMs. Further work to improve RAMs to help stratify the risk of VTE in women who are pregnant or in the puerperium could focus on using decision-analytic modelling to compare the effects, harms and costs of giving thromboprophylaxis to patients with varying risk of VTE. This would allow determination of the risk threshold at which thromboprophylaxis provides optimal overall benefit. Subsequent work to validate these findings would require primary research. Despite the limitations of undertaking accuracy studies in populations where thromboprophylaxis is routinely used, future research could focus on selected higher risk groups who are more likely to benefit from prophylaxis and, with a higher prevalence of VTE, are more amenable to an appropriately powered prospective study. However, given the uncertain benefits and harms of VTE thromboprophylaxis during pregnancy and the postpartum period,[14, 48] risk prediction studies should be undertaken alongside (or as a part of) randomised trials of prophylaxis in targeted groups deemed to be at higher risk of VTE.

## CONCLUSIONS

Currently there are a number of risk assessment models for assessing risk of VTE in pregnancy and the puerperium. Our review has shown that none of these models has been adequately validated and they have limited abilities to detect those at risk of VTE.

For peer review only

## ACKNOWLEDGEMENTS

The authors would like to thank all additional members of the core project group for NIHR131021 for input and commentary throughout the work. We are also indebted to Donna Davis for assistance with logistics and administration.

## COMPETING INTEREST

CNP reports personal fees from Sanofi, and is the lead developer of the RCOG Green Top Guideline on thromboprophylaxis in pregnancy (37a). All other authors declare no competing interests.

## SOURCE OF FUNDING

This study was funded by the United Kingdom National Institute for Health Research Health Technology Assessment Programme (project number 131021). The views expressed in this paper are those of the authors and not necessarily those of the NIHR HTA Programme. Any errors are the responsibility of the authors. The funders had no role in the study design, in the collection, analysis and interpretation of data; in the writing of the manuscript; and in the decision to submit the manuscript for publication.

## DETAILS OF ETHICS APPROVAL

Not applicable, as this is a systematic review of published papers.

## DATA SHARING STATEMENT

All data relevant to the study are included in the article or uploaded as supplementary information



For peer review only - http://bmjopen.bmj.com/site/about/guidelines.xhtml

## AUTHOR CONTRIBUTIONS

AP co-ordinated the review. SD, AP, JD, JH, MC, BH, CNP, and SG were responsible for conception, design and obtaining funding for the study. MC developed the search strategy, undertook searches, and organised retrieval of papers. AP, GR, JH, BH, JD, CNP and SG were responsible for the acquisition, analysis and interpretation of data. BH, JD, CNP and SG helped interpret and provided a methodological, policy and clinical perspective on the data. AP, BH, JD and SD were responsible for the drafting of this paper, although all authors provided comments on the drafts, read and approved the final version. AP is the guarantor for the paper.

## COPYRIGHT LICENCE STATEMENT

The project was funded by the United Kingdom National Institute for Health Research Health Technology Assessment Programme and as such is covered by Crown copyright. The Corresponding Author has the right to grant on behalf of all authors and does grant on behalf of all authors, **a non-exclusive license** on a worldwide basis to BMJ publishing (BMJ) and its Licensees to permit this article (if accepted) to be published by BMJ , as set out in our licence.

For peer review only

## REFERENCES

1. Say L, Chou D, Gemmill A, et al. Global causes of maternal death: a WHO systematic analysis. *Lancet Glob Health*. 2014;2(6):e323-3.

2. Heit JA, Kobbervig CE, James AH, Petterson TM, Bailey KR, Melton LJ, 3rd. Trends in the incidence of venous thromboembolism during pregnancy or postpartum: a 30-year population-based study. *Ann of Intern Med*. 2005;143(10):697-706.

3. James AH, Jamison MG, Brancazio LR, Myers ER. Venous thromboembolism during pregnancy and the postpartum period: incidence, risk factors, and mortality. *Am J Obstet Gynecol.* 2006;194(5):1311-5.

4. Lindqvist P, Dahlbäck B, Marŝál K. Thrombotic risk during pregnancy: a population study. *Obstet Gynecol.* 1999;94(4):595-599.

5. Royal College of Obstetricians & Gynaecologists. Thrombosis and Embolism during Pregnancy and the Puerperium, Reducing the Risk (Green-top Guideline No. 37a). 2015 [internet] [https://www.rcog.org.uk/en/guidelines-research-services/guidelines/gtg37a/]. Accessed 4 April 2022.

6. Pomp ER, Lenselink AM, Rosendaal FR, Doggen CJ. Pregnancy, the postpartum period and prothrombotic defects: risk of venous thrombosis in the MEGA study. *J Thromb and Haemost*. 2008;6(4):632–637.

7. Bates SM, Rajasekhar A, Middeldorp S, McLintock C, Rodger MA, James AH, et al. American Society of Hematology 2018 guidelines for management of venous thromboembolism: venous thromboembolism in the context of pregnancy. *Blood Adv.* 2018;2(22):3317-59.

8. Bates SM, Greer IA, Middeldorp S, Veenstra DL, Prabulos A-M, Vandvik PO. VTE, thrombophilia, antithrombotic therapy, and pregnancy: Antithrombotic Therapy and



For peer review only - http://bmjopen.bmj.com/site/about/guidelines.xhtml

Prevention of Thrombosis, 9th ed: American College of Chest Physicians Evidence-Based Clinical Practice Guidelines. *Chest.* 2012;141(2 Suppl):e691S-e736S.

9. Chan W-S, Rey E, Kent NE, et al. Venous thromboembolism and antithrombotic therapy in pregnancy. *J Obstet Gynaecol Can*. 2014;36(6):527-53.

10. D'Alton ME, Friedman AM, Smiley RM, et al. National Partnership for Maternal Safety: Consensus Bundle on Venous Thromboembolism. *Obstet Gynecol*. 2016;128(4):688-698.

11. James A, Birsner M, Kaimal A, in collaboration with American College of Obstetricians and Gynecologists' Committee on Practice Bulletins-Obstetrics. ACOG Practice Bulletin No. 196. Summary: thromboembolism in pregnancy. *Obstet Gynecol*. 2018;132(1):243-248.

12. Lindqvist PG, Hellgren M. Obstetric thromboprophylaxis: the Swedish guidelines. *Adv Hematol*. 2011;2011:157483.

13. McLintock C, Brighton T, Chunilal S, et al. Recommendations for the prevention of pregnancy-associated venous thromboembolism. *Aust N Z J Obstet Gynaecol*. 2012;52(1):3-13.

14. Middleton P, Shepherd E, Gomersall JC. Venous thromboembolism prophylaxis for women at risk during pregnancy and the early postnatal period. Cochrane Database Syst Rev. 2021;3(3):CD001689.

15. Moher D, Liberati A, Tetzlaff J, Altman DG, PRISMA Group, . Preferred reporting items for systematic reviews and meta-analyses: the PRISMA statement. *Ann Intern Med.* 2009;151(4):264-9.

16. Davis S, Goodacre S, Pandor A, et al. Estimating the value of future research to improve guidelines on thromboprophylaxis for women during pregnancy and after delivery.



For peer review only - http://bmjopen.bmj.com/site/about/guidelines.xhtml

2021 [Internet] [https://fundingawards.nihr.ac.uk/award/NIHR131021]. Accessed 4 April 2022.

17. Wilczynski N. McMaster University – HiRU's Approach to Search Filter Development. [Internet] [https://hiru.mcmaster.ca/hiru/HIRU_Hedges_home.aspx]. Accessed 4 April 2022.

18. Moons KGM, Wolff RF, Riley RD, et al. PROBAST: A Tool to Assess Risk of Bias and Applicability of Prediction Model Studies: Explanation and Elaboration. *Ann Intern Med* 2019;170(1):W1-W33.

19. Wolff RF, Moons KGM, Riley RD, et al. PROBAST: A Tool to Assess the Risk of Bias and Applicability of Prediction Model Studies. *Ann Intern Med.* 2019;170(1):51-8.

20. Centre for Reviews and Dissemination. Systematic Reviews: CRD's Guidance for Undertaking Reviews in Health Care: Centre for Reviews and Dissemination, York; 2009.

21. McKenzie JE, Brennan SE, Ryan RE, Thomson HJ, Johnston RV. Chapter 9: Summarizing study characteristics and preparing for synthesis. In: Higgins JPT, Thomas J, Chandler J, Cumpston M, Li T, Page MJ, et al., editors. Cochrane Handbook for Systematic Reviews of Interventions version 6.3 (updated February 2022): Cochrane; 2022.

22. Hosmer DW, Lemeshow S. Applied Logistic Regression (2nd Edition): John Wiley & Sons. New York; 2000.

23. Bauersachs RM, Dudenhausen J, Faridi A, et al. Risk stratification and heparin prophylaxis to prevent venous thromboembolism in pregnant women. *Thromb Haemost*. 2007;98(6):1237-1245.

24. Binstock AB, Larkin JC. Development of a Venous Thromboembolism Prevention Tool in the Postpartum Period. *Obstet Gynecol*. 2019;133(SUPPL 1).

For peer review only

25. Cavazza S, Rainaldi MP, Adduci A, Palareti G. Thromboprophylaxis following cesarean delivery: one site prospective pilot study to evaluate the application of a risk score model. *Thromb Res*. 2012;129(1):28-31.

26. Chau C, Campagna J, Vial M, Rambeaud C, Loundou A, Bretelle F. Use of a personalized iterative score to evaluate risk of venous thromboembolism during pregnancy and puerperium. *Int J Gynaecol Obstet*. 2019;144(3):277-282.

27. Chauleur C, Quenet S, Varlet M-N, et al. Feasibility of an easy-to-use risk score in the prevention of venous thromboembolism and placental vascular complications in pregnant women: a prospective cohort of 2736 women. *Thromb Res*. 2008;122(4):478-484.

28. Dargaud Y, Rugeri L, Fleury C, et al. Personalized thromboprophylaxis using a risk score for the management of pregnancies with high risk of thrombosis: a prospective clinical study. *J Thromb Haemost*. 2017;15(5):897-906.

29. Dargaud Y, Rugeri L, Ninet J, Negrier C, Trzeciak MC. Management of pregnant women with increased risk of venous thrombosis. *Int J Gynaecol Obstet*. 2005;90(3):203-207.

30. Gassmann N, Viviano M, Righini M, Fontana P, Martinez De Tejada B, Blondon M. Estimating the risk thresholds used by guidelines to recommend postpartum thromboprophylaxis. *J Thromb Haemost*. Epub 2020.

31. Hase EA, Barros VIPVLd, Igai AMK, Francisco RPV, Zugaib M. Risk assessment of venous thromboembolism and thromboprophylaxis in pregnant women hospitalized with cancer: Preliminary results from a risk score. *Clinics (Sao Paulo)*. 2018;73:e368.

32. Lindqvist PG, Torsson J, Almqvist A, Bjorgell O. Postpartum thromboembolism: severe events might be preventable using a new risk score model. *Vasc Health Risk Manag*. 2008;4(5):1081-1087.



For peer review only - http://bmjopen.bmj.com/site/about/guidelines.xhtml

33. Lok WY, Kong CW, To WWK. A local risk score model for venous thromboembolism prophylaxis for caesarean section in Chinese women and comparison with international guidelines. *Taiwan J Obstet Gynecol.* 2019;58(4):520-525.

34. Shacaluga A, Rayment R. Venous thromboembolic risk assessment in pregnancy: comparison of the All-Wales maternity risk assessment tool with guidance from the Royal College of Obstetrics and Gynaecology. *Br J Haematol*. 2019;185(1):162-165.

35. Sultan AA, West J, Grainge MJ, et al. Development and validation of risk prediction model for venous thromboembolism in postpartum women: multinational cohort study. *BMJ.* 2016;355:i6253.

36. Testa S, Passamonti SM, Paoletti O, et al. The "Pregnancy Health-care Program" for the prevention of venous thromboembolism in pregnancy. *Intern Emerg Med*. 2015;10(2):129-134.

37. Tran JP, Stribling SS, Ibezim UC, et al. Performance of Risk Assessment Models for Peripartum Thromboprophylaxis. *Reprod Sci*. 2019;26(9):1243-1248.

38. Weiss N, Bernstein PS. Risk factor scoring for predicting venous thromboembolism in obstetric patients. *Am J Obstet Gynecol*. 2000;182(5):1073-5.

39. Ellis-Kahana J, Sparks AD, Gimovsky AC, James AH, Ahmadzia HK. Developing a model for predicting venous thromboembolism in obese pregnant women in a national study. *Thromb Res*. 2020;191:42-49.

40. Collins GS, Reitsma JB, Altman DG, Moons KG. Transparent reporting of a multivariable prediction model for individual prognosis or diagnosis (TRIPOD): the TRIPOD Statement. *BMC Med.* 2015;13:1.

41. Moons KG, de Groot JA, Bouwmeester W, et al. Critical appraisal and data extraction for the systematic reviews of prediction modelling studies: the CHARMS checklist. *PLoS Med.* 2014;11:e1001744.



For peer review only - http://bmjopen.bmj.com/site/about/guidelines.xhtml

42. Andrew L, Ni Ainle F, Blondon M, Rodger MA, Skeith L. Preventing postpartum venous thromboembolism: A call to action to reduce undue maternal morbidity and mortality. *Thromb Res*. 2020;193:190-7.

43. Reps JM, Ryan PB, Rijnbeek PR, Schuemie MJ. Design matters in patient-level prediction: evaluation of a cohort vs. case-control design when developing predictive models in observational healthcare datasets. *J Big Data*. 2021;8(108).

44. Higgins JPT, Thomas J, Chandler J, Cumpston M, Li T, Page MJ, et al. Cochrane Handbook for Systematic Reviews of Interventions version 6.3 (updated February 2022). Cochrane 2022.

45. Knight M, Bunch K, Tuffnell D, Jayakody H, Shakespeare J, Kotnis R, et al. Saving Lives, Improving Mothers' Care - Lessons learned to inform maternity care from the UK and Ireland Confidential Enquiries into Maternal Deaths and Morbidity 2014-16. National Perinatal Epidemiology Unit, University of Oxford, Oxford 2018.

46. National Institute for Health and Care Excellence. Venous thromboembolism in over 16s: reducing the risk of hospital-acquired deep vein thrombosis or pulmonary embolism - NICE Guideline 89. NICE; London UK.; 2019.

47. Knight M. Antenatal pulmonary embolism: Risk factors, management and outcomes. *BJOG*. 2008;115(4):453-461.

48. Lu MY, Blanchard CT, Ausbeck EB, et al. Evaluation of a risk-stratified, heparin-based, obstetric thromboprophylaxis protocol. *Obstet Gynecol*. 2021;138(4):530-8.



For peer review only - http://bmjopen.bmj.com/site/about/guidelines.xhtml

## FIGURE LEGENDS

**Figure 1** **Study flow chart (adapted)**

**Figure 2** **PROBAST assessment summary graph - Review authors' judgements**

For peer review only

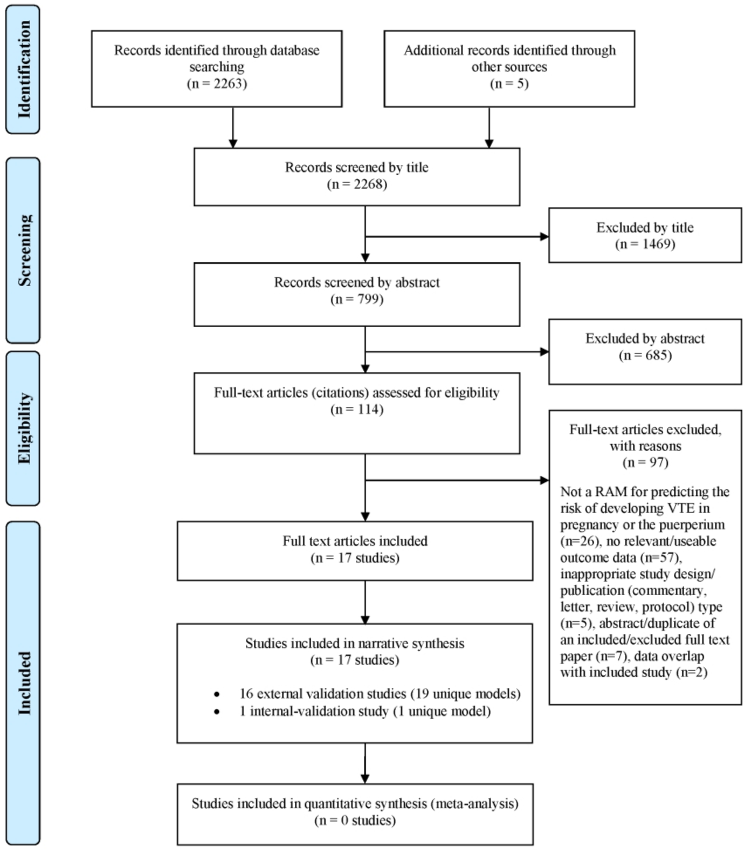


**Figure 1** **Study flow chart (adapted)**

Study flow chart (adapted)

210x297mm (300 x 300 DPI)

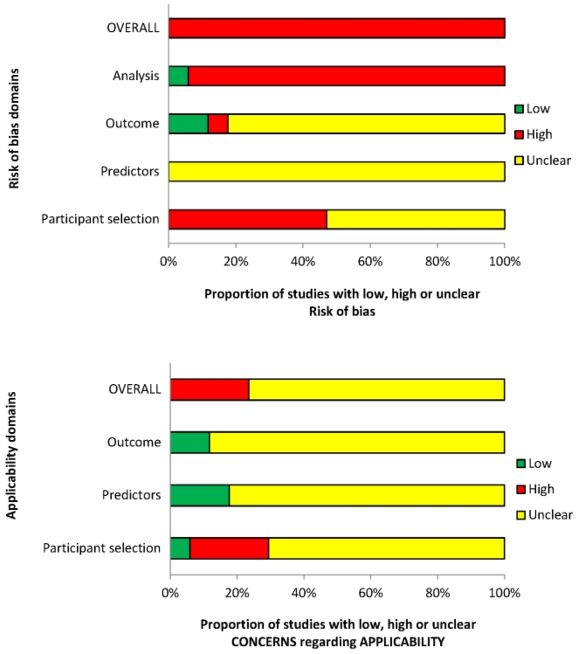


**Figure 2** **PROBAST assessment summary graph - Review authors' judgements**

PROBAST assessment summary graph - Review authors' judgements

210x297mm (300 x 300 DPI)

**APPENDICES**

**Pandor et al. Risk assessment models for venous thromboembolism in pregnancy and in the puerperium – a systematic review**

**Contents**


| | |
|---|---|
| Appendix S1 | Literature search strategies |
| Appendix S2 | List of excluded studies with rationale |
| Appendix S3 | Simplified summary of widely evaluated generic RAMs, their associated characteristics and composite clinical variables |


For peer review only

# APPENDIX S1 LITERATURE SEARCH STRATEGIES

| | |
|---|---|
| **Database searched:** | **Ovid MEDLINE(R) Epub Ahead of Print, In-Process & Other Non-Indexed Citations, Ovid MEDLINE(R) Daily, Ovid MEDLINE and Versions(R)** |
| **Platform or provider used:** | **Ovid SP** |
| **Date of coverage:** | **1946 to February 2021** |
| **Search undertaken:** | **February 2021** |

1 Pregnant Women/ or exp Pregnancy Complications/ or exp Maternal Health Services/ or exp Fetal Monitoring/ or exp Prenatal Diagnosis/ or Perinatal Care/ or Labor pain/ or Analgesia, Obstetric/ or exp Obstetric Surgical Procedures/ or exp Postpartum Period/
2 (pregnan* or antenatal* or ante-natal* or prenatal* or pre-natal* or gestational* or matern* or perinatal* or peri-natal* or postnatal* or post-natal* or postpartum or post-partum or puerper* or obstetric).mp.
3 1 or 2
4 pulmonary embolism/ or thromboembolism/ or venous thromboembolism/ or venous thrombosis/ or upper extremity deep vein thrombosis/
5 (((venous or vein) adj (thrombosis or thromboses or thrombus or thromboemboli*)) or (dvt or vte) or ((pulmonary or lung) adj3 (embolism or emboli or embolus or emboliz* or thromboemboli*))).ti,ab.
6 4 or 5
7 editorial/ or news/ or exp historical article/ or anecdotes as topic/ or comment/ or case report/ or (letter or comment).ti.
8 randomized controlled trial/ or random*.ti,ab.
9 7 not 8
10 animals/ not humans/
11 exp animals, laboratory/
12 exp animal experimentation/
13 exp models, animal/
14 exp rodentia/
15 (rat or rats or mouse or mice).ti.
16 9 or 10 or 11 or 12 or 13 or 14 or 15
17 6 not 16
18 (risk* adj2 assess*).ti,ab.
19 ((score* or scoring) adj2 (tool* or system*)).ti,ab.
20 ((risk* or predict* or prognos*) adj4 (tool* or rule* or index* or indices or score* or scoring or scale* or model* or system* or algorithm* or stratif* or criteria or calculat*)).ti,ab.
21 department of health.ti,ab,au.
22 (guidance or guideline*).ti,hw,pt.
23 18 or 19 or 20 or 21 or 22
24 17 and 23
25 3 and 24

For peer review only

**Databases searched:** **EMBASE**

**Platform or provider used:** **Ovid SP**

**Date of coverage:** **1974 to February 2021**

**Search undertaken:** **February 2021**

1 exp pregnancy/ or maternal health service/ or exp pregnancy complication/ or exp fetus monitoring/ or exp prenatal diagnosis/ or exp perinatal care/ or exp obstetric analgesia/ or exp labor pain/ or exp obstetrics/ or obstetric analgesia/ or exp obstetric operation/ or puerperium/
2 (pregnan* or antenatal* or ante-natal* or prenatal* or pre-natal* or gestational* or matern* or perinatal* or peri-natal* or postnatal* or post-natal* or postpartum or post-partum or puerper* or obstetric or labo?r).mp.
3 1 or 2
4 lung embolism/ or exp venous thromboembolism/ or exp vein thrombosis/ or upper extremity deep vein thrombosis/
5 (((venous or vein) adj (thrombosis or thromboses or thrombus or thromboemboli*)) or (dvt or vte) or ((pulmonary or lung) adj3 (embolism or emboli or embolus or emboliz* or thromboemboli*))).ti,ab.
6 4 or 5
7 editorial/ or comment/ or case report/ or (letter or comment).ti.
8 randomized controlled trial/ or random*.ti,ab.
9 7 not 8
10 exp animal/ not exp human/
11 (rat or rats or mouse or mice).ti.
12 9 or 10
13 6 not 12
14 (risk* adj2 assess*).ti,ab.
15 ((score* or scoring) adj2 (tool* or system*)).ti,ab.
16 ((risk* or predict* or prognos*) adj4 (tool* or rule* or index* or indices or score* or scoring or scale* or model* or system* or algorithm* or stratif* or criteria or calculat*)).ti,ab.
17 department of health.ti,ab,au.
18 (guidance or guideline*).ti,hw,pt.
19 14 or 15 or 16 or 17 or 18
20 13 and 19
21 3 and 20

For peer review only

| | |
|---|---|
| **Databases searched:** | **Cochrane CENTRAL Register of Randomised Controlled Trials & Cochrane Database of Systematic Reviews** |
| **Platform or provider used:** | **www.thecochranelibrary.com** |
| **Date of coverage:** | **Inception to February 2021** |
| **Search undertaken:** | **February 2021** |

#1 MeSH descriptor: [Pregnancy] explode all trees
#2 MeSH descriptor: [Pregnancy Complications] 1 tree(s) exploded
#3 MeSH descriptor: [Maternal Health Services] explode all trees
#4 MeSH descriptor: [Fetal Monitoring] explode all trees
#5 MeSH descriptor: [Perinatal Care] explode all trees
#6 MeSH descriptor: [Labor Pain] explode all trees
#7 MeSH descriptor: [Analgesia, Obstetrical] explode all trees
#8 MeSH descriptor: [Obstetric Surgical Procedures] explode all trees
#9 MeSH descriptor: [Postpartum Period] explode all trees
#10 (pregnan* or antenatal* or "ante-natal*" or prenatal* or "pre-natal*" or gestational* or matern* or perinatal* or "peri-natal*" or postnatal* or "post-natal*" or postpartum or "post-partum" or puerper* or obstetric):ti,ab,kw (Word variations have been searched)
#11 MeSH descriptor: [Pulmonary Embolism] explode all trees
#12 MeSH descriptor: [Venous Thromboembolism] explode all trees
#13 MeSH descriptor: [Venous Thrombosis] explode all trees
#14 MeSH descriptor: [Upper Extremity Deep Vein Thrombosis] explode all trees
#15 ((venous or vein) near/2 (thrombosis or thromboses or thrombus or thromboemboli*)):ti,ab,kw OR ((dvt or vte)):ti,ab,kw OR ((pulmonary or lung) near/2 (embolism or emboli or embolus or emboliz* or thromboemboli*)):ti,ab,kw (Word variations have been searched)
#16 #1 or #2 or #3 or #4 or #5 or #6 or #7 or #8 or #9 or #10
#17 #11 or #12 or #13 or #14 or #15
#18 #16 and #17
#19 (risk* or predict* or prognos*):ti,ab,kw AND (tool* or rule* or index* or indices or score* or scoring or scale* or model* or system* or algorithm* or stratif* or criteria or calculat*):ti,ab,kw OR ((pulmonary or lung) near/3 (embolism or emboli or embolus or emboliz* or thromboemboli*)):ti,ab,kw (Word variations have been searched)
#20 (score* or scoring) near/2 (tool* or system*)
#21 guidance or guideline* or "department of health"
#22 #19 or #20 or #21
#23 #18 and #22

For peer review only

**APPENDIX S2 LIST OF EXCLUDED STUDIES WITH RATIONALE**

| | Authors, year | Reason for exclusion |
|---|---|---|
| 1. | Abdul Sultan et al., 2013 [1] | Not a RAM for predicting the risk of developing VTE in pregnancy or the puerperium |
| 2. | Ahmadzia et al., 2019 [2] | Abstract of included full text study (Ellis-Kahana 2020) |
| 3. | Alsayegh et al., 2016 [3] | No relevant/useable outcome data |
| 4. | Bahl et al., 2010 [4] | Not a RAM for predicting the risk of developing VTE in pregnancy or the puerperium |
| 5. | Banfield et al., 2013 [5] | No relevant/useable outcome data |
| 6. | Bare et al., 2013 [6] | Not a RAM for predicting the risk of developing VTE in pregnancy or the puerperium |
| 7. | Barros et al., 2017 [7] | No relevant/useable outcome data |
| 8. | Barros et al., 2017 [8] | No relevant/useable outcome data |
| 9. | Barros et al., 2020 [9] | No relevant/useable outcome data |
| 10. | Barros et al., 2011 [10] | No relevant/useable outcome data |
| 11. | Bastek et al., 2011 [11] | Not a RAM for predicting the risk of developing VTE in pregnancy or the puerperium |
| 12. | Beckett et al., 2013 [12] | No relevant/useable outcome data |
| 13. | Berkin et al., 2016 [13] | No relevant/useable outcome data |
| 14. | Blondon et al., 2015 [14] | Not a RAM for predicting the risk of developing VTE in pregnancy or the puerperium |
| 15. | Blondon and Hugon-Rodin 2017 [15] | Commentary |
| 16. | Campbell 2013 [16] | No relevant/useable outcome data |
| 17. | Cavazza et al., 2010 [17] | Abstract of included full text study (Cavazza 2012) |
| 18. | Chauleur et al., 2017 [18] | Data overlap - patients included in Chauleur 2018 (included study) |
| 19. | Chauleur et al., 2010 [19] | No relevant/useable outcome data |
| 20. | Chauleur et al., 2018 [20] | No relevant/useable outcome data |
| 21. | Cooley et al., 2016 [21] | No relevant/useable outcome data |
| 22. | Creagh et al., 2014 [22] | Not a RAM for predicting the risk of developing VTE in pregnancy or the puerperium |
| 23. | Creagh et al., 2013 [23] | Not a RAM for predicting the risk of developing VTE in pregnancy or the puerperium |
| 24. | Crowley et al., 2013 [24] | Abstract of excluded full text study (Crowley 2017) |
| 25. | Crowley et al., 2017 [25] | No relevant/useable outcome data |
| 26. | Crowley et al., 2013 [26] | Abstract of excluded full text study (Crowley 2017) |
| 27. | Cunningham et al., 2015 [27] | Not a RAM for predicting the risk of developing VTE in pregnancy or the puerperium |

For peer review only

| | | |
|---|---|---|
| 28. | Cutts et al., 2014 [28] | Not a RAM for predicting the risk of developing VTE in pregnancy or the puerperium |
| 29. | Cutts et al., 2011 [29] | Not a RAM for predicting the risk of developing VTE in pregnancy or the puerperium |
| 30. | Dargaud et al., 2015 [30] | Abstract of included full text study (Dargaud 2017) |
| 31. | Dargaud et al., 2009 [31] | Data overlap - patients included in Dargaud 2017 (included study) |
| 32. | Dargaud et al., 2009 [32] | No relevant/useable outcome data |
| 33. | Davis and Hadpawat-Lee 2017 [33] | No relevant/useable outcome data |
| 34. | Dentali et al., 2020 [34] | Not a RAM for predicting the risk of developing VTE in pregnancy or the puerperium |
| 35. | Francis Kim et al., 2020 [35] | No relevant/useable outcome data |
| 36. | Francis Kim et al., 2020 [36] | No relevant/useable outcome data |
| 37. | Fuller et al., 2018 [37] | Not a RAM for predicting the risk of developing VTE in pregnancy or the puerperium |
| 38. | Gassmann et al., 2020 [38] | Abstract of included full text study (Gassmann 2020) |
| 39. | Gerhardt et al., 2016 [39] | Not a RAM for predicting the risk of developing VTE in pregnancy or the puerperium |
| 40. | Gherghe et al., 2012 [40] | No relevant/useable outcome data |
| 41. | Goffman et al., 2009 [41] | No relevant/useable outcome data |
| 42. | Gomez et al., 2020 [42] | No relevant/useable outcome data |
| 43. | Grille et al., 2015 [43] | No relevant/useable outcome data |
| 44. | Goodfellow et al., 2017 [44] | No relevant/useable outcome data |
| 45. | Grant et al., 2016 [45] | No relevant/useable outcome data |
| 46. | Handa et al., 2015 [46] | No relevant/useable outcome data |
| 47. | Harris et al., 2016 [47] | No relevant/useable outcome data |
| 48. | Hayes-Ryan and Byrne 2011 [48] | No relevant/useable outcome data |
| 49. | Hayes-Ryan and Byrne 2012 [49] | No relevant/useable outcome data |
| 50. | Heath and Goodfellow 2016 [50] | No relevant/useable outcome data |
| 51. | Henke and Pannucci 2010 [51] | Review |
| 52. | Kazi et al., 2020 [52] | Not a RAM for predicting the risk of developing VTE in pregnancy or the puerperium |
| 53. | Lacoss and Jheeta 2017 [53] | Not a RAM for predicting the risk of developing VTE in pregnancy or the puerperium |
| 54. | Li et al., 2018 [54] | Not a RAM for predicting the risk of developing VTE in pregnancy or the puerperium |
| 55. | Lindqvist 2018 [55] | Letter |
| 56. | Lindqvist and Hellgren 2011 [56] | No relevant/useable outcome data |
| 57. | Lindqvist et al., 2002 [57] | No relevant/useable outcome data |

For peer review only

| | | |
|---|---|---|
| 58. | Lou Mercade et al., 2017 [58] | No relevant/useable outcome data |
| 59. | Marks and Maiti 2018 [59] | No relevant/useable outcome data |
| 60. | Mcarthur et al., 2011 [60] | No relevant/useable outcome data |
| 61. | Mpouzouki et al., 2013 [61] | No relevant useable outcome data |
| 62. | Naidoo et al., 2019 [62] | No relevant/useable outcome data |
| 63. | Nct 2018 [63] | Protocol |
| 64. | Noone et al., 2013 [64] | No relevant/useable outcome data |
| 65. | O'Connor et al., 2011 [65] | Not a RAM for predicting the risk of developing VTE in pregnancy or the puerperium |
| 66. | O'Keefe et al., 2019 [66] | Not a RAM for predicting the risk of developing VTE in pregnancy or the puerperium |
| 67. | Omunakwe et al., 2017 [67] | No relevant/useable outcome data |
| 68. | Orfanelli et al., 2017 [68] | No relevant/useable outcome data |
| 69. | O'Shaughnessy et al., 2017 [69] | No relevant/useable outcome data |
| 70. | O'Shaughnessy et al., 2018 [70] | No relevant/useable outcome data |
| 71. | O'Shaughnessy et al., 2019 [71] | No relevant/useable outcome data |
| 72. | O'Sullivan et al., 2020 [72] | No relevant/useable outcome data |
| 73. | O'Sullivan et al., 2009 [73] | Not a RAM for predicting the risk of developing VTE in pregnancy or the puerperium |
| 74. | Ottawa Hospital Research Institute and Leo Pharma 2002 [74] | Protocol |
| 75. | Palmerola et al., 2016 [75] | No relevant/useable outcome data |
| 76. | Pannucci and Fleming 2017 [76] | Not a RAM for predicting the risk of developing VTE in pregnancy or the puerperium |
| 77. | Pierce-Williams et al., 2018 [77] | No relevant/useable outcome data |
| 78. | Potdar et al., 2006 [78] | No relevant/useable outcome data |
| 79. | Rahim et al., 2020 [79] | Not a RAM for predicting the risk of developing VTE in pregnancy or the puerperium |
| 80. | Righini et al., 2013 [80] | Not a RAM for predicting the risk of developing VTE in pregnancy or the puerperium |
| 81. | Righini and Le Gal 2013 [81] | Not a RAM for predicting the risk of developing VTE in pregnancy or the puerperium |
| 82. | Ryan 2019 [82] | No relevant/useable outcome data |
| 83. | Saad et al., 2018 [83] | Abstract of included full text study (Tran 2019) |
| 84. | Santos et al., 2015 [84] | No relevant/useable outcome data |
| 85. | Schoenbeck et al., 2011 [85] | No relevant/useable outcome data |
| 86. | Sellappan et al., 2012 [86] | Not a RAM for predicting the risk of developing VTE in pregnancy or the puerperium |
| 87. | Shacaluga et al., 2017 [87] | No relevant/useable outcome data |

For peer review only

| | | |
|---|---|---|
| 88. | Tan and Wisdom 2006 [88] | No relevant/useable outcome data |
| 89. | Tang and Marsden 2011 [89] | No relevant/useable outcome data |
| 90. | Taylor et al., 2000 [90] | No relevant/useable outcome data |
| 91. | Testa et al., 2010 [91] | No relevant/useable outcome data |
| 92. | Testa et al., 2013 [92] | No relevant/useable outcome data |
| 93. | Touhami et al., 2018 [93] | Not a RAM for predicting the risk of developing VTE in pregnancy or the puerperium |
| 94. | Usoro et al., 2019 [94] | Not a RAM for predicting the risk of developing VTE in pregnancy or the puerperium |
| 95. | Valdre et al., 2016 [95] | No relevant/useable outcome data |
| 96. | Von Hawrylak 2018 [96] | No relevant/useable outcome data |
| 97. | Zhang et al., 2020 [97] | No relevant/useable outcome data |

For peer review only

**REFERENCES (APPENDIX S2)**


1. Abdul Sultan A, West J, Tata LJ, et al. Risk of first venous thromboembolism in pregnant women in hospital: population based cohort study from England. *BMJ (Clinical Research Ed)* 2013;347:f6099. doi: https://dx.doi.org/10.1136/bmj.f6099
2. Ahmadzia HK, Ellis-Kahana JK, Sparks AD, et al. 859: Predicting venous thromboembolism in obese pregnant women in a national study. *American Journal of Obstetrics and Gynecology* 2019;220(1 Supplement):S559-S60. doi: http://dx.doi.org/10.1016/j.ajog.2018.11.882
3. Alsayegh F, Al-Jassar W, Wani S, et al. Venous Thromboembolism Risk and Adequacy of Prophylaxis in High Risk Pregnancy in the Arabian Gulf. *Current Vascular Pharmacology* 2016;14(4):368-73.
4. Bahl V, Hu HM, Henke PK, et al. A validation study of a retrospective venous thromboembolism risk scoring method. *Annals of Surgery* 2010;251(2):344-50. doi: https://dx.doi.org/10.1097/SLA.0b013e3181b7fca6
5. Banfield DA, Page LM, Cotzias CS, et al. Postnatal risk assessment of venous thromboembolism (VTE). *Archives of Disease in Childhood: Fetal and Neonatal Edition* 2013;98(SUPPL. 1) doi: http://dx.doi.org/10.1136/archdischild-2013-303966.121
6. Bare LA, De Haan HG, Arellano AR, et al. A simple genetic thrombosis score of five single nucleotide polymorphisms is associated with risk of first venous thrombosis in pregnant women. *Blood* 2013;122(21)
7. Barros V, Hase E, Igai AM, et al. Cancer and venous thromboembolism in pregnancy. *Research and Practice in Thrombosis and Haemostasis* 2017;1(Supplement 1):54. doi: http://dx.doi.org/10.1002/rth2.12012
8. Barros V, Igai AM, Bortolotto MR, et al. Obesity and thromboembolic risk during hospitalization in pregnancy: Preliminary results. *Research and Practice in Thrombosis and Haemostasis* 2017;1(Supplement 1):1030.
9. Barros V, Igai A, Fernanda B, et al. Preventing maternal death and morbidity from venous thromboembolism (VTE): Results from a VTE risk score trial during hospitalization. *Research and Practice in Thrombosis and Haemostasis* 2020;4(SUPPL 1):1197-98. doi: http://dx.doi.org/10.1002/rth2.12393
10. Barros V, Oliveira A, Spadotto F, et al. Thrombosis risk score in pregnancy: Preliminary results from a model. *Journal of Perinatal Medicine* 2011;39(SUPPL. 1) doi: http://dx.doi.org/10.1515/jpm-2012-1008
11. Bastek JA, Dainty E, Srinivas SK, et al. Pulmonary embolism in pregnancy: Do the diagnostic algorithms from non-pregnant populations apply? *American Journal of Obstetrics and Gynecology* 2011;204(1 SUPPL.):S317. doi: http://dx.doi.org/10.1016/j.ajog.2010.10.834
12. Beckett V, Graham I, Keriakos R. Audit of prescribing and administration of thromboprophylaxis medication and stockings to in-patients of the obstetric and gynaecological wards at the

Jessops Wing, Sheffield teaching hospitals. *BJOG: An International Journal of Obstetrics and Gynaecology* 2013;120(SUPPL. 1):437-38. doi: http://dx.doi.org/10.1111/1471-0528.12301

13. Berkin JA, Lee C, Landsberger E, et al. Scorecard implementation improves identification of postpartum patients at risk for venous thromboembolism. *Journal of Healthcare Risk Management: The Journal of the American Society for Healthcare Risk Management* 2016;36(1):8-13. doi: https://dx.doi.org/10.1002/jhrm.21229
14. Blondon M, Harrington L, Boehlen F, et al. Pre-pregnancy BMI and delivery BMI as risk factors for postpartum VTE: A population-based, case-control study. *Journal of Thrombosis and Haemostasis* 2015;13(SUPPL. 2):106. doi: http://dx.doi.org/10.1111/jth.12993
15. Blondon M, Hugon-Rodin J. A clinical risk score to predict the incidence of postpartum venous thromboembolism. *Evidence-Based Medicine* 2017;22(3):98. doi: http://dx.doi.org/10.1136/ebmed-2017-110680
16. Campbell MJ. Full audit cycle assessing how current antenatal inpatients are risk assessed for venous thromboembolic (VTE) disease as an inpatient and antenatally at Ninewells Hospital, Dundee, Scotland, October 2012. *BJOG: An International Journal of Obstetrics and Gynaecology* 2013;120(SUPPL. 1):472. doi: http://dx.doi.org/10.1111/1471-0528.12301
17. Cavazza S, Rainaldi MP, Bonazzi F, et al. Thomboprophylaxis following cesarean section: A one site prospective pilot study to evaluate the application of a risk score model. *Pathophysiology of Haemostasis and Thrombosis* 2010;37(SUPPL. 1):A4. doi: http://dx.doi.org/10.1159/000318095
18. Chauleur C, Gris JC, Laporte S, et al. Implementation of risk score-guided prophylaxis in over 2000 pregnant women at risk of thrombotic events: Impact on morbidity. A quasi-experimental prospective study. *Thrombosis Research* 2017;151(Supplement 1):S107-S08.
19. Chauleur C, Gris JC, Laporte S, et al. Use of the Delphi method to facilitate antithrombotics prescription during pregnancy. *Thrombosis Research* 2010;126(2):88-92. doi: https://dx.doi.org/10.1016/j.thromres.2010.01.012
20. Chauleur C, Gris J-C, Laporte S, et al. Benefit of Risk Score-Guided Prophylaxis in Pregnant Women at Risk of Thrombotic Events: A Controlled Before-and-After Implementation Study. *Thrombosis and Haemostasis* 2018;118(9):1564-71. doi: https://dx.doi.org/10.1055/s-0038-1668524
21. Cooley SM, Donnelly JC, Deering M, et al. Thrombocalc: Personalized postpartum VTE risk assessment in a high-throughput environment. *American Journal of Obstetrics and Gynecology* 2016;214(1 SUPPL. 1):S26-S27.
22. Creagh MD, Davies B, Webborn A, et al. Practice using a programme for avoidance of venous thrombo-embolism (VTE) in pregnancy; Is post delivery risk assessment undertaken and

For peer review only

effective? *British Journal of Haematology* 2014;165(SUPPL. 1):44-45. doi: http://dx.doi.org/10.1111/bjh.12802

23. Creagh MD, Dehnel A, Rider L, et al. Does systematic risk assessment in pregnancy identify women at risk for venous thromboembolism and so avoid thrombosis? Experience of an 18 month programme based on national guidance. *Journal of Thrombosis and Haemostasis* 2013;11(SUPPL. 2):867.
24. Crowley MP, Noone C, Higgins JR, et al. Venous thromboembolism (VTE) prophylaxis in hospitalized obstetric patients in Ireland: A multicentre cross-sectional study. *Journal of Thrombosis and Haemostasis* 2013;11(SUPPL. 2):739.
25. Crowley MP, Noone C, Higgins JR, et al. A Multicentre Study of Thromboprophylaxis in Pregnancy. *Irish Medical Journal* 2017;110(5):567.
26. Crowley MP, Noone C, Higgins JR, et al. Venous thromboembolism (VTE) prophylaxis in hospitalized obstetric patients: A multicentre cross-sectional study. *Haematologica* 2013;98(SUPPL. 1):438.
27. Cunningham Y, MacDonald H, Coutts S. Bettering antenatal and postnatal risk assessment for venous thromboembolism - A quality improvement approach. *BJOG: An International Journal of Obstetrics and Gynaecology* 2015;122(SUPPL. 2):263. doi: http://dx.doi.org/10.1111/14710528.13383
28. Cutts BA, Tran HA, Merriman E, et al. The utility of the Wells clinical prediction model and ventilation-perfusion scanning for pulmonary embolism diagnosis in pregnancy. *Blood Coagulation & Fibrinolysis: An International Journal in Haemostasis and Thrombosis* 2014;25(4):375-8. doi: https://dx.doi.org/10.1097/MBC.0000000000000054
29. Cutts BA, Tran H, Merriman E, et al. The utility of the wells clinical prediction model and ventilation-perfusion scanning for pulmonary embolism in pregnancy. *Journal of Thrombosis and Haemostasis* 2011;9(SUPPL. 2):631. doi: http://dx.doi.org/10.1111/j.1538-7836.2011.04380_3.x
30. Dargaud YG, Rugeri L, Fleury C, et al. Individually tailored prophylaxis using a risk score for the management of pregnant women with increased risk of venous thromboembolism. *Blood* 2015;126(23):889.
31. Dargaud Y, Rugeri L, Vergnes MC, et al. A risk score for the management of pregnant women with increased risk of venous thromboembolism: a multicentre prospective study. *British Journal of Haematology* 2009;145(6):825-35. doi: https://dx.doi.org/10.1111/j.1365-2141.2009.07698.x
32. Dargaud Y, Rugeri L, Vergnes MC, et al. Management of pregnancies with high risk of thrombosis: A multicenter prospective study. *Journal of Thrombosis and Haemostasis* 2009;7(S2):1060-61. doi: http://dx.doi.org/10.1111/j.1538-7836.2009.03473-2.x

For peer review only

33. Davis B, Hadpawat-Lee A. Venous thromboembolism: Comparing risk assessment tools for postpartum prophylaxis in a teaching hospital. *Obstetrics and Gynecology* 2017;129(Supplement 1):114S.

34. Dentali F, Fontanella A, Cohen AT, et al. Derivation and Validation of a Prediction Model for Venous Thromboembolism in Primary Care. *Thrombosis and Haemostasis* 2020;120(4):692-701. doi: http://dx.doi.org/10.1055/s-0040-1701483

35. Francis Kim AP, Saleh M, Melendez Torres A, et al. Age and body mass index can screen for VTE risk at labor and delivery admission. *Obstetrics and Gynecology* 2020;135(Supplement 1):39S.

36. Francis Kim AP, Saleh M, Torres AM, et al. Impact of delivery-related factors on venous thromboembolism risk during labor. *Obstetrics and Gynecology* 2020;135(Supplement 1):128S.

37. Fuller GW, Nelson-Piercy C, Hunt BJ, et al. Consensus-derived clinical decision rules to guide advanced imaging decisions for pulmonary embolism in pregnancy and the postpartum period. *European Journal of Emergency Medicine: Official Journal of the European Society for Emergency Medicine* 2018;25(3):221-22. doi: https://dx.doi.org/10.1097/MEJ.0000000000000477

38. Gassmann N, Viviano M, Fontana P, et al. Comparison of recommended postpartum thromboprophylaxis and of absolute risk thresholds according to the RCOG, ACCP and ACOG guidelines. *Research and Practice in Thrombosis and Haemostasis* 2020;4(SUPPL 1):32-33. doi: http://dx.doi.org/10.1002/rth2.12401

39. Gerhardt A, Scharf RE, Greer IA, et al. Hereditary risk factors for thrombophilia and probability of venous thromboembolism during pregnancy and the puerperium. *Blood* 2016;128(19):2343-49. doi: http://dx.doi.org/10.1182/blood-2016-03-703728

40. Gherghe M, Moss H, Gibson J, et al. Risk assessment to reduce thromboembolic disease in obstetric practice: A regional audit. *Archives of Disease in Childhood: Fetal and Neonatal Edition* 2012;97(SUPPL. 1):A49. doi: http://dx.doi.org/10.1136/fetalneonatal-2012-301809.156

41. Goffman D, Fisher N, Kowenski J, et al. Utilization of a checklist to evaluate risk for postpartum venous thromboembolism. *American Journal of Obstetrics and Gynecology* 2009;201(6 SUPPL. 1):S297. doi: http://dx.doi.org/10.1016/j.ajog.2009.10.848

42. Gomez D, Orfanelli T, Awomolo A, et al. A comparison of pregnancy-specific risk scoring systems for venous thromboembolic pharmacoprophylaxis in hospitalized maternity patients. *The Journal of Maternal-Fetal & Neonatal Medicine: The Official Journal of the European Association of Perinatal Medicine, the Federation of Asia and Oceania Perinatal Societies, the International Society of Perinatal Obstetricians* 2020:1-8. doi: https://dx.doi.org/10.1080/14767058.2020.1832072

For peer review only

43. Grille S, Castro V, Turcatti P, et al. Prophylaxis for venous thromboembolic disease in pregnancy and postpartum period. *Haematologica* 2015;100(SUPPL. 1):626.

44. Goodfellow A, Heath S, George L, et al. Perinatal venous thromboembolism state based prevention strategy: Midwifery risk assessment tool. *Thrombosis Research* 2017;151(Supplement 1):S132.

45. Grant GH, Merriman JB, Hoffman MK. Implementation and efficacy of a formalized venous thromboembolism prevention strategy in the peripartum population. *American Journal of Obstetrics and Gynecology* 2016;214(1 SUPPL. 1):S227-S28.

46. Handa S, Singh S, Bhandal N. Retrospective audit of venous thromboembolism risk assessment in obstetric postoperative patients. *Anaesthesia* 2015;70(SUPPL. 3):72. doi: http://dx.doi.org/10.1111/anae.13136

47. Harris C, Sulmers C, Groesch K, et al. Venous thromboembolism: Padua prediction score in the obstetric patient. *Obstetrics and Gynecology* 2016;127(Supplement 1):88S. doi: http://dx.doi.org/10.1097/01.AOG.0000483795.00678.28

48. Hayes-Ryan D, Byrne B. Thromboprophylaxis in pregnancy the practical implications of guidelines. *American Journal of Obstetrics and Gynecology* 2011;204(1 SUPPL.):S315-S16. doi: http://dx.doi.org/10.1016/j.ajog.2010.10.829

49. Hayes-Ryan D, Byrne BM. Prevention of thrombosis in pregnancy: how practical are consensus derived clinical practice guidelines? *Journal of Obstetrics and Gynaecology: The Journal of the Institute of Obstetrics and Gynaecology* 2012;32(8):740-2. doi: https://dx.doi.org/10.3109/01443615.2012.693982

50. Heath S, Goodfellow A. Maternal venous thromboembolism (VTE) risk assessment. *Journal of Paediatrics and Child Health* 2016;52(Supplement 2):69. doi: http://dx.doi.org/10.1111/jpc.13194

51. Henke PK, Pannucci CJ. Venous thromboembolism risk factor assessment and prophylaxis. *Phlebology* 2010;25(5):219-23. doi: http://dx.doi.org/10.1258/phleb.2010.010018

52. Kazi S, McLeod A, Berndl A. Approach to venous thromboembolism risk in women with physical disability in pregnancy-multidisciplinary survey. *Research and Practice in Thrombosis and Haemostasis* 2020;4(SUPPL 1):1283-84. doi: http://dx.doi.org/10.1002/rth2.12393

53. LaCoss E, Jheeta S. How accurate is information documented on inpatient electronic venous thromboembolism risk-assessment forms? *Journal of Pharmacy and Pharmacology* 2017;69(Supplement 1):13-14. doi: http://dx.doi.org/10.1111/jphp.12850

54. Li C, Zuo Y, Karp D, et al. Identifying clinical and epidemiological risk factors associated with thrombosis and pregnancy morbidity in a large cohort of Chinese APS patients. *International Journal of Rheumatic Diseases* 2018;21(Supplement 1):126-27. doi: http://dx.doi.org/10.1111/1756-185X.13361

For peer review only

55. Lindqvist PG. Re: Postpartum venous thromboembolism prophylaxis may cause more harm than benefit: a critical analysis of international guidelines through an evidence-based lens: Postpartum thromboprophylaxis is cost-effective using the Swedish thromboprophylaxis algorithm. *BJOG: An International Journal of Obstetrics and Gynaecology* 2018;125(9):1194-95. doi: http://dx.doi.org/10.1111/1471-0528.15266

56. Lindqvist PG, Hellgren M. Obstetric thromboprophylaxis: the Swedish guidelines. *Advances in hematology* 2011;2011:157483. doi: https://dx.doi.org/10.1155/2011/157483

57. Lindqvist PG, Kublikas M, Dahlback B. Individual risk assessment of thrombosis in pregnancy. *Acta Obstetricia et Gynecologica Scandinavica* 2002;81(5):412-6.

58. Lou Mercade AC, Saviron Cornudella R, Cornudella Lacasa R. AnticoagObs: An application for antenatal and postpartum risk assessment and prophylaxis of venous thromboembolism. *Thrombosis Research* 2017;151(Supplement 1):S127.

59. Marks D, Maiti S. Venous thromboembolism risk assessment and prescribing on the postnatal ward at north manchester general hospital: A clinical audit. *BJOG: An International Journal of Obstetrics and Gynaecology* 2018;125(Supplement 3):25. doi: http://dx.doi.org/10.1111/1471-0528.15493

60. McArthur L, Whittaker B, Rajasri A, et al. Venous thrombo-embolic risk in pregnancy and the puerperium: Can self-assessment identify risk and guide further care? *British Journal of Haematology* 2011;153(SUPPL. 1):84-85. doi: http://dx.doi.org/10.1111/j.13652141.2011.08609

61. Mpouzouki A, Bowles L, Beski S, et al. A retrospective review of risk factors for thrombosis in women presenting with venous thromboembolism (VTE) during pregnancy and the puerperium. *Thrombosis Research* 2013;131(SUPPL. 1):S82. doi: http://dx.doi.org/10.1016/S0049-3848%2813%2970070-7

62. Naidoo P, Mothilal R, Snyman LC. Assessment and management of venous thrombo-embolism risk during pregnancy and the puerperium (SAVE): The South African cohort. *South African Medical Journal* 2019;109(3):186-92. doi: https://dx.doi.org/10.7196/SAMJ.2019.v109i3.13487

63. NCT. Pregnancy and Risk of Venous Thromboembolism. *https://clinicaltrialsgov/show/NCT03659708* 2018

64. Noone CM, O'Shea S, Crowley MP, et al. Venous thromboembolism (VTE) risk assessment & prophylaxis in irish hospitalized obstetric patients: A nationwide cross-sectional study. *Blood* 2013;122(21)

65. O'Connor C, Moriarty J, Walsh J, et al. The application of a clinical risk stratification score may reduce unnecessary investigations for pulmonary embolism in pregnancy. *The Journal of Maternal-Fetal & Neonatal Medicine: The Official Journal of the European Association of Perinatal Medicine, the Federation of Asia and Oceania Perinatal Societies, the International*

For peer review only

*Society of Perinatal Obstetricians* 2011;24(12):1461-4. doi: https://dx.doi.org/10.3109/14767058.2011.614652

66. O'Keefe D, Hui L, Ho P, et al. Evaluation of global coagulation assays for assessment of clotting function and risk of venous thromboembolism in pregnancy and post-partum. *Australian and New Zealand Journal of Obstetrics and Gynaecology* 2019;59(Supplement 1):68-69. doi: http://dx.doi.org/10.1111/ajo.13067
67. Omunakwe HE, Roberts LN, Patel JP, et al. Re: A comparison of the recommendations for pharmacologic thromboembolism prophylaxis after caesarean delivery from the major guidelines: Impact on thromboprophylaxis rates of implementing Royal College of Obstetricians and Gynaecologists' guidance for reducing the risk of ante- and postnatal venous thromboembolism. *BJOG: An International Journal of Obstetrics and Gynaecology* 2017;124(5):831-32. doi: http://dx.doi.org/10.1111/1471-0528.14001
68. Orfanelli T, Awomolo A, Gomez D, et al. A comparison of peripartum scoring systems of anticoagulation for venous thromboembolic prophylaxis. *Obstetrics and Gynecology* 2017;129(Supplement 1):112S.
69. O'Shaughnessy F, Donnelly JC, Cooley SM, et al. Thrombocalc: implementation and uptake of personalized postpartum venous thromboembolism risk assessment in a high-throughput obstetric environment. *Acta obstetricia et gynecologica Scandinavica* 2017;96(11):1382-90. doi: https://dx.doi.org/10.1111/aogs.13206
70. O'Shaughnessy F, Donnelly J, Cooley S, et al. Initiating postpartum pharmacological thromboprophylaxis: A comparison of international recommendations. *American Journal of Obstetrics and Gynecology* 2018;218(1 Supplement 1):S493-S94.
71. O'Shaughnessy F, Ni Ainle F, Jennifer D, et al. Preventing postpartum venous thromboembolism; impact of systematic VTE risk assessment. *Research and Practice in Thrombosis and Haemostasis* 2019;3(Supplement 1):225-26. doi: http://dx.doi.org/10.1002/rth2.12227
72. O'Sullivan C, Christensen K, Gallagher G, et al. Peripartum vte risk assessment in a tertiary centre: An audit. *Journal of Paediatrics and Child Health* 2020;56(SUPPL 1):143. doi: http://dx.doi.org/10.1111/jpc.14868
73. O'Sullivan C, Moriarty J, Walsh J, et al. Application of a clinical risk stratification score in pregnancy and the puerperium - Can unnecessary investigations for pulmonary embolism be avoided? *American Journal of Obstetrics and Gynecology* 2009;201(6 SUPPL. 1):S68. doi: http://dx.doi.org/10.1016/j.ajog.2009.10.161
74. Ottawa Hospital Research Institute, LEO Pharma. The STOP CLOT Pilot Study: Study of Low Molecular Weight Heparin in High Risk Cesarean Section: https://ClinicalTrials.gov/show/NCT00225108, 2002.
75. Palmerola KL, D'Alton ME, Brock CO, et al. A comparison of recommendations for pharmacologic thromboembolism prophylaxis after caesarean delivery from three major

For peer review only

guidelines. *BJOG: An International Journal of Obstetrics and Gynaecology* 2016;123(13):2157-62. doi: https://dx.doi.org/10.1111/1471-0528.13706

76. Pannucci C, Fleming K. The electronic medical record underestimates venous thromboembolism risk using the 2005 caprini score compared with face-to-face interaction. *Journal of Vascular Surgery: Venous and Lymphatic Disorders* 2017;5(1):152-53. doi: http://dx.doi.org/10.1016/j.jvsv.2016.10.028
77. Pierce-Williams R, Cohen I, D'Adamo C, et al. Venous thromboembolism prophylaxis after cesarean section: A quality improvement project. *Obstetrics and Gynecology* 2018;131(Supplement 1):104S-05S.
78. Potdar N, Jabbar B, Burrell SJ. Thromboprophylaxis after vaginal delivery: a district general hospital experience. *Journal of Obstetrics and Gynaecology: The Journal of the Institute of Obstetrics and Gynaecology* 2006;26(1):24-6.
79. Rahim MN, Williamson C, Kametas NA, et al. Using pregnancy to assess risk and predict women's health. *EClinicalMedicine* 2020;20:100292. doi: http://dx.doi.org/10.1016/j.eclinm.2020.100292
80. Righini M, Jobic C, Boehlen F, et al. Predicting deep venous thrombosis in pregnancy: external validation of the LEFT clinical prediction rule. *Haematologica* 2013;98(4):545-8. doi: https://dx.doi.org/10.3324/haematol.2012.072009
81. Righini M, Le Gal G. Predicting deep venous thrombosis in pregnancy: External validation of the 'left' clinical prediction rule. *Journal of Thrombosis and Haemostasis* 2013;11(SUPPL. 3):91. doi: http://dx.doi.org/10.1111/jth.12443
82. Ryan N. Maternal thromboprophylaxis assessment tool: An audit. *Irish Journal of Medical Science* 2019;188(Supplement 7):S53. doi: http://dx.doi.org/10.1007/s11845-019-02053-0
83. Saad A, Tran JP, Stribling S, et al. Performance of risk assessment models for thromboprophylaxis in an obstetric population. *American Journal of Obstetrics and Gynecology* 2018;218(1 Supplement 1):S48-S49.
84. Santos R, Barros VV, Igai AK, et al. Maternal death and Venous Thromboembolism (VTE) in patients admitted in a maternity of high risk: Results pre and post application of a risk score. *Journal of Thrombosis and Haemostasis* 2015;13(SUPPL. 2):679. doi: http://dx.doi.org/10.1111/jth.12993
85. Schoenbeck D, Nicolle A, Newbegin K, et al. The use of a scoring system to guide thromboprophylaxis in a high-risk pregnant population. *Thrombosis* 2011;2011:652796. doi: https://dx.doi.org/10.1155/2011/652796
86. Sellappan KV, Farr R, Hill P. An audit on risk assessment of women at antenatal booking appointment. *International Journal of Gynecology and Obstetrics* 2012;119(SUPPL. 3):S703-S04. doi: http://dx.doi.org/10.1016/S0020-7292%2812%2961724-X

For peer review only

87. Shacaluga A, Wallace P, Rayment R. Abandoning RCOG Guideline 37a: A risk worth taking? *BJOG: An International Journal of Obstetrics and Gynaecology* 2017;124(Supplement 1):142. doi: http://dx.doi.org/10.1111/1471-0528.9_14572

88. Tan EK, Wisdom SJ. Thromboprophylaxis post vaginal delivery: are we forgetting it? Audit on thromboprophylaxis prescription post vaginal births. *Journal of Obstetrics and Gynaecology: The Journal of the Institute of Obstetrics and Gynaecology* 2006;26(1):27-9.

89. Tang Z, Marsden PJ. Risk assessment and management of venous thrombo-embolism in obstetric women. *Archives of Disease in Childhood: Fetal and Neonatal Edition* 2011;96(SUPPL. 1) doi: http://dx.doi.org/10.1136/adc.2011.300163.65

90. Taylor GM, McKenzie CA, Mires GJ. Use of a computerised maternity information system to improve clinical effectiveness: thromboprophylaxis at caesarean section. *Postgraduate Medical Journal* 2000;76(896):354-6.

91. Testa S, Paoletti O, Ronca E, et al. Health care program for thromboembolism prevention in pregnancy. *Pathophysiology of Haemostasis and Thrombosis* 2010;37(SUPPL. 1):A76. doi: http://dx.doi.org/10.1159/000318097

92. Testa S, Passamonti SM, Paoletti O, et al. The pregnancy health-care program: A model for the prevention of venous thromboembolism in pregnancy. *Journal of Thrombosis and Haemostasis* 2013;11(SUPPL. 2):606.

93. Touhami O, Marzouk SB, Bennasr L, et al. Are the Wells Score and the Revised Geneva Score valuable for the diagnosis of pulmonary embolism in pregnancy? *European Journal of Obstetrics, Gynecology, and Reproductive Biology* 2018;221:166-71. doi: https://dx.doi.org/10.1016/j.ejogrb.2017.12.049

94. Usoro E, Sakurai H, Aedla N. Investigation and management of venous thromboembolism in pregnancy. *European Journal of Obstetrics Gynecology and Reproductive Biology* 2019;234:e105. doi: http://dx.doi.org/10.1016/j.ejogrb.2018.08.390

95. Valdre L, Lambertini I, Palareti G, et al. Comparison of strategies for preventing venous thromboembolism in high risk pregnant women according to national and international guidelines: Results of a prospective cohort study. *Blood Transfusion* 2016;14(Supplement 5):s747.

96. Von Hawrylak F. Implementing a venous thromboembolism risk assessment in an abortion service. *European Journal of Contraception and Reproductive Health Care* 2018;23(Supplement 1):43. doi: http://dx.doi.org/10.1080/13625187.2018.1442911

97. Zhang W, Shen J, Sun J-L. Risk scores, prevention, and treatment of maternal venous thromboembolism. *World Journal of Clinical Cases* 2020;8(11):2210-18. doi: https://dx.doi.org/10.12998/wjcc.v8.i11.2210

For peer review only

**APPENDIX S3 SIMPLIFIED SUMMARY OF WIDELY EVALUATED GENERIC RAMS, THEIR ASSOCIATED CHARACTERISTICS AND COMPOSITE CLINICAL VARIABLES**

| Characteristics | Name of VTE risk assessment model | | | |
|---|---|---|---|---|
| | RCOG | ACOG | SFOG | Lyon score |
| General | | | | |
| Author, year | Royal College of Obstetricians and Gynaecologists, 2015[1] | James et al., 2018[2] | Lindquist et al., 2008[3] and Lindqvist & Hellgren, 2011[4] | Dargaud et al., 2017[5] |
| Applicable cohort | All pregnant and postpartum women | All pregnant and postpartum women at risk | Pregnant women with moderate-high risk of VTE | Pregnant women with high risk of thrombosis |
| Design | Risk factor based with cumulative score | Risk factor based | Risk factor based with cumulative score | Risk factor based with cumulative score |
| Number of VTE risk variables | 26 | Not specified | 23 | 15 |
| When is pharmacological thromboprophylaxis recommended? | • Score ≥ 4 antenatally (from first trimester)<br>• Score 3 antenatally (from 28 weeks)<br>• Score ≥ 2 postnatally (at least 10 days).<br>• Antenatal hospital admission<br>• Prolonged hospital admission (≥ 3 days) or readmission to hospital within puerperium | • All women with acute VTE during pregnancy, or women with history of thrombosis or those at significant risk of VTE during pregnancy or the postpartum period such as those with thrombophilia | • Very high risk (high dose antepartum and at least 12 weeks postpartum)[a]<br>• Score ≥ 4 (antepartum and 6 weeks postpartum)<br>• Score 3 (after delivery [6 weeks])<br>• Score 2 (after delivery [7 days] or during immobilisation) | • Score ≥ 6 antenatally or postnatally<br>• Score between 3 and 5, from third trimester |
| **Pre-existing risk factors** | | | | |

For peer review only

| | | | | |
|---|---|---|---|---|
| Previous VTE (personal) | Yes (except a single event related to major surgery) | Yes | Yes | Yes (pregnancy related, CVT or massive PE or VTE in childhood [<16 years]; unprovoked or oestrogen related; transient risk factor induced) |
| Recurrent VTE | No | Yes | Yes | Yes (personal history; residual venous thrombi with clinical signs of PTS, recent <2 years) |
| Previous VTE provoked by specific event | Yes (major surgery) | Yes (surgery, trauma or immobility AND additional major thrombotic risk factors)[b] | No | No |
| Family history of VTE | Yes (unprovoked or estrogen related) | Yes (first degree with thrombophilia) | Yes (first degree <60 years) | Yes (severe or recurrent) |
| Thrombophilia e.g. Factor V Leiden and Factor II mutations; protein C, protein S and antithrombin deficiency; antiphospholipid syndrome (with or without VTE) | Yes (various forms | Yes (various forms) | Yes (various forms) | Yes (various forms) |
| Medical comorbidities | Yes (3 points for any individual comorbidities) | No | Yes (inflammatory bowel disease) | No |
| Age | Yes (>35 years) | No | Yes (>40 years) | Yes (>35 years) |
| Obesity | Yes (≥30kg/m$^2$; ≥40kg/m$^2$) | No | Yes (>28kg/m$^2$ in early pregnancy) | Yes (≥30kg/m$^2$) |

For peer review only

| | | | | |
|---|---|---|---|---|
| Parity | Yes (≥3) | No | No | No |
| Smoker | Yes | No | No | No |
| Varicose veins | Yes (gross) | No | No | No |
| Hyperhomocysteinemia | No | No | Yes (homocysteine >8 $\mu$mol/L in pregnancy | No |
| Mechanical heart prosthesis | No | No | Yes | No |
| Chronic warfarin prophylaxis | No | No | Yes | No |
| **Obstetric** | | | | |
| Pre-eclampsia | Yes (current pregnancy) | No | Yes | No |
| ART/IVF | Yes (antenatal only) | No | No | No |
| Multiple pregnancy | Yes | No | No | Yes |
| Caesarean section | Yes (elective/in labour) | No | Yes | No |
| Mid-cavity or rotational operative delivery | Yes | No | No | No |
| Prolonged labour (> 24 hours) | Yes | No | No | No |
| Postpartum haemorrhage | Yes (>1 litre or transfusion) | No | No | No |
| Preterm birth | Yes (<37 weeks, current pregnancy) | No | No | No |
| Stillbirth | Yes (current pregnancy) | No | No | No |
| Abruptio placenta | No | No | Yes | No |
| **Transient factors** | | | | |
| Any surgical procedure | Yes (pregnancy or puerperium except immediate repair of the perineum) | No | No | No |
| Hyperemesis | Yes | No | No | No |

For peer review only

| | | | | |
|---|---|---|---|---|
| Ovarian hyperstimulation syndrome | Yes (first trimester only) | No | No | No |
| Systemic infection | Yes (current) | No | No | No |
| Immobility | Yes (current and dehydration) | No | Yes | Yes |
| **Other** | | | | |
| 'Other risk factors' | No | No | Yes (according to clinical decision) | No |

[a] Thromboprophylaxis initiated as early as possible (sometimes before pregnancy). Only women with antithrombin deficiency, chronic warfarin prophylaxis, recurrent VTE, antiphospholipid syndrome with VTE, and those with mechanical heart prosthesis are included in this group

[b] First-degree relative with a history of a thrombotic episode, or other major thrombotic risk factors (e.g., obesity, prolonged immobility, caesarean delivery

ACOG, American College of Obstetricians and Gynaecologists; ART/IVF Assisted reproductive technology/ In vitro fertilization; RCOG, Royal College of Obstetricians and Gynaecologists; SFOG, Swedish Society of Obstetrics and Gynecology; VTE, venous thromboembolism

For peer review only

**REFERENCES (APPENDIX S3)**


1. Royal College of Obstetricians & Gynaecologists. Thrombosis and Embolism during Pregnancy and the Puerperium, Reducing the Risk (Green-top Guideline No. 37a) 2015 [Available from: https://www.rcog.org.uk/en/guidelines-research-services/guidelines/gtg37a/ accessed January 2022.
2. James A, Birsner M, Kaimal A, et al. ACOG Practice Bulletin No. 196. Summary: thromboembolism in pregnancy. *Obstet Gynecol* 2018;132:243-8.
3. Lindqvist PG, Torsson J, Almqvist A, et al. Postpartum thromboembolism: severe events might be preventable using a new risk score model. *Vascular Health and Risk Management* 2008;4(5):1081-7.
4. Lindqvist PG, Hellgren M. Obstetric thromboprophylaxis: the Swedish guidelines. *Advances in Hematology* 2011;2011:157483. doi: https://dx.doi.org/10.1155/2011/157483
5. Dargaud Y, Rugeri L, Fleury C, et al. Personalized thromboprophylaxis using a risk score for the management of pregnancies with high risk of thrombosis: a prospective clinical study. *Journal of Thrombosis and Haemostasis* 2017;15(5):897-906.

For peer review only

# PRISMA 2020 Checklist

| Section and Topic | Item # | Checklist item | Location where item is reported |
|---|---|---|---|
| **TITLE** | | | |
| Title | 1 | Identify the report as a systematic review. | Title page (page 1) |
| **ABSTRACT** | | | |
| Abstract | 2 | See the PRISMA 2020 for Abstracts checklist. | Abstract (pages 2-3) |
| **INTRODUCTION** | | | |
| Rationale | 3 | Describe the rationale for the review in the context of existing knowledge. | Introduction (page 5-6) |
| Objectives | 4 | Provide an explicit statement of the objective(s) or question(s) the review addresses. | Introduction (page 5-6) |
| **METHODS** | | | |
| Eligibility criteria | 5 | Specify the inclusion and exclusion criteria for the review and how studies were grouped for the syntheses. | Eligibility criteria (page 6) |
| Information sources | 6 | Specify all databases, registers, websites, organisations, reference lists and other sources searched or consulted to identify studies. Specify the date when each source was last searched or consulted. | Data sources and searches (pages 6-7) |
| Search strategy | 7 | Present the full search strategies for all databases, registers and websites, including any filters and limits used. | Appendix S1 |
| Selection process | 8 | Specify the methods used to decide whether a study met the inclusion criteria of the review, including how many reviewers screened each record and each report retrieved, whether they worked independently, and if applicable, details of automation tools used in the process. | Study selection (page 7) |
| Data collection process | 9 | Specify the methods used to collect data from reports, including how many reviewers collected data from each report, whether they worked independently, any processes for obtaining or confirming data from study investigators, and if applicable, details of automation tools used in the process. | Data abstraction and quality assessment (pages 7-8) |
| Data items | 10a | List and define all outcomes for which data were sought. Specify whether all results that were compatible with each outcome domain in each study were sought (e.g. for all measures, time points, analyses), and if not, the methods used to decide which results to collect. | Data abstraction and quality assessment (pages 7-8) |
| | 10b | List and define all other variables for which data were sought (e.g. participant and intervention characteristics, funding sources). Describe any assumptions made about any missing or unclear information. | Data abstraction and quality assessment (pages 7-8) |
| Study risk of bias assessment | 11 | Specify the methods used to assess risk of bias in the included studies, including details of the tool(s) used, how many reviewers assessed each study and whether they worked independently, and if applicable, details of automation tools used in the process. | Data abstraction and quality assessment (pages 7-8) |
| Effect measures | 12 | Specify for each outcome the effect measure(s) (e.g. risk ratio, mean difference) used in the synthesis or presentation of results. | Data synthesis and analysis (page 8-9) |
| Synthesis methods | 13a | Describe the processes used to decide which studies were eligible for each synthesis (e.g. tabulating the study intervention characteristics and comparing against the planned groups for each synthesis (item #5)). | Data synthesis and analysis (page 8-9) |
| | 13b | Describe any methods required to prepare the data for presentation or synthesis, such as handling of missing summary statistics, or data conversions. | Data synthesis and analysis (page 8-9) |

For peer review only

# PRISMA 2020 Checklist

| Section and Topic | Item # | Checklist item | Location where item is reported |
|---|---|---|---|
| | 13c | Describe any methods used to tabulate or visually display results of individual studies and syntheses. | Data synthesis and analysis (page 8-9) |
| | 13d | Describe any methods used to synthesize results and provide a rationale for the choice(s). If meta-analysis was performed, describe the model(s), method(s) to identify the presence and extent of statistical heterogeneity, and software package(s) used. | Data synthesis and analysis (page 8-9) |
| | 13e | Describe any methods used to explore possible causes of heterogeneity among study results (e.g. subgroup analysis, meta-regression). | Data synthesis and analysis (page 8-9) |
| | 13f | Describe any sensitivity analyses conducted to assess robustness of the synthesized results. | Data synthesis and analysis (page 8-9) |
| Reporting bias assessment | 14 | Describe any methods used to assess risk of bias due to missing results in a synthesis (arising from reporting biases). | Data synthesis and analysis (page 8-9) |
| Certainty assessment | 15 | Describe any methods used to assess certainty (or confidence) in the body of evidence for an outcome. | N/A |
| **RESULTS** | | | |
| Study selection | 16a | Describe the results of the search and selection process, from the number of records identified in the search to the number of studies included in the review, ideally using a flow diagram. | Study flow (pages 9) |
| | 16b | Cite studies that might appear to meet the inclusion criteria, but which were excluded, and explain why they were excluded. | Appendix S2 |
| Study characteristics | 17 | Cite each included study and present its characteristics. | Study and patient characteristics (pages 10-11) |
| Risk of bias in studies | 18 | Present assessments of risk of bias for each included study. | Risk of bias and applicability assessment (pages 13-15) |
| Results of individual studies | 19 | For all outcomes, present, for each study: (a) summary statistics for each group (where appropriate) and (b) an effect estimate and its precision (e.g. confidence/credible interval), ideally using structured tables or plots. | Predictive performance of VTE RAMs (summary of results) (pages 16-19) |
| Results of syntheses | 20a | For each synthesis, briefly summarise the characteristics and risk of bias among contributing studies. | Risk of bias and applicability assessment (pages 13-15) and Predictive performance of VTE RAMs (summary of results) (pages 16-19) |
| | 20b | Present results of all statistical syntheses conducted. If meta-analysis was done, present for each the summary estimate and its precision (e.g. confidence/credible interval) and measures of statistical heterogeneity. If comparing groups, describe the direction of the effect. | Predictive performance of VTE RAMs (summary of results) (pages 16-19) |
| | 20c | Present results of all investigations of possible causes of heterogeneity among study results. | Predictive performance of VTE RAMs (summary of results) (pages 16-19) |
| | 20d | Present results of all sensitivity analyses conducted to assess the robustness of the synthesized results. | N/A |
| Reporting biases | 21 | Present assessments of risk of bias due to missing results (arising from reporting biases) for each synthesis assessed. | Predictive performance of VTE RAMs (summary of results) (pages 16-19) |

For peer review only

For peer review only - http://bmjopen.bmj.com/site/about/guidelines.xhtml

# PRISMA 2020 Checklist

| Section and Topic | Item # | Checklist item | Location where item is reported |
|---|---|---|---|
| Certainty of evidence | 22 | Present assessments of certainty (or confidence) in the body of evidence for each outcome assessed. | N/A |
| **DISCUSSION** | | | |
| Discussion | 23a | Provide a general interpretation of the results in the context of other evidence. | Discussion – Summary of results (page 20); Interpretation of results (pages 20-21); and Comparison to the existing literature (page 21) |
| | 23b | Discuss any limitations of the evidence included in the review. | Discussion – Interpretation of results (pages 20-21); and Comparison to the existing literature (page 21) |
| | 23c | Discuss any limitations of the review processes used. | Discussion – Strengths and limitations (page 21-23) |
| | 23d | Discuss implications of the results for practice, policy, and future research. | Discussion - Implications for patients, clinicians and policymakers and future research (pages 23-24) |
| **OTHER INFORMATION** | | | |
| Registration and protocol | 24a | Provide registration information for the review, including register name and registration number, or state that the review was not registered. | Page 3 and 6 |
| | 24b | Indicate where the review protocol can be accessed, or state that a protocol was not prepared. | Page 3 and 6 |
| | 24c | Describe and explain any amendments to information provided at registration or in the protocol. | N/A |
| Support | 25 | Describe sources of financial or non-financial support for the review, and the role of the funders or sponsors in the review. | Funding section (page 25) |
| Competing interests | 26 | Declare any competing interests of review authors. | Competing interest section (page 25) |
| Availability of data, code and other materials | 27 | Report which of the following are publicly available and where they can be found: template data collection forms; data extracted from included studies; data used for all analyses; analytic code; any other materials used in the review. | All data relevant to the study are included in the article or uploaded as Appendices (page 25– see data sharing statement) |

*From:* Page MJ, McKenzie JE, Bossuyt PM, Boutron I, Hoffmann TC, Mulrow CD, et al. The PRISMA 2020 statement: an updated guideline for reporting systematic reviews. BMJ 2021;372:n71. doi: 10.1136/bmj.n71

For more information, visit: http://www.prisma-statement.org/

For peer review only

# Risk assessment models for venous thromboembolism in pregnancy and in the puerperium: a systematic review



| | |
|---|---|
| Journal: | *BMJ Open* |
| Manuscript ID | bmjopen-2022-065892.R1 |
| Article Type: | Original research |
| Date Submitted by the Author: | 13-Sep-2022 |
| Complete List of Authors: | Pandor, Abdullah; The University of Sheffield, ScHARR<br>Daru, Jahnavi; Queen Mary University of London, Institute of Population Health Sciences<br>Hunt, Beverley; Guy's and St Thomas' NHS Foundation Trust, Department of Haematology<br>Rooney, Gill; The University of Sheffield, ScHARR<br>Hamilton, Jean; The University of Sheffield, ScHARR<br>Clowes, Mark; The University of Sheffield, ScHARR<br>Goodacre, Steve; The University of Sheffield, ScHARR<br>Nelson-Piercy, Catherine; Guy's and St Thomas' NHS Foundation Trust<br>Davis, Sarah; The University of Sheffield, ScHARR |
| <b>Primary Subject Heading</b>: | Obstetrics and gynaecology |
| Secondary Subject Heading: | Haematology (incl blood transfusion) |
| Keywords: | OBSTETRICS, HAEMATOLOGY, Maternal medicine < OBSTETRICS, Anticoagulation < HAEMATOLOGY |
| | |



SCHOLARONE™
Manuscripts

*I, the Submitting Author has the right to grant and does grant on behalf of all authors of the Work (as defined in the below author licence), an exclusive licence and/or a non-exclusive licence for contributions from authors who are: i) UK Crown employees; ii) where BMJ has agreed a CC-BY licence shall apply, and/or iii) in accordance with the terms applicable for US Federal Government officers or employees acting as part of their official duties; on a worldwide, perpetual, irrevocable, royalty-free basis to BMJ Publishing Group Ltd ("BMJ") its licensees and where the relevant Journal is co-owned by BMJ to the co-owners of the Journal, to publish the Work in this journal and any other BMJ products and to exploit all rights, as set out in our licence.*

*The Submitting Author accepts and understands that any supply made under these terms is made by BMJ to the Submitting Author unless you are acting as an employee on behalf of your employer or a postgraduate student of an affiliated institution which is paying any applicable article publishing charge ("APC") for Open Access articles. Where the Submitting Author wishes to make the Work available on an Open Access basis (and intends to pay the relevant APC), the terms of reuse of such Open Access shall be governed by a Creative Commons licence – details of these licences and which Creative Commons licence will apply to this Work are set out in our licence referred to above.*

*Other than as permitted in any relevant BMJ Author's Self Archiving Policies, I confirm this Work has not been accepted for publication elsewhere, is not being considered for publication elsewhere and does not duplicate material already published. I confirm all authors consent to publication of this Work and authorise the granting of this licence.*

For peer review only

**Complete manuscript title:**

# Risk assessment models for venous thromboembolism in pregnancy and in the puerperium: a systematic review

**Short title: Predicting VTE in pregnancy**

**Authors:**

Abdullah Pandor, ScHARR, The University of Sheffield, Sheffield, UK

Jahnavi Daru, Institute of Population Health Sciences, Queen Mary University of London, UK

Beverley J Hunt, Guy's and St Thomas' NHS Foundation Trust, London, UK

Gill Rooney, ScHARR, The University of Sheffield, Sheffield, UK

Jean Hamilton, ScHARR, The University of Sheffield, Sheffield, UK

Mark Clowes, ScHARR, The University of Sheffield, Sheffield, UK

Steve Goodacre, ScHARR, The University of Sheffield, Sheffield, UK

Catherine Nelson-Piercy, Guy's and St Thomas' NHS Foundation Trust, London, UK

Sarah Davis, ScHARR, The University of Sheffield, Sheffield, UK

**Corresponding author:** Abdullah Pandor, BSc (Hons), MSc, ScHARR, The University of Sheffield, Regent Court, 30 Regent Street, Sheffield, S1 4DA, telephone +44 (0) 114 222 0778, email: a.pandor@sheffield.ac.uk

**Word count: 3682**

For peer review only

## ABSTRACT

### OBJECTIVES

To assess the comparative accuracy of risk assessment models (RAMs) to identify women during pregnancy and the early postnatal period who are at increased risk of VTE.

### DESIGN

Systematic review following Preferred Reporting Items for Systematic Reviews and Meta-Analyses guidelines.

### DATA SOURCES

MEDLINE, EMBASE, Cochrane Library and two research registers were searched until February 2021.

### ELIGIBILITY CRITERIA

All validation studies that examined the accuracy of a multivariable RAM (or scoring system) for predicting the risk of developing VTE in women who are pregnant or in the puerperium (within 6-weeks post-delivery).

### DATA EXTRACTION AND SYNTHESIS

Two authors independently selected and extracted data. Risk of bias was appraised using PROBAST tool. Data were synthesised without meta-analysis.

### RESULTS

Seventeen studies, comprising 19 externally validated RAMs and one internal validated model met the inclusion criteria. The most widely evaluated RAMs were the RCOG guidelines (6 studies), ACOG guidelines (2 studies), SFOG guidelines (2 studies) and the Lyon score (2 studies). In general,


For peer review only

estimates of sensitivity and specificity were highly variable with sensitivity estimates ranging from 0% to 100% for RAMs that were applied to antepartum women to predict antepartum or postpartum VTE and 0% to 100% for RAMs applied postpartum to predict postpartum VTE. Specificity estimates were similarly diverse ranging from 28% to 98% and 5% to 100%, respectively.

**CONCLUSIONS**

Available data suggest that external validation studies have weak designs and limited generalisability, so estimates of prognostic accuracy are very uncertain.

**PROSPERO REGISTRATION**

Abdullah Pandor, Steve Goodacre, Gill Rooney, Mark Clowes, Sarah Davis. A systematic review of risk assessment models for venous thromboembolism in pregnancy and in the puerperium. PROSPERO 2020 CRD42020221094 Available from: https://www.crd.york.ac.uk/prospero/display_record.php?ID=CRD42020221094

**KEYWORDS**

Pregnancy, postpartum period, risk assessment, systematic review, venous thromboembolism

For peer review only

## ARTICLE SUMMARY

### Strengths and limitations of this study

- A number of risk assessment models for venous thromboembolism (VTE) in pregnancy & puerperium have been developed using a variety of methods and based on a variety of predictor variables.
- This systematic review provides a comprehensive review of risk assessment models for predicting the risk of developing VTE in women who are pregnant or in the puerperium (within 6 weeks post-delivery).
- The newly developed PROBAST tool was used to evaluate the risk of bias and applicability of the available evidence
- Heterogeneity in the included studies (participants, inclusion criteria, clinical condition, outcome definition and measurement) and variable reporting of items precluded meta-analysis.
- Limitations of the existing evidence and areas of future research are highlighted.



For peer review only - http://bmjopen.bmj.com/site/about/guidelines.xhtml

## INTRODUCTION

Venous thromboembolism (VTE) remains an important cause of maternal morbidity and mortality in the developed world.[1] Whilst uncommon, VTE complications can occur at a rate of 1 to 2 per 1000 deliveries and can develop at any time during pregnancy.[2-4] The risks substantially increase during the post-partum period (6 weeks post-delivery)[5] and can be as high as 60-fold in some individuals compared with age-matched non-pregnant women.[6] Preventative treatment with low-dose anticoagulation (thromboprophylaxis) has the potential to reduce the risk of symptomatic and asymptomatic VTE in pregnancy and the postpartum period.[5] Consequently, various prominent international guidelines recommend targeted thromboprophylaxis for pregnant and puerperal women deemed to be at high risk of VTE.[5, 7-13] However, these expert-based consensus guidelines vary substantially with regards to the threshold of risk (based on certain risk factors) and the timing, dose and duration of pharmacological thromboprophylaxis.

Risk assessment models (RAMs) have been developed to help stratify the risk of VTE during pregnancy and the early postnatal period. These models use clinical information from the patient's history and examination to identify those with an increased risk of developing VTE who are most likely to benefit from pharmacological thromboprophylaxis. Inappropriate use of VTE prophylaxis may not reduce VTE rates and may cause unnecessary harm especially through bleeding and bruising.[14] Whilst RAMs could improve the ratio of benefit to risk and benefit to cost, it is unclear which VTE RAM are best  applied to guide decision-making for thromboprophylaxis in clinical practice and thereby optimise patient care.



For peer review only - http://bmjopen.bmj.com/site/about/guidelines.xhtml

The aim of this systematic review was to identify primary validation studies and determine the accuracy of individual RAMs that identify pregnant and postpartum women at increased risk of developing VTE who could be selected for thromboprophylaxis.

## METHODS

A systematic review was undertaken in accordance with the general principles recommended in the Preferred Reporting Items for Systematic Reviews and Meta-Analyses (PRISMA) statement.[15] This review was part of a larger project on Thromboprophylaxis in pregnancy and after delivery[16] and was registered on the International Prospective Register of Systematic Reviews (PROSPERO) database (CRD42020221094).

### Eligibility criteria

All studies evaluating the accuracy (e.g. sensitivity, specificity, C-statistic) of a multivariable RAM (or scoring system) for predicting the risk of developing VTE were eligible for inclusion. We primarily sought and selected studies that included validation of the model in a group of patients that were not involved in the development of the prediction model. Although the included studies could have reported derivation of the model (for internal validation), we only used the external validation data to estimate accuracy, where appropriate. The study population of interest in our review consisted of pregnant and postpartum (within 6 weeks post-delivery) women who are at increased risk of developing a VTE and receiving care in both hospital, community, and primary care settings. Studies that focussed on non-pregnant women were excluded as these patient groups have VTE risk profiles that differ markedly from the obstetric population.

### Data sources and searches



For peer review only - http://bmjopen.bmj.com/site/about/guidelines.xhtml

Potentially relevant studies were identified through searches of several electronic databases and research registers. This included MEDLINE (OvidSP from 1946), EMBASE (OvidSP from 1974), the Cochrane Library (https://www.cochranelibrary.com from inception), ClinicalTrials.gov (US National Institutes of Health from 2000) and the International Clinical Trials Registry Platform (World Health Organisation from 1990). All searches were conducted from inception to February 2021. The search strategy used free text and thesaurus terms and combined synonyms relating to the condition (e.g. VTE in pregnant and postpartum women) with risk prediction modelling terms.[17] No language or date restrictions were used. Searches were supplemented by hand-searching the reference lists of all relevant studies (including existing systematic reviews); forward citation searching of included studies; contacting key experts in the field; and undertaking targeted searches of the World Wide Web using the Google search engine. Further details on the search strategy can be found in the Appendix S1 (supplementary information).

**Study selection**

All titles were examined for inclusion by one reviewer (GR) and any citations that clearly did not meet the inclusion criteria (e.g. non-human, unrelated to VTE in pregnancy and the puerperium) were excluded (for quality assurance a random subset of 20% was checked by a second reviewer). All abstracts and full text articles were then examined independently by two reviewers (GR and AP). Any disagreements in the selection process were resolved through discussion or if necessary, arbitration by a third reviewer (JD) or the wider group (BH, CNP, SG) and included by consensus.

**Data extraction and quality assessment**



For peer review only - http://bmjopen.bmj.com/site/about/guidelines.xhtml

For eligible studies, data relating to study design, methodological quality and outcomes were extracted by one reviewer (GR) into a standardised data extraction form and independently checked for accuracy by a second reviewer (AP). Any discrepancies were resolved through discussion, or if this was unsuccessful, a third reviewer's opinion was sought (JD). Where multiple publications of the same study were identified, data were extracted and reported as a single study.

The methodological quality of each included study was assessed using PROBAST (Prediction model Risk Of Bias ASsessment Tool).[18, 19] This instrument includes four key domains: participants (e.g., study design and patient selection), predictors (e.g., differences in definition and measurement of the predictors), outcome (e.g., differences related to the definition and outcome assessment), and statistical analysis (e.g., sample size, choice of analysis method and handling of missing data). Each domain is assessed in terms of risk of bias and the concern regarding applicability to the review (first three domains only). To guide the overall domain-level judgement about whether a study is at high, low or an unclear (in the event of insufficient data in the publication to answer the corresponding question) risk of bias, sub-domains within each domain include several signalling questions to help judge with bias and applicability concerns. An overall risk of bias for each individual study was defined as low risk when all domains were judged as low; and high risk of bias when one or more domains were considered as high. Studies were assigned an unclear risk of bias if one or more domains were unclear, and all other domains were low.

**Data synthesis and analysis**

Due to significant levels of heterogeneity between studies (study design, participants, inclusion criteria) and variable reporting of items, a meta-analysis was not considered



For peer review only - http://bmjopen.bmj.com/site/about/guidelines.xhtml

possible. As a result, a pre-specified narrative synthesis approach[20, 21] was undertaken, with data being summarised in tables with accompanying narrative summaries that included a description of the included variables, statistical methods and performance measures (e.g. sensitivity, specificity and C-statistic [a value between 0.7 to 0.8 and >0.8 indicated good and excellent discrimination, respectively; and values <0.7 were considered weak]),[22] where applicable. All analyses were conducted using Microsoft Excel 2010 (Microsoft Corporation, Redmond, WA, USA).

For peer review only

**Patient and public involvement**

Patients and the public were not involved in the design or conduct of this systematic review.

## RESULTS

**Study flow**

**Figure 1** summarises the process of identifying and selecting relevant literature. Of the 2268 citations identified, 16 studies[23-38] investigating 19 unique externally validated RAMs met the inclusion criteria. Only one of these studies[35] presented data on model development and external validation (this study used UK Clinical Practice Research Data linked to Hospital Episode Statistics to develop a risk prediction model and externally validated using Swedish medical birth registry data). The remaining studies focused on external validation with no description of the initial derivation methodology.[23-34, 36-38] Due to the lack of model derivation studies with external validation, we also identified and included one internal validation study for completeness (i.e. prediction model development without external validation).[39] This study used a boot-strap validation approach to capture optimism in model performance[40, 41] when applied to similar future patients. Most of the full-text articles (n=97) were excluded primarily based on not using a RAM for predicting the risk of developing VTE during



For peer review only - http://bmjopen.bmj.com/site/about/guidelines.xhtml

pregnancy or the puerperium, having no useable or relevant outcome data or an inappropriate study design (e.g., reviews, commentaries, or study protocols). A full list of excluded studies with reasons for exclusion is provided in Appendix S2 (supplementary information).

**[INSERT FIGURE 1]**

**Study and patient characteristics**

The design and participant characteristics of the 17 included studies are summarised in **Table 1**. All studies were published between 2000 and 2020 and were undertaken in North America (n=4),[24, 37-39] Southeast Asia (n=1),[33] Europe (n=10),[23, 25-30, 32, 34, 36] South America (n=1),[31] and one study was multi-country.[35] Sample sizes ranged from 52[31] to 662,387[35] patients in 14 observational cohort studies (6 prospective[25, 27, 28, 31, 33, 36] [all single centre] and 8 retrospective[24, 26, 29, 30, 34, 35, 37, 39] [2 of which were multicentre] in design). Sample sizes in two, single centre case control studies[32, 38] ranged from 76[38] to 2421[32] patients and one study used a non-randomised multicentre study design.[23] The mean age ranged from 27.8 years[39] to 34 years[25, 29] (not reported in 7 studies).[24, 27, 32, 34, 36-38]

The majority of studies were conducted across antenatal and postnatal periods,[23, 27-29, 31, 34, 36, 38] or postpartum period only[24-26, 30, 32, 33, 35, 37, 39] and generally included women at increased risk of VTE.[23-25, 28, 29, 31-33, 38, 39] One study excluded women with a history of VTE[35] and six studies[26, 27, 30, 34, 36, 37] included all pregnant women who delivered. Thromboprophylaxis was employed in about half (n=9)[23, 25, 28-31, 33, 35, 36] of the studies, with the proportion receiving thromboprophylaxis ranging from 3%[35] to 100%.[23, 28] The remaining studies did not report data on thromboprophylaxis use.



For peer review only - http://bmjopen.bmj.com/site/about/guidelines.xhtml

**Table 1: Study and population characteristics**

| Author, year | Country | Design | Single/ multi-centre | Sample size | Population | Period | Mean age (years) | VTE prophylaxis | RAMs evaluated | Target condition, definition (risk period) | Incidence |
|---|---|---|---|---|---|---|---|---|---|---|---|
| Antepartum and postpartum following vaginal and caesarean delivery | | | | | | | | | | | |
| Bauersachs et al., 2007[23] | Germany | P,NRS | Multi | 810 | Women at increased risk of VTE (due to thromboembolic status and prior VTE) | March 1999 to December 2002 | 30.8 | 100% | •EThIG | Antepartum and postpartum VTE, symptomatic (NR) | 0.62% (antepartum: 0.25%; postpartum: 0.37%) |
| Chauleur et al., 2008[27] | France | P,CS | Single | 2685 | All women who delivered | July 2002-June 2003 | NR (median, 29) | NR | •STRATHEGE | Antepartum and postpartum VTE (NR) | 0.34% (antepartum: 0.19%; postpartum: 0.15%) |
| Dargaud et al., 2017[28] | France | P,CS | Single | 445 | Women at increased risk of VTE (due to thromboembolic status and prior VTE) | January 2005-January 2015 | 33 | 100% | •Lyon | Antepartum and postpartum VTE, not defined (pregnancy and 3 months postpartum) | 1.35% |
| Dargaud et al., 2005[29] | France | R,CS | Single | 116 | Women at increased risk of VTE (due to thromboembolic status and prior VTE) | 2001-2003 | 34 | 53% | •Lyon | Antepartum and postpartum VTE, not defined (NR) | 0.86% (antepartum only) |
| Hase et al., 2018[31] | Brazil | P,CS | Single | 52 | Hospitalised pregnant women with cancer | 1 December 2014 to 31 July 2016 | 31 | 57.7% | •RCOG (modified) | Antepartum and postpartum VTE, not defined (pregnancy and 3 months postpartum) | Unable to estimate - no VTE |
| Shacaluga et al., 2019[34] (correspondence) | Wales | R,CS | Single | 42,000 | All managed pregnancies | 2009-2015 | NR | NR | •All Wales<br>•RCOG | Antepartum and postpartum VTE, not defined, (NR) | 0.08% (antepartum: 0.04%; postpartum: 0.04%) |
| Testa et al., 2015[36] | Italy | P,CS | Single | 1719 | All pregnant women enrolled in Pregnancy Healthcare Program | January 2008 to December 2010 | NR (median 33) | 4.6% | •Novel (Testa) | Antepartum and postpartum VTE (NR) | Unable to estimate - no VTE |
| Weiss et al., 2000[38] | USA | CC | Single | 19 cases: 57 control[a] | Women with (confirmed cases) and without (unmatched control) VTE | 1987-1998 | NR | NR | •Novel (Weiss) | Antepartum and postpartum VTE, not defined (pregnancy and 6 weeks postpartum) | - |
| Postpartum only following vaginal and caesarean delivery | | | | | | | | | | | |
| Chau et al., 2019[26] | France | R,CS | Single | 1069 (time period 2012: 557; 2015: 512) | All women who delivered | February-April 2012 and February-April 2015 | 2012: 29<br>2015: 29 | NR | •Novel (Chau) | Postpartum VTE, not defined (8 weeks) | 2012: 0.18%<br>2015: 0.20% |
| Ellis-Kahana et al., 2020[39b] | USA | R,CS | Multi | 83,500 | All obese women (BMI >30kg/m$^2$) who delivered | 2002-2008 | 27.8 | NR | •Novel (Ellis-Kahana) | Postpartum VTE (NR) | 0.13% |
| Gassmann et al., 2020[30] | Switzerland | R,CS[c] | Single | 344 | All women who delivered | 1-31 January 2019 | 32.2 | 24% | •RCOG<br>•ACOG<br>•ACCP | Postpartum VTE, not defined (3 months) | Unable to estimate - no VTE |



For peer review only - http://bmjopen.bmj.com/site/about/guidelines.xhtml

| | | | | | | | | | | | |
|---|---|---|---|---|---|---|---|---|---|---|---|
| | | | | | | | | | •ASH | | |
| Lindqvist et al., 2008[32] | Sweden | CC | Single | 37 cases: 2384 control | All women with (confirmed cases) and without (unselected population-based control) VTE | 1990-2005 | NR | NR | •SFOG (Swedish guidelines) | Postpartum VTE (NR) | - |
| Sultan et al., 2016[35] | England (derivation)[d] and, Sweden (validation) | R,CS | Multi | 662,387 (validation cohort)[d] | All women (with no history of VTE) who delivered | 1 July 2005 to 31 December 2011 | 30.32 | 3% | •Novel (Sultan) •RCOG[d] •SFOG (Swedish Guidelines) | Postpartum VTE (6 weeks) | 0.08% (validation cohort) |
| Tran et al., 2019[37] | USA | R,CS | Single | 6094 | All women who delivered after 14 weeks | 01 January 2015 to 31 December 2016 | NR | NR | •RCOG •Padua •Caprini | Postpartum VTE (6 months) | 0.05% |
| Postpartum following caesarean delivery | | | | | | | | | | | |
| Binstock & Larkin, 2019 (abstract)[24] | USA | R,CS | Single | 2875 | Postpartum women following CD | 2011 | NR | NR | •Novel (Binstock) •RCOG | Postpartum VTE, not defined (NR) | 0.38% |
| Cavazza et al., 2012[25] | Italy | P,CS | Single | 501 | Postpartum women following CD | 2007-2009 | 34 | 53.5% | •Novel (Cavazza) | Postpartum VTE, symptomatic, not defined (90 days) | 0.20% |
| Lok et al., 2019[33] | Hong Kong | P,CS | Single | 859 | Postpartum women following CD | May 2017 to April 2018 | 32.9 | 3.3% | •Novel (Lok) •RCOG •ACOG | Postpartum VTE, symptomatic, not defined (NR) | Unable to estimate - no VTE |

Abbreviations: ACCP, American College of Chest Physicians; ACOG, American College of Obstetricians and Gynaecologists; ASH, American Society of Hematology; CC, case -control; CD, caesarean delivery; CS, cohort study; NR, not reported; NRS, non-randomised study; P, prospective; R, retrospective; RCOG, Royal College of Obstetricians and Gynaecologists; SFOG, Swedish Society of Obstetrics and Gynecology; VTE, venous thromboembolism

[a] Retrospective case-control study of pregnant and postpartum women but data reported for antepartum period only due to low number of postpartum VTE events (n=2)

[b] Internal validation study (i.e. prediction model development without external validation)

[c] Prospective cohort study with retrospective analysis, thus classified as retrospective cohort study

[d] RCOG was applied to an English derivation cohort, n=433,353, incidence, 0.07% (312 events)



For peer review only - http://bmjopen.bmj.com/site/about/guidelines.xhtml

### VTE definition and case ascertainment

Only a few studies[23, 27, 32, 36] defined the VTE endpoint (DVT and or PE) as being confirmed by objective testing. Of the remainder, three studies[35, 37, 39] had no objective confirmation of VTE and 10 studies[24-26, 28-31, 33, 34, 38] did not report the methods for diagnosis confirmation. Although 9 studies[23, 24, 27, 29, 32-34, 36, 39] did not report the VTE risk period, the majority of the remaining studies utilised the RAMs to predict the occurrence of VTE up to 3 months after delivery.[25, 28, 30, 31] Despite differences in study design, study participants, definitions, different criteria for the use of thromboprophylaxis and differences between doses of low molecular weight heparin (LMWH), the reported overall incidence of VTE in pregnancy and the puerperium was <1.3%.

### RAMs

The studies included in this review evaluated 19 externally validated RAMs[23-38] and one internally validated risk model.[39] While most RAMs focused solely on the estimate of thromboembolic risk, RAMs varied in design, structure, threshold, dosage and duration for pharmacological prophylaxis. In addition, the individual predictors and their weighting varied markedly between RAMs. The most commonly used tools were the Royal College of Obstetricians and Gynaecologists (RCOG) guidelines (6 studies),[24, 30, 33-35, 37] American College of Obstetricians and Gynaecologists (ACOG) guidelines (2 studies),[30, 33] Swedish Society of Obstetrics and Gynecology (SFOG) guidelines (2 studies)[32, 35] and the Lyon score (2 studies).[28, 29] A simplified summary of their associated characteristics and composite clinical variables is provided in Appendix S3 (supplementary information).

### Risk of bias and applicability assessment



For peer review only - http://bmjopen.bmj.com/site/about/guidelines.xhtml

The overall methodological quality of the 17 included studies is summarised in **Table 2 and Figure 2**. The methodological quality of the included studies was variable, with most studies having high or unclear risk of bias in at least one item of the PROBAST tool. The main risk of bias limitations was related to patient selection factors (arising from retrospective data collection,[24, 26, 29, 30, 32, 34, 37-39] unclear exclusions/ incomplete patient enrolment,[24, 26, 27, 31-34, 36, 38, 39] or unclear criteria for patients receiving VTE prophylaxis)[23, 30, 35]; predictor and outcome bias (due to a general lack of details on the definition[24-26, 28-31, 33, 34, 38] and methods of outcome determination[24, 26, 28-31, 33, 34, 37-39] and whether all predictors were available at the models intended time of use[23, 24, 29, 31, 32, 34, 36-39] or influenced by the outcome measurement)[23-28, 30-39] and analysis factors (low event rates,[23-31, 33-37, 39] unclear handling of missing data,[23-29, 31-34, 36-39] and failure in reporting relevant performance measures such as calibration and discrimination.[23-34, 36-38]

Assessment of applicability to the review question led to the majority of studies being classed either as unclear (n=13)[23, 26-30, 32, 34-39] or high (n=4)[24, 25, 31, 33] risk of inapplicability. These assessments were generally related to patient selection (highly selected study populations e.g. selected women at increased risk of VTE, caesarean delivery only, single disease pathologies, single site settings), predictors (inconsistency in definition, assessment or timing of predictors) and outcome determination.

For peer review only

# Table 2. Summary of each study's risk of bias and applicability concern using the PROBAST tool – Review authors' judgements

| Author, year | Risk of Bias | | | | Applicability | | | Overall | |
|---|---|---|---|---|---|---|---|---|---|
| | Participant selection | Predictors | Outcome | Analysis | Participant selection | Predictors | Outcome | Risk of bias | Applicability |
| Bauersachs et al., 2007[23] | ? | ? | + | - | ? | ? | + | - | ? |
| Binstock 2019[24] | ? | ? | ? | - | - | ? | ? | - | - |
| Cavazza 2012[25] | - | ? | ? | - | - | + | ? | - | - |
| Chau 2019[26] | ? | ? | ? | - | ? | ? | ? | - | ? |
| Chauleur 2008[27] | ? | ? | ? | - | ? | ? | ? | - | ? |
| Dargaud 2017[28] | ? | ? | ? | - | ? | ? | ? | - | ? |
| Dargaud et al., 2005[29] | - | ? | ? | - | ? | + | ? | - | ? |
| Ellis-Kahana 2020[39] | - | ? | ? | - | ? | ? | ? | - | ? |
| Gassmann et al., 2020[30] | ? | ? | ? | - | ? | ? | ? | - | ? |
| Hase et al., 2018[31] | ? | ? | ? | - | - | ? | ? | - | - |
| Lindqvist et al., 2008[32] | - | ? | ? | - | ? | ? | ? | - | ? |
| Lok et al., 2019[33] | ? | ? | - | - | - | + | ? | - | - |
| Shacaluga et al., 2019[34] | - | ? | ? | - | ? | ? | ? | - | ? |
| Sultan et al., 2016[35] | - | ? | + | + | + | ? | + | - | ? |
| Testa et al., 2015[36] | ? | ? | ? | - | ? | ? | ? | - | ? |
| Tran et al., 2019[37] | - | ? | ? | - | ? | ? | ? | - | ? |
| Weiss & Bernstein, 2000[38] | - | ? | ? | - | ? | ? | ? | - | ? |

+ indicates low risk of bias/low concern regarding applicability; –, indicates high risk of bias/high concern regarding applicability; and ? indicates unclear risk of bias/unclear concern regarding applicability



For peer review only - http://bmjopen.bmj.com/site/about/guidelines.xhtml

**[INSERT FIGURE 2]**

**Predictive performance of VTE RAMs (summary of results)**

**Table 3** and **Table 4** shows the sensitivity and specificity of RAMs that were applied to antepartum women to predict antepartum or postpartum VTE or applied postpartum to predict postpartum VTE, respectively, with the results grouped by RAM. However, any meaningful comparisons between these alone is difficult, without considering the models' corresponding discrimination and calibration metrics, which were not universally reported. Only one external validation study considered model discrimination and calibration.  In this study by Sultan et al.,[35] their recalibrated novel risk prediction model (also known as the Maternity Clot Risk) provided good discrimination and was able to discriminate postpartum women with and without VTE in the external Swedish cohort with a C statistic of 0.73 (95% CI: 0.71 to 0.75), and calibration, of observed and predicted VTE risk, close to ideal (calibration slope of 1.11 [95% CI: 1.01 to 1.20]). In the remaining studies, interpretation was further limited by marked heterogeneity, which was exacerbated when different thresholds were reported by different studies evaluating the same model. In general, model accuracy was generally poor, with high sensitivity usually reflecting a threshold effect, as indicated by corresponding low specificity values (and vice versa).



For peer review only - http://bmjopen.bmj.com/site/about/guidelines.xhtml

## Table 3. Performance of RAMs applied antepartum to predict VTE

| Risk assessment models | Threshold or cut-off | Endpoint | Data source | Performance measures | | | | | |
|---|---|---|---|---|---|---|---|---|---|
| | | | | TP | FP | FN | TN | Sensitivity (95% CI) | Specificity (95% CI) |
| **Predicting either antepartum or postpartum VTE** | | | | | | | | | |
| All Wales (1 study) | NR | VTE | Shacaluga et al., 2019[34] | 25 | NR | 9 | NR | 0.74 (0.57, 0.85) | NR |
| EThIG (1 study) | High/very high risk | VTE | Bauersachs et al., 2007[23] | 5 | 580 | 0 | 225 | 1.00 (0.57, 1) | 0.28 (0.25, 0.31) |
| Lyon (2 studies) | Risk Score ≥3 | VTE | Dargaud et al., 2017[28] | 5 | 282 | 1 | 157 | 0.83 (0.44, 0.97) | 0.36 (0.31, 0.4) |
| Lyon | Risk Score ≥3 | VTE | Dargaud et al., 2005[29] | 1 | 56 | 0 | 59 | 1.00 (0.21, 1) | 0.51 (0.42, 0.6) |
| RCOG (modified) (1 study) | Risk Score ≥3 | VTE | Hase et al., 2018[31] | 0 | 34 | 0 | 18 | unable to estimate - no VTE | 0.35 (0.23, 0.48) |
| STRATHEGE | Risk Score ≥3 | VTE | Chauleur et al., 2008[27] | 0 | 54 | 9 | 2622 | 0.00 (0, 0.3) | 0.98 (0.97, 0.99) |
| Testa 2015 (1 study) | Risk Score ≥2.5 | VTE | Testa et al., 2015[36] | 0 | 85 | 0 | 1634 | unable to estimate - no VTE | 0.95 (0.94, 0.96) |
| **Predicting antepartum VTE** | | | | | | | | | |
| EThIG (1 study) | High/very high risk | VTE | Bauersachs et al., 2007[23] | 2 | 583 | 0 | 225 | 1.00 (0.34, 1) | 0.28 (0.25, 0.31) |
| Lyon (1 study) | Risk Score ≥3 | VTE | Dargaud et al., 2017[28] | 1 | 286 | 1 | 157 | 0.50 (0.09, 0.91) | 0.35 (0.31, 0.4) |
| STRATHEGE (1 study) | Risk Score ≥1 | VTE | Chauleur et al., 2008[27] | 0 | 54 | 4 | 2627 | 0.00 (0, 0.49) | 0.98 (0.97, 0.99) |
| Weiss 2000 (1 study) | Risk Score ≥2 | VTE | Weiss et al., 2000[38] | 4 | 3 | 15 | 54 | 0.21 (0.09, 0.43) | 0.95 (0.86, 0.98) |
| **Predicting postpartum VTE** | | | | | | | | | |
| EThIG (1 study) | High/very high risk | VTE | Bauersachs et al., 2007[23] | 3 | 582 | 0 | 225 | 1.00 (0.44, 1) | 0.28 (0.25, 0.31) |
| Lyon (1 study) | Risk Score ≥3 | VTE | Dargaud et al., 2017[28] | 4 | 283 | 0 | 158 | 1.00 (0.51, 1) | 0.36 (0.31, 0.4) |
| STRATHEGE (1 study) | Risk Score ≥1 | VTE | Chauleur et al., 2008[27] | 0 | 54 | 5 | 2626 | 0.00 (0, 0.43) | 0.98 (0.97, 0.98) |

Abbreviations: CI, confidence interval; FN, false negative; FP, false positive; NR, not reported; RCOG, Royal College of Obstetricians and Gynaecologists; TN, true negative; TP, true positive; VTE, venous thromboembolism



For peer review only - http://bmjopen.bmj.com/site/about/guidelines.xhtml

# Table 4. Performance of RAMs applied postpartum to predict VTE

| Risk assessment models | Threshold or cut-off | Endpoint | Data source | Performance measures | | | | | |
|---|---|---|---|---|---|---|---|---|---|
| | | | | TP | FP | FN | TN | Sensitivity (95% CI) | Specificity (95% CI) |
| **Predicting postpartum VTE following vaginal and caesarean delivery** | | | | | | | | | |
| ACCP (1 study) | NR | VTE | Gassmann et al., 2020[30] | 0 | 34 | 0 | 310 | unable to estimate - no VTE | 0.90 (0.86, 0.93) |
| ACOG (1 study) | NR | VTE | Gassmann et al., 2020[30] | 0 | 30 | 0 | 314 | unable to estimate - no VTE | 0.91 (0.88, 0.94) |
| ASH (1 study) | NR | VTE | Gassmann et al., 2020[30] | 0 | 0 | 0 | 344 | unable to estimate - no VTE | 1.00 (0.99, 1) |
| Caprini (1 study) | Risk Score ≥2 | VTE | Tran et al., 2019[37] | 3 | 5780 | 0 | 311 | 1.00 (0.44, 1) | 0.05 (0.05, 0.06) |
| Caprini | Risk Score ≥3 | VTE | Tran et al., 2019[37] | 1 | 3066 | 2 | 3025 | 0.33 (0.06, 0.79) | 0.50 (0.48, 0.51) |
| Caprini | Risk Score ≥4 | VTE | Tran et al., 2019[37] | 0 | 1257 | 3 | 4834 | 0.00 (0, 0.56) | 0.79 (0.78, 0.80) |
| Padua (1 study) | Risk Score ≥4 | VTE | Tran et al., 2019[37] | 0 | 50 | 3 | 6041 | 0.00 (0, 0.56) | 0.99 (0.99, 0.99) |
| RCOG (3 studies) | NR | VTE | Gassmann et al., 2020[30] | 0 | 138 | 0 | 206 | unable to estimate - no VTE | 0.60 (0.55, 0.65) |
| RCOG | Risk Score ≥2 | VTE | Tran et al., 2019[37] | 1 | 3837 | 2 | 2254 | 0.33 (0.06, 0.79) | 0.37 (0.36, 0.38) |
| RCOG | ≥2 low risk factors or 1 high risk factor | VTE | Sultan et al., 2016[35] | 197 | 149205 | 115 | 283836 | 0.63 (0.58, 0.68) | 0.66 (0.65, 0.66) |
| SFOG (2 studies) | Risk Score ≥2 | VTE | Lindqvist et al., 2008 | 18 | 111 | 19 | 2273 | 0.49 (0.33, 0.64) | 0.95 (0.94, 0.96) |
| SFOG | ≥2 risk factors | VTE | Sultan et al., 2016[35] | 109 | 41145 | 412 | 620721 | 0.21 (0.18, 0.25) | 0.94 (0.94, 0.94) |
| Chau, 2019 (1 study[a]) | Risk Score ≥3 (2012 data set) | VTE | Chau et al., 2019[26] | 0 | 101 | 1 | 456 | 0.00 (0, 0.79) | 0.82 (0.78, 0.85) |
| Chau, 2019 | Risk Score ≥3 (2015 data set) | VTE | Chau et al., 2019[26] | 0 | 113 | 1 | 393 | 0.00 (0, 0.79) | 0.78 (0.74, 0.81) |
| Ellis-Kahana, 2020 (full model) (1 study[b]) | Risk Score >3 (high risk) | VTE | Ellis-Kahana 2020[39] | 68 | 7942 | 41 | 75449 | 0.62 (0.53, 0.71) | 0.90 (0.90, 0.91) |
| Ellis-Kahana, 2020 (without antepartum thromboembolic disorder) | Risk Score >3 (high risk) | VTE | Ellis-Kahana 2020[39] | 63 | 9926 | 46 | 73465 | 0.58 (0.48, 0.67) | 0.88 (0.88, 0.88) |
| Sultan, 2016 (1 study[c]) | ≥2 risk factors: top 35% (threshold: 7.2 per 10,000 deliveries) | VTE | Sultan et al., 2016[35] | 355 | 231480 | 166 | 430386 | 0.68 (0.64, 0.72) | 0.65 (0.65, 0.65) |
| Sultan, 2016 | ≥2 risk factors: top 25% (threshold: 8.7 per 10,000 deliveries) | VTE | Sultan et al., 2016[35] | 310 | 164976 | 211 | 496890 | 0.60 (0.55, 0.64) | 0.75 (0.75, 0.75) |
| Sultan, 2016 | ≥2 risk factors: top 20% (threshold: 9.8 per 10,000 deliveries) | VTE | Sultan et al., 2016[35] | 278 | 131921 | 243 | 529945 | 0.53 (0.49, 0.58) | 0.80 (0.80, 0.80) |
| Sultan, 2016 | ≥2 risk factors: top 10% (threshold: 14 per 10,000 deliveries) | VTE | Sultan et al., 2016[35] | 185 | 66053 | 336 | 595813 | 0.36 (0.32, 0.40) | 0.90 (0.90, 0.90) |
| Sultan, 2016 | ≥2 risk factors: top 6% (threshold: 18 per 10,000 deliveries) | VTE | Sultan et al., 2016[35] | 158 | 41096 | 363 | 620770 | 0.30 (0.27, 0.34) | 0.94 (0.94, 0.94) |
| Sultan, 2016 | ≥2 risk factors: top 5% (threshold: 19.7 per 10,000 deliveries) | VTE | Sultan et al., 2016[35] | 139 | 32980 | 382 | 628886 | 0.27 (0.23, 0.31) | 0.95 (0.95, 0.95) |
| Sultan, 2016 | ≥2 risk factors: top 1% (threshold: 41.2 per 10,000 deliveries) | VTE | Sultan et al., 2016[35] | 47 | 6576 | 474 | 655290 | 0.09 (0.07, 0.12) | 0.99 (0.99, 0.99) |
| **Predicting postpartum VTE following caesarean delivery only** | | | | | | | | | |
| ACOG (1 study) | Risk Score ≥3 | VTE | Lok et al., 2019[33] | 0 | 0 | 0 | 859 | unable to estimate - no VTE | 1.00 (1, 1) |



For peer review only - http://bmjopen.bmj.com/site/about/guidelines.xhtml

| | | | | | | | | | |
|---|---|---|---|---|---|---|---|---|---|
| RCOG (2 studies) | NR | VTE | Binstock & Larkin, 2019 (abstract)[24] | 11 | 2692 | 0 | 172 | 1.00 (0.74, 1) | 0.06 (0.05, 0.07) |
| RCOG | Risk Score ≥3 | VTE | Lok et al., 2019[33] | 0 | 649 | 0 | 210 | unable to estimate - no VTE | 0.24 (0.22, 0.27) |
| Binstock, 2019 (1 study) | NR | VTE | Binstock & Larkin, 2019 (abstract)[24] | 11 | 2635 | 0 | 229 | 1.00 (0.74, 1) | 0.08 (0.07, 0.09) |
| Cavazza 2012 (1 study) | Moderate/high/very high | VTE | Cavazza et al., 2012[25] | 0 | 268 | 1 | 232 | 0.00 (0, 0.79) | 0.46 (0.42, 0.51) |
| Lok, 2019 (1 study) | Risk Score ≥3 | VTE | Lok et al., 2019[33] | 0 | 28 | 0 | 831 | unable to estimate - no VTE | 0.97 (0.95, 0.98) |

Abbreviations: ACCP, American College of Chest Physicians; ACOG, American College of Obstetricians and Gynaecologists; ASH, American Society of Hematology; CI, confidence interval; FN, false negative; FP, false positive; NR, not reported; RCOG, Royal College of Obstetricians and Gynaecologists; SFOG, Swedish Society of Obstetrics and Gynecology; TN, true negative; TP, true positive; VTE, venous thromboembolism

[a] Data discrepancy in paper – text states analysis included 1069 women: 557 in the 2012 time frame and 512 in the 2015 time frame; however, data in tables suggest 558 women included in the 2012 time frame and 507 in the 2015 time frame.
[b] Internal-validation study. Full risk prediction model: C-statistic, 0.817 (95% CI: 0.768, 0.865) with Hosmer-Lemeshow P-value = 0.297; Model without antepartum thromboembolic disorder: C-statistic, 0.778 (95% CI: 0.729, 0.826) with Hosmer-Lemeshow P-value = 0.114
[c] Sultan et al.,[35] final risk prediction model in external Swedish cohort: C-statistic, 0.73 (95% CI: 0.71 to 0.75) and calibration slope, 1.11 (95% CI: 1.01 to 1.20)

For peer review only

## DISCUSSION

### Summary of results

This systematic review identified 19 externally validated RAMs (and one internally validated risk model) that aimed to predict the risk of VTE in pregnant and postpartum women and who could be selected for thromboprophylaxis. Although various risk models (based on a variety of predictor variables) are being used, most of these lacked rigorous development and evaluation. The predictive accuracy of the RAMs was highly variable, and the substantial risk of bias concerns and the general lack of methodological clarity and unclear applicability make meaningful comparisons of the evidence difficult.

### Interpretation of results

Despite the development and use of various RAMs to predict the risk of developing VTE in women who are pregnant or in the puerperium (within 6 weeks post-delivery), VTE remains the leading cause of direct maternity mortality in the UK (MBRRACE-UK report 2021). Several explanations for this are possible: the risk assessment tools are inadequate; the application of these tools is incomplete or inaccurate; the underlying VTE risks of the pregnant population (increasing age, BMI and comorbidities) are changing from when the RAMS were developed; or all three problems are operating.

The use of thromboprophylaxis was reported in nine studies[23, 25, 28-31, 33, 35, 36] (ranging from 3%[35] to 100%)[23, 28] This may lead to underestimation of predictive accuracy if a given RAM were to predict VTE events that were subsequently prevented by thromboprophylaxis. In the remaining studies (n=8) where thromboprophylaxis use was not reported (n=8), further analysis of its impact upon the performance of the RAMs was not possible. This also suggests that the degree to which thromboprophylaxis reduces the risk of VTE in those who received it



For peer review only - http://bmjopen.bmj.com/site/about/guidelines.xhtml

cannot be accurately estimated. Moreover, the lack of data on the predictive performance of weight-based LMWH dosing, dosage change throughout pregnancy and D-Dimer testing in the included studies also precluded further analysis of its association with VTE.

**Comparison to the existing literature**

To our knowledge, there are no previous systematic reviews on this topic. However, recently several large registries have been interrogated in an attempt to derive robust prediction rules for this population, albeit with some methodological concerns. Sultan et al.,[35] developed (using a large English-based registry database covering 6% of the population) and validated (using a Swedish national database registry) a risk prediction tool to estimate the absolute risk of VTE in postpartum women according to their individual risk factor combinations. Despite the low incidence of VTE in both cohorts (<0.08%), their model showed good discrimination in the external cohort and poor sensitivity at predicting those at risk of experiencing VTE. In addition, their model lacked some important VTE risk factors (e.g. thrombophilia, antepartum immobilisation), and possibly underestimated the risks due to diagnosis limited to diagnostic coding (e.g. varicose veins, severity of comorbidities) and the use of thromboprophylaxis in both cohorts.[42] Ellis-Kahana et al.,[39] also derived (using a large national database from the US) a risk prediction model for VTE in obese pregnant women and indicated strong discrimination. However, this model still requires external validation.

**Strengths and limitations**

This systematic review has several strengths. It is the first systematic review to evaluate RAMs for predicting the risk of developing VTE in women during pregnant and the puerperium periods, and was conducted with robust methodology in accordance with the PRISMA statement[15] and the protocol was registered with the PROSPERO register. Clinical



For peer review only - http://bmjopen.bmj.com/site/about/guidelines.xhtml

experts, in addition to the core review team, were involved and consulted throughout as advisors and to assess the validity and applicability of research findings during the review processes.

The main limitations of this study related to the observational nature of the studies reviewed and their own limitations. Most of the included risk prediction studies were retrospective cohorts. Retrospective cohort studies of large health database registries are limited by poor data quality and failure to accurately ascertain outcomes and case–control designs are prone to bias including uncontrolled confounding, temporal and selection bias.[43] Conversely, better quality data may be obtained with prospective cohorts, but smaller sample sizes will lack statistical power. In addition, most of the external validation studies evaluated predictive performance of risk models that were not statistically derived (i.e. without model development and internal validation). This process is vital, as risk models with only external validation may be subject to overfitting and optimism.[40] Similarly, the absence of model performance measures such as calibration or discrimination hinders the full appraisal of models.[41]

Due to the high levels of heterogeneity between studies, we were unable to undertake any meta-analysis or statistical examination of the causes of heterogeneity due to the small number of external validation studies per risk model. Potential sources of heterogeneity include variation in study design, the study population, risk model implementation, outcome definition and measurement, and the use of thromboprophylaxis. As a result, we reported descriptive statistics to provide a better understanding of the evidence base applicable to the subject matter, and shortcomings regarding reliability and validity of the data. Finally, assessments on study relevance, information gathering and validity of articles were unblinded



For peer review only - http://bmjopen.bmj.com/site/about/guidelines.xhtml

and could potentially have been influenced by pre-formed opinions. However, masking is resource intensive with uncertain benefits in protecting against bias decisions.[44]

**Implications for policy, practice and future research**

VTE risk assessment is challenging for numerous reasons. Many risk factors for VTE are pre-existing and non-modifiable (such as parity and inherited thrombophilia). These are then often combined with evolving risk factors which can change over the course of a pregnancy or postnatal period. Despite wide scale awareness of VTE being a major contributor to maternal mortality, numerous challenges with VTE risk stratification have been highlighted. In the UK, the MBRRACE-UK report (Saving Lives, Improving Mothers' Care 2018)[45] shows that doctors and midwives find existing risk scoring systems difficult to apply consistently in clinical practice. There is a need for development of a RAM that is simpler and more reproducible. NICE Guidelines on the use of thromboprophylaxis (NG89)[46] concluded that the tool described by Sultan et al.[35] showed poor sensitivity compared to their pre-specified target of 90% sensitivity. However, this high level of sensitivity may not be realistic because there is evidence that only 70% of women having antenatal pulmonary embolism had any identifiable classic risk factors suggesting that sensitivity rates above 70% may not be achievable.[47] In addition, a high sensitivity rate is usually associated with a lower specificity rate and the overall balance of benefits and harms may be undesirable if that means exposing a high proportion of women to thromboprophylaxis.

Despite lack of evidence, many guidelines and clinical care bundles include the use of RAMs to guide VTE prophylaxis. Recently published ACOG guidelines state that the most RAMs have not been validated prospectively in the obstetric population and that current usage of such models is based on extrapolations from non-pregnant women, who differ biologically



For peer review only - http://bmjopen.bmj.com/site/about/guidelines.xhtml

from pregnant women. The practice bulletin emphasises the need for more research to identify optimal models.[37] Although further research is clearly needed the routine use of thromboprophylaxis may present a barrier to generating accurate and precise estimates of the prognostic accuracy of RAMs. Further work to improve RAMs to help stratify the risk of VTE in women who are pregnant or in the puerperium could focus on using decision-analytic modelling to compare the effects, harms and costs of giving thromboprophylaxis to patients with varying risk of VTE. This would allow determination of the risk threshold at which thromboprophylaxis provides optimal overall benefit. Subsequent work to validate these findings would require primary research. Despite the limitations of undertaking accuracy studies in populations where thromboprophylaxis is routinely used, future research could focus on selected higher risk groups who are more likely to benefit from prophylaxis and, with a higher prevalence of VTE, are more amenable to an appropriately powered prospective study. However, given the uncertain benefits and harms of VTE thromboprophylaxis during pregnancy and the postpartum period,[14, 48] risk prediction studies should be undertaken alongside (or as a part of) randomised trials of prophylaxis in targeted groups deemed to be at higher risk of VTE.

## CONCLUSIONS

Currently there are a number of risk assessment models for assessing risk of VTE in pregnancy and the puerperium. Our review has shown that none of these models has been adequately validated and they have limited abilities to detect those at risk of VTE.

For peer review only



For peer review only - http://bmjopen.bmj.com/site/about/guidelines.xhtml

## ACKNOWLEDGEMENTS

The authors would like to thank all additional members of the core project group for NIHR131021 for input and commentary throughout the work. We are also indebted to Donna Davis for assistance with logistics and administration.

## COMPETING INTEREST

CNP reports personal fees from Sanofi, and is the lead developer of the RCOG Green Top Guideline on thromboprophylaxis in pregnancy (37a). All other authors declare no competing interests.

## SOURCE OF FUNDING

This study was funded by the United Kingdom National Institute for Health Research Health Technology Assessment Programme (project number 131021). The views expressed in this paper are those of the authors and not necessarily those of the NIHR HTA Programme. Any errors are the responsibility of the authors. The funders had no role in the study design, in the collection, analysis and interpretation of data; in the writing of the manuscript; and in the decision to submit the manuscript for publication.

## DETAILS OF ETHICS APPROVAL

Not applicable, as this is a systematic review of published papers.

## DATA SHARING STATEMENT

All data relevant to the study are included in the article or uploaded as supplementary information



For peer review only - http://bmjopen.bmj.com/site/about/guidelines.xhtml

## AUTHOR CONTRIBUTIONS

AP co-ordinated the review. SD, AP, JD, JH, MC, BH, CNP, and SG were responsible for conception, design and obtaining funding for the study. MC developed the search strategy, undertook searches, and organised retrieval of papers. AP, GR, JH, BH, JD, CNP and SG were responsible for the acquisition, analysis and interpretation of data. BH, JD, CNP and SG helped interpret and provided a methodological, policy and clinical perspective on the data. AP, BH, JD and SD were responsible for the drafting of this paper, although all authors provided comments on the drafts, read and approved the final version. AP is the guarantor for the paper.

## COPYRIGHT LICENCE STATEMENT

The project was funded by the United Kingdom National Institute for Health Research Health Technology Assessment Programme and as such is covered by Crown copyright. The Corresponding Author has the right to grant on behalf of all authors and does grant on behalf of all authors, **a non-exclusive license** on a worldwide basis to BMJ publishing (BMJ) and its Licensees to permit this article (if accepted) to be published by BMJ , as set out in our licence.

For peer review only

## REFERENCES

1. Say L, Chou D, Gemmill A, et al. Global causes of maternal death: a WHO systematic analysis. *Lancet Glob Health*. 2014;2(6):e323-3.

2. Heit JA, Kobbervig CE, James AH, Petterson TM, Bailey KR, Melton LJ, 3rd. Trends in the incidence of venous thromboembolism during pregnancy or postpartum: a 30-year population-based study. *Ann of Intern Med*. 2005;143(10):697-706.

3. James AH, Jamison MG, Brancazio LR, Myers ER. Venous thromboembolism during pregnancy and the postpartum period: incidence, risk factors, and mortality. *Am J Obstet Gynecol.* 2006;194(5):1311-5.

4. Lindqvist P, Dahlbäck B, Marŝál K. Thrombotic risk during pregnancy: a population study. *Obstet Gynecol.* 1999;94(4):595-599.

5. Royal College of Obstetricians & Gynaecologists. Thrombosis and Embolism during Pregnancy and the Puerperium, Reducing the Risk (Green-top Guideline No. 37a). 2015 [internet] [https://www.rcog.org.uk/en/guidelines-research-services/guidelines/gtg37a/]. Accessed 4 April 2022.

6. Pomp ER, Lenselink AM, Rosendaal FR, Doggen CJ. Pregnancy, the postpartum period and prothrombotic defects: risk of venous thrombosis in the MEGA study. *J Thromb and Haemost*. 2008;6(4):632–637.

7. Bates SM, Rajasekhar A, Middeldorp S, McLintock C, Rodger MA, James AH, et al. American Society of Hematology 2018 guidelines for management of venous thromboembolism: venous thromboembolism in the context of pregnancy. *Blood Adv.* 2018;2(22):3317-59.

8. Bates SM, Greer IA, Middeldorp S, Veenstra DL, Prabulos A-M, Vandvik PO. VTE, thrombophilia, antithrombotic therapy, and pregnancy: Antithrombotic Therapy and



For peer review only - http://bmjopen.bmj.com/site/about/guidelines.xhtml

Prevention of Thrombosis, 9th ed: American College of Chest Physicians Evidence-Based Clinical Practice Guidelines. *Chest.* 2012;141(2 Suppl):e691S-e736S.

9. Chan W-S, Rey E, Kent NE, et al. Venous thromboembolism and antithrombotic therapy in pregnancy. *J Obstet Gynaecol Can*. 2014;36(6):527-53.

10. D'Alton ME, Friedman AM, Smiley RM, et al. National Partnership for Maternal Safety: Consensus Bundle on Venous Thromboembolism. *Obstet Gynecol*. 2016;128(4):688-698.

11. James A, Birsner M, Kaimal A, in collaboration with American College of Obstetricians and Gynecologists' Committee on Practice Bulletins-Obstetrics. ACOG Practice Bulletin No. 196. Summary: thromboembolism in pregnancy. *Obstet Gynecol*. 2018;132(1):243-248.

12. Lindqvist PG, Hellgren M. Obstetric thromboprophylaxis: the Swedish guidelines. *Adv Hematol*. 2011;2011:157483.

13. McLintock C, Brighton T, Chunilal S, et al. Recommendations for the prevention of pregnancy-associated venous thromboembolism. *Aust N Z J Obstet Gynaecol*. 2012;52(1):3-13.

14. Middleton P, Shepherd E, Gomersall JC. Venous thromboembolism prophylaxis for women at risk during pregnancy and the early postnatal period. Cochrane Database Syst Rev. 2021;3(3):CD001689.

15. Moher D, Liberati A, Tetzlaff J, Altman DG, PRISMA Group, . Preferred reporting items for systematic reviews and meta-analyses: the PRISMA statement. *Ann Intern Med.* 2009;151(4):264-9.

16. Davis S, Goodacre S, Pandor A, et al. Estimating the value of future research to improve guidelines on thromboprophylaxis for women during pregnancy and after delivery.



For peer review only - http://bmjopen.bmj.com/site/about/guidelines.xhtml

2021 [Internet] [https://fundingawards.nihr.ac.uk/award/NIHR131021]. Accessed 4 April 2022.

17. Wilczynski N. McMaster University – HiRU's Approach to Search Filter Development. [Internet] [https://hiru.mcmaster.ca/hiru/HIRU_Hedges_home.aspx]. Accessed 4 April 2022.

18. Moons KGM, Wolff RF, Riley RD, et al. PROBAST: A Tool to Assess Risk of Bias and Applicability of Prediction Model Studies: Explanation and Elaboration. *Ann Intern Med* 2019;170(1):W1-W33.

19. Wolff RF, Moons KGM, Riley RD, et al. PROBAST: A Tool to Assess the Risk of Bias and Applicability of Prediction Model Studies. *Ann Intern Med.* 2019;170(1):51-8.

20. Centre for Reviews and Dissemination. Systematic Reviews: CRD's Guidance for Undertaking Reviews in Health Care: Centre for Reviews and Dissemination, York; 2009.

21. McKenzie JE, Brennan SE, Ryan RE, Thomson HJ, Johnston RV. Chapter 9: Summarizing study characteristics and preparing for synthesis. In: Higgins JPT, Thomas J, Chandler J, Cumpston M, Li T, Page MJ, et al., editors. Cochrane Handbook for Systematic Reviews of Interventions version 6.3 (updated February 2022): Cochrane; 2022.

22. Hosmer DW, Lemeshow S. Applied Logistic Regression (2nd Edition): John Wiley & Sons. New York; 2000.

23. Bauersachs RM, Dudenhausen J, Faridi A, et al. Risk stratification and heparin prophylaxis to prevent venous thromboembolism in pregnant women. *Thromb Haemost*. 2007;98(6):1237-1245.

24. Binstock AB, Larkin JC. Development of a Venous Thromboembolism Prevention Tool in the Postpartum Period. *Obstet Gynecol*. 2019;133(SUPPL 1).



For peer review only - http://bmjopen.bmj.com/site/about/guidelines.xhtml

25. Cavazza S, Rainaldi MP, Adduci A, Palareti G. Thromboprophylaxis following cesarean delivery: one site prospective pilot study to evaluate the application of a risk score model. *Thromb Res*. 2012;129(1):28-31.

26. Chau C, Campagna J, Vial M, Rambeaud C, Loundou A, Bretelle F. Use of a personalized iterative score to evaluate risk of venous thromboembolism during pregnancy and puerperium. *Int J Gynaecol Obstet*. 2019;144(3):277-282.

27. Chauleur C, Quenet S, Varlet M-N, et al. Feasibility of an easy-to-use risk score in the prevention of venous thromboembolism and placental vascular complications in pregnant women: a prospective cohort of 2736 women. *Thromb Res*. 2008;122(4):478-484.

28. Dargaud Y, Rugeri L, Fleury C, et al. Personalized thromboprophylaxis using a risk score for the management of pregnancies with high risk of thrombosis: a prospective clinical study. *J Thromb Haemost*. 2017;15(5):897-906.

29. Dargaud Y, Rugeri L, Ninet J, Negrier C, Trzeciak MC. Management of pregnant women with increased risk of venous thrombosis. *Int J Gynaecol Obstet*. 2005;90(3):203-207.

30. Gassmann N, Viviano M, Righini M, Fontana P, Martinez De Tejada B, Blondon M. Estimating the risk thresholds used by guidelines to recommend postpartum thromboprophylaxis. *J Thromb Haemost*. Epub 2020.

31. Hase EA, Barros VIPVLd, Igai AMK, Francisco RPV, Zugaib M. Risk assessment of venous thromboembolism and thromboprophylaxis in pregnant women hospitalized with cancer: Preliminary results from a risk score. *Clinics (Sao Paulo)*. 2018;73:e368.

32. Lindqvist PG, Torsson J, Almqvist A, Bjorgell O. Postpartum thromboembolism: severe events might be preventable using a new risk score model. *Vasc Health Risk Manag*. 2008;4(5):1081-1087.



For peer review only - http://bmjopen.bmj.com/site/about/guidelines.xhtml

33. Lok WY, Kong CW, To WWK. A local risk score model for venous thromboembolism prophylaxis for caesarean section in Chinese women and comparison with international guidelines. *Taiwan J Obstet Gynecol.* 2019;58(4):520-525.

34. Shacaluga A, Rayment R. Venous thromboembolic risk assessment in pregnancy: comparison of the All-Wales maternity risk assessment tool with guidance from the Royal College of Obstetrics and Gynaecology. *Br J Haematol*. 2019;185(1):162-165.

35. Sultan AA, West J, Grainge MJ, et al. Development and validation of risk prediction model for venous thromboembolism in postpartum women: multinational cohort study. *BMJ.* 2016;355:i6253.

36. Testa S, Passamonti SM, Paoletti O, et al. The "Pregnancy Health-care Program" for the prevention of venous thromboembolism in pregnancy. *Intern Emerg Med*. 2015;10(2):129-134.

37. Tran JP, Stribling SS, Ibezim UC, et al. Performance of Risk Assessment Models for Peripartum Thromboprophylaxis. *Reprod Sci*. 2019;26(9):1243-1248.

38. Weiss N, Bernstein PS. Risk factor scoring for predicting venous thromboembolism in obstetric patients. *Am J Obstet Gynecol*. 2000;182(5):1073-5.

39. Ellis-Kahana J, Sparks AD, Gimovsky AC, James AH, Ahmadzia HK. Developing a model for predicting venous thromboembolism in obese pregnant women in a national study. *Thromb Res*. 2020;191:42-49.

40. Collins GS, Reitsma JB, Altman DG, Moons KG. Transparent reporting of a multivariable prediction model for individual prognosis or diagnosis (TRIPOD): the TRIPOD Statement. *BMC Med.* 2015;13:1.

41. Moons KG, de Groot JA, Bouwmeester W, et al. Critical appraisal and data extraction for the systematic reviews of prediction modelling studies: the CHARMS checklist. *PLoS Med.* 2014;11:e1001744.



For peer review only - http://bmjopen.bmj.com/site/about/guidelines.xhtml

42. Andrew L, Ni Ainle F, Blondon M, Rodger MA, Skeith L. Preventing postpartum venous thromboembolism: A call to action to reduce undue maternal morbidity and mortality. *Thromb Res*. 2020;193:190-7.

43. Reps JM, Ryan PB, Rijnbeek PR, Schuemie MJ. Design matters in patient-level prediction: evaluation of a cohort vs. case-control design when developing predictive models in observational healthcare datasets. *J Big Data*. 2021;8(108).

44. Higgins JPT, Thomas J, Chandler J, Cumpston M, Li T, Page MJ, et al. Cochrane Handbook for Systematic Reviews of Interventions version 6.3 (updated February 2022). Cochrane 2022.

45. Knight M, Bunch K, Tuffnell D, Jayakody H, Shakespeare J, Kotnis R, et al. Saving Lives, Improving Mothers' Care - Lessons learned to inform maternity care from the UK and Ireland Confidential Enquiries into Maternal Deaths and Morbidity 2014-16. National Perinatal Epidemiology Unit, University of Oxford, Oxford 2018.

46. National Institute for Health and Care Excellence. Venous thromboembolism in over 16s: reducing the risk of hospital-acquired deep vein thrombosis or pulmonary embolism - NICE Guideline 89. NICE; London UK.; 2019.

47. Knight M. Antenatal pulmonary embolism: Risk factors, management and outcomes. *BJOG*. 2008;115(4):453-461.

48. Lu MY, Blanchard CT, Ausbeck EB, et al. Evaluation of a risk-stratified, heparin-based, obstetric thromboprophylaxis protocol. *Obstet Gynecol*. 2021;138(4):530-8.

For peer review only



For peer review only - http://bmjopen.bmj.com/site/about/guidelines.xhtml

## FIGURE LEGENDS

**Figure 1** **Study flow chart (adapted)**

**Figure 2** **PROBAST assessment summary graph - Review authors' judgements**

For peer review only

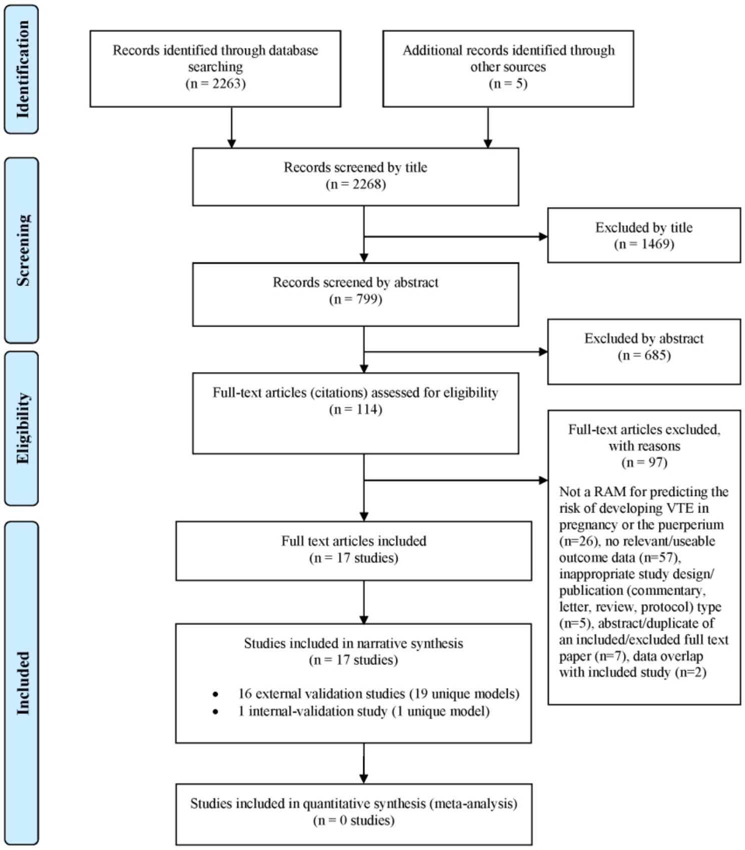


**Figure 1** **Study flow chart (adapted)**

Study flow chart (adapted)

210x297mm (300 x 300 DPI)

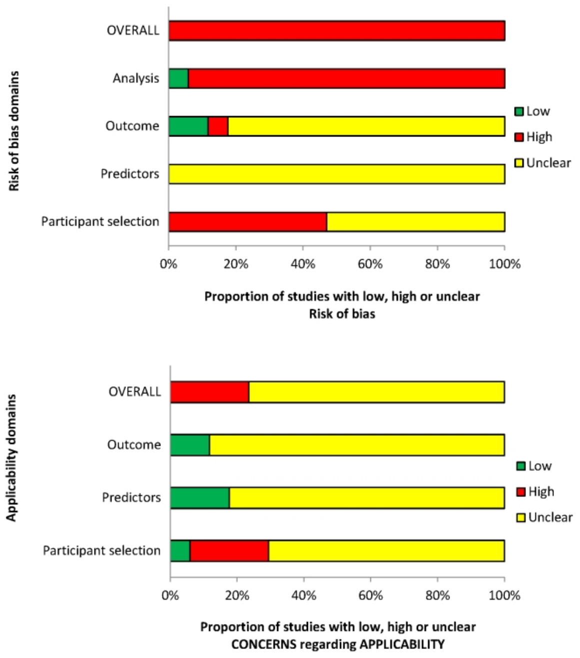


**Figure 2** **PROBAST assessment summary graph - Review authors' judgements**

PROBAST assessment summary graph - Review authors' judgements

210x297mm (300 x 300 DPI)

**APPENDICES**

**Pandor et al. Risk assessment models for venous thromboembolism in pregnancy and in the puerperium – a systematic review**

**Contents**


| | |
|---|---|
| Appendix S1 | Literature search strategies |
| Appendix S2 | List of excluded studies with rationale |
| Appendix S3 | Simplified summary of widely evaluated generic RAMs, their associated characteristics and composite clinical variables |


For peer review only

## APPENDIX S1 LITERATURE SEARCH STRATEGIES

| | |
|---|---|
| **Database searched:** | **Ovid MEDLINE(R) Epub Ahead of Print, In-Process & Other Non-Indexed Citations, Ovid MEDLINE(R) Daily, Ovid MEDLINE and Versions(R)** |
| **Platform or provider used:** | **Ovid SP** |
| **Date of coverage:** | **1946 to February 2021** |
| **Search undertaken:** | **February 2021** |

For peer review only

1 Pregnant Women/ or exp Pregnancy Complications/ or exp Maternal Health Services/ or exp Fetal Monitoring/ or exp Prenatal Diagnosis/ or Perinatal Care/ or Labor pain/ or Analgesia, Obstetric/ or exp Obstetric Surgical Procedures/ or exp Postpartum Period/
2 (pregnan* or antenatal* or ante-natal* or prenatal* or pre-natal* or gestational* or matern* or perinatal* or peri-natal* or postnatal* or post-natal* or postpartum or post-partum or puerper* or obstetric).mp.
3 1 or 2
4 pulmonary embolism/ or thromboembolism/ or venous thromboembolism/ or venous thrombosis/ or upper extremity deep vein thrombosis/
5 (((venous or vein) adj (thrombosis or thromboses or thrombus or thromboemboli*)) or (dvt or vte) or ((pulmonary or lung) adj3 (embolism or emboli or embolus or emboliz* or thromboemboli*))).ti,ab.
6 4 or 5
7 editorial/ or news/ or exp historical article/ or anecdotes as topic/ or comment/ or case report/ or (letter or comment).ti.
8 randomized controlled trial/ or random*.ti,ab.
9 7 not 8
10 animals/ not humans/
11 exp animals, laboratory/
12 exp animal experimentation/
13 exp models, animal/
14 exp rodentia/
15 (rat or rats or mouse or mice).ti.
16 9 or 10 or 11 or 12 or 13 or 14 or 15
17 6 not 16
18 (risk* adj2 assess*).ti,ab.
19 ((score* or scoring) adj2 (tool* or system*)).ti,ab.
20 ((risk* or predict* or prognos*) adj4 (tool* or rule* or index* or indices or score* or scoring or scale* or model* or system* or algorithm* or stratif* or criteria or calculat*)).ti,ab.
21 department of health.ti,ab,au.
22 (guidance or guideline*).ti,hw,pt.
23 18 or 19 or 20 or 21 or 22
24 17 and 23
25 3 and 24

| | |
|---|---|
| **Databases searched:** | **EMBASE** |
| **Platform or provider used:** | **Ovid SP** |
| **Date of coverage:** | **1974 to February 2021** |
| **Search undertaken:** | **February 2021** |

1 exp pregnancy/ or maternal health service/ or exp pregnancy complication/ or exp fetus monitoring/ or exp prenatal diagnosis/ or exp perinatal care/ or exp obstetric analgesia/ or exp labor pain/ or exp obstetrics/ or obstetric analgesia/ or exp obstetric operation/ or puerperium/
2 (pregnan* or antenatal* or ante-natal* or prenatal* or pre-natal* or gestational* or matern* or perinatal* or peri-natal* or postnatal* or post-natal* or postpartum or post-partum or puerper* or obstetric or labo?r).mp.
3 1 or 2
4 lung embolism/ or exp venous thromboembolism/ or exp vein thrombosis/ or upper extremity deep vein thrombosis/
5 (((venous or vein) adj (thrombosis or thromboses or thrombus or thromboemboli*)) or (dvt or vte) or ((pulmonary or lung) adj3 (embolism or emboli or embolus or emboliz* or thromboemboli*))).ti,ab.
6 4 or 5
7 editorial/ or comment/ or case report/ or (letter or comment).ti.
8 randomized controlled trial/ or random*.ti,ab.
9 7 not 8
10 exp animal/ not exp human/
11 (rat or rats or mouse or mice).ti.
12 9 or 10
13 6 not 12
14 (risk* adj2 assess*).ti,ab.
15 ((score* or scoring) adj2 (tool* or system*)).ti,ab.
16 ((risk* or predict* or prognos*) adj4 (tool* or rule* or index* or indices or score* or scoring or scale* or model* or system* or algorithm* or stratif* or criteria or calculat*)).ti,ab.
17 department of health.ti,ab,au.
18 (guidance or guideline*).ti,hw,pt.
19 14 or 15 or 16 or 17 or 18
20 13 and 19
21 3 and 20

For peer review only

**Databases searched:** **Cochrane CENTRAL Register of Randomised Controlled Trials & Cochrane Database of Systematic Reviews**

**Platform or provider used:** **www.thecochranelibrary.com**

**Date of coverage:** **Inception to February 2021**

**Search undertaken:** **February 2021**

#1 MeSH descriptor: [Pregnancy] explode all trees
#2 MeSH descriptor: [Pregnancy Complications] 1 tree(s) exploded
#3 MeSH descriptor: [Maternal Health Services] explode all trees
#4 MeSH descriptor: [Fetal Monitoring] explode all trees
#5 MeSH descriptor: [Perinatal Care] explode all trees
#6 MeSH descriptor: [Labor Pain] explode all trees
#7 MeSH descriptor: [Analgesia, Obstetrical] explode all trees
#8 MeSH descriptor: [Obstetric Surgical Procedures] explode all trees
#9 MeSH descriptor: [Postpartum Period] explode all trees
#10 (pregnan* or antenatal* or "ante-natal*" or prenatal* or "pre-natal*" or gestational* or matern* or perinatal* or "peri-natal*" or postnatal* or "post-natal*" or postpartum or "post-partum" or puerper* or obstetric):ti,ab,kw (Word variations have been searched)
#11 MeSH descriptor: [Pulmonary Embolism] explode all trees
#12 MeSH descriptor: [Venous Thromboembolism] explode all trees
#13 MeSH descriptor: [Venous Thrombosis] explode all trees
#14 MeSH descriptor: [Upper Extremity Deep Vein Thrombosis] explode all trees
#15 ((venous or vein) near/2 (thrombosis or thromboses or thrombus or thromboemboli*)):ti,ab,kw OR ((dvt or vte)):ti,ab,kw OR ((pulmonary or lung) near/2 (embolism or emboli or embolus or emboliz* or thromboemboli*)):ti,ab,kw (Word variations have been searched)
#16 #1 or #2 or #3 or #4 or #5 or #6 or #7 or #8 or #9 or #10
#17 #11 or #12 or #13 or #14 or #15
#18 #16 and #17
#19 (risk* or predict* or prognos*):ti,ab,kw AND (tool* or rule* or index* or indices or score* or scoring or scale* or model* or system* or algorithm* or stratif* or criteria or calculat*):ti,ab,kw OR ((pulmonary or lung) near/3 (embolism or emboli or embolus or emboliz* or thromboemboli*)):ti,ab,kw (Word variations have been searched)
#20 (score* or scoring) near/2 (tool* or system*)
#21 guidance or guideline* or "department of health"
#22 #19 or #20 or #21
#23 #18 and #22

For peer review only

**APPENDIX S2 LIST OF EXCLUDED STUDIES WITH RATIONALE**

| | Authors, year | Reason for exclusion |
|---|---|---|
| 1. | Abdul Sultan et al., 2013 [1] | Not a RAM for predicting the risk of developing VTE in pregnancy or the puerperium |
| 2. | Ahmadzia et al., 2019 [2] | Abstract of included full text study (Ellis-Kahana 2020) |
| 3. | Alsayegh et al., 2016 [3] | No relevant/useable outcome data |
| 4. | Bahl et al., 2010 [4] | Not a RAM for predicting the risk of developing VTE in pregnancy or the puerperium |
| 5. | Banfield et al., 2013 [5] | No relevant/useable outcome data |
| 6. | Bare et al., 2013 [6] | Not a RAM for predicting the risk of developing VTE in pregnancy or the puerperium |
| 7. | Barros et al., 2017 [7] | No relevant/useable outcome data |
| 8. | Barros et al., 2017 [8] | No relevant/useable outcome data |
| 9. | Barros et al., 2020 [9] | No relevant/useable outcome data |
| 10. | Barros et al., 2011 [10] | No relevant/useable outcome data |
| 11. | Bastek et al., 2011 [11] | Not a RAM for predicting the risk of developing VTE in pregnancy or the puerperium |
| 12. | Beckett et al., 2013 [12] | No relevant/useable outcome data |
| 13. | Berkin et al., 2016 [13] | No relevant/useable outcome data |
| 14. | Blondon et al., 2015 [14] | Not a RAM for predicting the risk of developing VTE in pregnancy or the puerperium |
| 15. | Blondon and Hugon-Rodin 2017 [15] | Commentary |
| 16. | Campbell 2013 [16] | No relevant/useable outcome data |
| 17. | Cavazza et al., 2010 [17] | Abstract of included full text study (Cavazza 2012) |
| 18. | Chauleur et al., 2017 [18] | Data overlap - patients included in Chauleur 2018 (included study) |
| 19. | Chauleur et al., 2010 [19] | No relevant/useable outcome data |
| 20. | Chauleur et al., 2018 [20] | No relevant/useable outcome data |
| 21. | Cooley et al., 2016 [21] | No relevant/useable outcome data |
| 22. | Creagh et al., 2014 [22] | Not a RAM for predicting the risk of developing VTE in pregnancy or the puerperium |
| 23. | Creagh et al., 2013 [23] | Not a RAM for predicting the risk of developing VTE in pregnancy or the puerperium |
| 24. | Crowley et al., 2013 [24] | Abstract of excluded full text study (Crowley 2017) |
| 25. | Crowley et al., 2017 [25] | No relevant/useable outcome data |
| 26. | Crowley et al., 2013 [26] | Abstract of excluded full text study (Crowley 2017) |
| 27. | Cunningham et al., 2015 [27] | Not a RAM for predicting the risk of developing VTE in pregnancy or the puerperium |

For peer review only

| | | |
|---|---|---|
| 28. | Cutts et al., 2014 [28] | Not a RAM for predicting the risk of developing VTE in pregnancy or the puerperium |
| 29. | Cutts et al., 2011 [29] | Not a RAM for predicting the risk of developing VTE in pregnancy or the puerperium |
| 30. | Dargaud et al., 2015 [30] | Abstract of included full text study (Dargaud 2017) |
| 31. | Dargaud et al., 2009 [31] | Data overlap - patients included in Dargaud 2017 (included study) |
| 32. | Dargaud et al., 2009 [32] | No relevant/useable outcome data |
| 33. | Davis and Hadpawat-Lee 2017 [33] | No relevant/useable outcome data |
| 34. | Dentali et al., 2020 [34] | Not a RAM for predicting the risk of developing VTE in pregnancy or the puerperium |
| 35. | Francis Kim et al., 2020 [35] | No relevant/useable outcome data |
| 36. | Francis Kim et al., 2020 [36] | No relevant/useable outcome data |
| 37. | Fuller et al., 2018 [37] | Not a RAM for predicting the risk of developing VTE in pregnancy or the puerperium |
| 38. | Gassmann et al., 2020 [38] | Abstract of included full text study (Gassmann 2020) |
| 39. | Gerhardt et al., 2016 [39] | Not a RAM for predicting the risk of developing VTE in pregnancy or the puerperium |
| 40. | Gherghe et al., 2012 [40] | No relevant/useable outcome data |
| 41. | Goffman et al., 2009 [41] | No relevant/useable outcome data |
| 42. | Gomez et al., 2020 [42] | No relevant/useable outcome data |
| 43. | Grille et al., 2015 [43] | No relevant/useable outcome data |
| 44. | Goodfellow et al., 2017 [44] | No relevant/useable outcome data |
| 45. | Grant et al., 2016 [45] | No relevant/useable outcome data |
| 46. | Handa et al., 2015 [46] | No relevant/useable outcome data |
| 47. | Harris et al., 2016 [47] | No relevant/useable outcome data |
| 48. | Hayes-Ryan and Byrne 2011 [48] | No relevant/useable outcome data |
| 49. | Hayes-Ryan and Byrne 2012 [49] | No relevant/useable outcome data |
| 50. | Heath and Goodfellow 2016 [50] | No relevant/useable outcome data |
| 51. | Henke and Pannucci 2010 [51] | Review |
| 52. | Kazi et al., 2020 [52] | Not a RAM for predicting the risk of developing VTE in pregnancy or the puerperium |
| 53. | Lacoss and Jheeta 2017 [53] | Not a RAM for predicting the risk of developing VTE in pregnancy or the puerperium |
| 54. | Li et al., 2018 [54] | Not a RAM for predicting the risk of developing VTE in pregnancy or the puerperium |
| 55. | Lindqvist 2018 [55] | Letter |
| 56. | Lindqvist and Hellgren 2011 [56] | No relevant/useable outcome data |
| 57. | Lindqvist et al., 2002 [57] | No relevant/useable outcome data |

For peer review only

| | | |
|---|---|---|
| 58. | Lou Mercade et al., 2017 [58] | No relevant/useable outcome data |
| 59. | Marks and Maiti 2018 [59] | No relevant/useable outcome data |
| 60. | Mcarthur et al., 2011 [60] | No relevant/useable outcome data |
| 61. | Mpouzouki et al., 2013 [61] | No relevant useable outcome data |
| 62. | Naidoo et al., 2019 [62] | No relevant/useable outcome data |
| 63. | Nct 2018 [63] | Protocol |
| 64. | Noone et al., 2013 [64] | No relevant/useable outcome data |
| 65. | O'Connor et al., 2011 [65] | Not a RAM for predicting the risk of developing VTE in pregnancy or the puerperium |
| 66. | O'Keefe et al., 2019 [66] | Not a RAM for predicting the risk of developing VTE in pregnancy or the puerperium |
| 67. | Omunakwe et al., 2017 [67] | No relevant/useable outcome data |
| 68. | Orfanelli et al., 2017 [68] | No relevant/useable outcome data |
| 69. | O'Shaughnessy et al., 2017 [69] | No relevant/useable outcome data |
| 70. | O'Shaughnessy et al., 2018 [70] | No relevant/useable outcome data |
| 71. | O'Shaughnessy et al., 2019 [71] | No relevant/useable outcome data |
| 72. | O'Sullivan et al., 2020 [72] | No relevant/useable outcome data |
| 73. | O'Sullivan et al., 2009 [73] | Not a RAM for predicting the risk of developing VTE in pregnancy or the puerperium |
| 74. | Ottawa Hospital Research Institute and Leo Pharma 2002 [74] | Protocol |
| 75. | Palmerola et al., 2016 [75] | No relevant/useable outcome data |
| 76. | Pannucci and Fleming 2017 [76] | Not a RAM for predicting the risk of developing VTE in pregnancy or the puerperium |
| 77. | Pierce-Williams et al., 2018 [77] | No relevant/useable outcome data |
| 78. | Potdar et al., 2006 [78] | No relevant/useable outcome data |
| 79. | Rahim et al., 2020 [79] | Not a RAM for predicting the risk of developing VTE in pregnancy or the puerperium |
| 80. | Righini et al., 2013 [80] | Not a RAM for predicting the risk of developing VTE in pregnancy or the puerperium |
| 81. | Righini and Le Gal 2013 [81] | Not a RAM for predicting the risk of developing VTE in pregnancy or the puerperium |
| 82. | Ryan 2019 [82] | No relevant/useable outcome data |
| 83. | Saad et al., 2018 [83] | Abstract of included full text study (Tran 2019) |
| 84. | Santos et al., 2015 [84] | No relevant/useable outcome data |
| 85. | Schoenbeck et al., 2011 [85] | No relevant/useable outcome data |
| 86. | Sellappan et al., 2012 [86] | Not a RAM for predicting the risk of developing VTE in pregnancy or the puerperium |
| 87. | Shacaluga et al., 2017 [87] | No relevant/useable outcome data |

For peer review only

| | | |
|---|---|---|
| 88. | Tan and Wisdom 2006 [88] | No relevant/useable outcome data |
| 89. | Tang and Marsden 2011 [89] | No relevant/useable outcome data |
| 90. | Taylor et al., 2000 [90] | No relevant/useable outcome data |
| 91. | Testa et al., 2010 [91] | No relevant/useable outcome data |
| 92. | Testa et al., 2013 [92] | No relevant/useable outcome data |
| 93. | Touhami et al., 2018 [93] | Not a RAM for predicting the risk of developing VTE in pregnancy or the puerperium |
| 94. | Usoro et al., 2019 [94] | Not a RAM for predicting the risk of developing VTE in pregnancy or the puerperium |
| 95. | Valdre et al., 2016 [95] | No relevant/useable outcome data |
| 96. | Von Hawrylak 2018 [96] | No relevant/useable outcome data |
| 97. | Zhang et al., 2020 [97] | No relevant/useable outcome data |

For peer review only

**REFERENCES (APPENDIX S2)**

1. Abdul Sultan A, West J, Tata LJ, et al. Risk of first venous thromboembolism in pregnant women in hospital: population based cohort study from England. *BMJ (Clinical Research Ed)* 2013;347:f6099. doi: https://dx.doi.org/10.1136/bmj.f6099

2. Ahmadzia HK, Ellis-Kahana JK, Sparks AD, et al. 859: Predicting venous thromboembolism in obese pregnant women in a national study. *American Journal of Obstetrics and Gynecology* 2019;220(1 Supplement):S559-S60. doi: http://dx.doi.org/10.1016/j.ajog.2018.11.882

3. Alsayegh F, Al-Jassar W, Wani S, et al. Venous Thromboembolism Risk and Adequacy of Prophylaxis in High Risk Pregnancy in the Arabian Gulf. *Current Vascular Pharmacology* 2016;14(4):368-73.

4. Bahl V, Hu HM, Henke PK, et al. A validation study of a retrospective venous thromboembolism risk scoring method. *Annals of Surgery* 2010;251(2):344-50. doi: https://dx.doi.org/10.1097/SLA.0b013e3181b7fca6

5. Banfield DA, Page LM, Cotzias CS, et al. Postnatal risk assessment of venous thromboembolism (VTE). *Archives of Disease in Childhood: Fetal and Neonatal Edition* 2013;98(SUPPL. 1) doi: http://dx.doi.org/10.1136/archdischild-2013-303966.121

6. Bare LA, De Haan HG, Arellano AR, et al. A simple genetic thrombosis score of five single nucleotide polymorphisms is associated with risk of first venous thrombosis in pregnant women. *Blood* 2013;122(21)

7. Barros V, Hase E, Igai AM, et al. Cancer and venous thromboembolism in pregnancy. *Research and Practice in Thrombosis and Haemostasis* 2017;1(Supplement 1):54. doi: http://dx.doi.org/10.1002/rth2.12012

8. Barros V, Igai AM, Bortolotto MR, et al. Obesity and thromboembolic risk during hospitalization in pregnancy: Preliminary results. *Research and Practice in Thrombosis and Haemostasis* 2017;1(Supplement 1):1030.

9. Barros V, Igai A, Fernanda B, et al. Preventing maternal death and morbidity from venous thromboembolism (VTE): Results from a VTE risk score trial during hospitalization. *Research and Practice in Thrombosis and Haemostasis* 2020;4(SUPPL 1):1197-98. doi: http://dx.doi.org/10.1002/rth2.12393

10. Barros V, Oliveira A, Spadotto F, et al. Thrombosis risk score in pregnancy: Preliminary results from a model. *Journal of Perinatal Medicine* 2011;39(SUPPL. 1) doi: http://dx.doi.org/10.1515/jpm-2012-1008

11. Bastek JA, Dainty E, Srinivas SK, et al. Pulmonary embolism in pregnancy: Do the diagnostic algorithms from non-pregnant populations apply? *American Journal of Obstetrics and Gynecology* 2011;204(1 SUPPL.):S317. doi: http://dx.doi.org/10.1016/j.ajog.2010.10.834

12. Beckett V, Graham I, Keriakos R. Audit of prescribing and administration of thromboprophylaxis medication and stockings to in-patients of the obstetric and gynaecological wards at the

For peer review only

Jessops Wing, Sheffield teaching hospitals. *BJOG: An International Journal of Obstetrics and Gynaecology* 2013;120(SUPPL. 1):437-38. doi: http://dx.doi.org/10.1111/1471-0528.12301

13. Berkin JA, Lee C, Landsberger E, et al. Scorecard implementation improves identification of postpartum patients at risk for venous thromboembolism. *Journal of Healthcare Risk Management: The Journal of the American Society for Healthcare Risk Management* 2016;36(1):8-13. doi: https://dx.doi.org/10.1002/jhrm.21229
14. Blondon M, Harrington L, Boehlen F, et al. Pre-pregnancy BMI and delivery BMI as risk factors for postpartum VTE: A population-based, case-control study. *Journal of Thrombosis and Haemostasis* 2015;13(SUPPL. 2):106. doi: http://dx.doi.org/10.1111/jth.12993
15. Blondon M, Hugon-Rodin J. A clinical risk score to predict the incidence of postpartum venous thromboembolism. *Evidence-Based Medicine* 2017;22(3):98. doi: http://dx.doi.org/10.1136/ebmed-2017-110680
16. Campbell MJ. Full audit cycle assessing how current antenatal inpatients are risk assessed for venous thromboembolic (VTE) disease as an inpatient and antenatally at Ninewells Hospital, Dundee, Scotland, October 2012. *BJOG: An International Journal of Obstetrics and Gynaecology* 2013;120(SUPPL. 1):472. doi: http://dx.doi.org/10.1111/1471-0528.12301
17. Cavazza S, Rainaldi MP, Bonazzi F, et al. Thomboprophylaxis following cesarean section: A one site prospective pilot study to evaluate the application of a risk score model. *Pathophysiology of Haemostasis and Thrombosis* 2010;37(SUPPL. 1):A4. doi: http://dx.doi.org/10.1159/000318095
18. Chauleur C, Gris JC, Laporte S, et al. Implementation of risk score-guided prophylaxis in over 2000 pregnant women at risk of thrombotic events: Impact on morbidity. A quasi-experimental prospective study. *Thrombosis Research* 2017;151(Supplement 1):S107-S08.
19. Chauleur C, Gris JC, Laporte S, et al. Use of the Delphi method to facilitate antithrombotics prescription during pregnancy. *Thrombosis Research* 2010;126(2):88-92. doi: https://dx.doi.org/10.1016/j.thromres.2010.01.012
20. Chauleur C, Gris J-C, Laporte S, et al. Benefit of Risk Score-Guided Prophylaxis in Pregnant Women at Risk of Thrombotic Events: A Controlled Before-and-After Implementation Study. *Thrombosis and Haemostasis* 2018;118(9):1564-71. doi: https://dx.doi.org/10.1055/s-0038-1668524
21. Cooley SM, Donnelly JC, Deering M, et al. Thrombocalc: Personalized postpartum VTE risk assessment in a high-throughput environment. *American Journal of Obstetrics and Gynecology* 2016;214(1 SUPPL. 1):S26-S27.
22. Creagh MD, Davies B, Webborn A, et al. Practice using a programme for avoidance of venous thrombo-embolism (VTE) in pregnancy; Is post delivery risk assessment undertaken and

For peer review only

effective? *British Journal of Haematology* 2014;165(SUPPL. 1):44-45. doi: http://dx.doi.org/10.1111/bjh.12802

23. Creagh MD, Dehnel A, Rider L, et al. Does systematic risk assessment in pregnancy identify women at risk for venous thromboembolism and so avoid thrombosis? Experience of an 18 month programme based on national guidance. *Journal of Thrombosis and Haemostasis* 2013;11(SUPPL. 2):867.
24. Crowley MP, Noone C, Higgins JR, et al. Venous thromboembolism (VTE) prophylaxis in hospitalized obstetric patients in Ireland: A multicentre cross-sectional study. *Journal of Thrombosis and Haemostasis* 2013;11(SUPPL. 2):739.
25. Crowley MP, Noone C, Higgins JR, et al. A Multicentre Study of Thromboprophylaxis in Pregnancy. *Irish Medical Journal* 2017;110(5):567.
26. Crowley MP, Noone C, Higgins JR, et al. Venous thromboembolism (VTE) prophylaxis in hospitalized obstetric patients: A multicentre cross-sectional study. *Haematologica* 2013;98(SUPPL. 1):438.
27. Cunningham Y, MacDonald H, Coutts S. Bettering antenatal and postnatal risk assessment for venous thromboembolism - A quality improvement approach. *BJOG: An International Journal of Obstetrics and Gynaecology* 2015;122(SUPPL. 2):263. doi: http://dx.doi.org/10.1111/14710528.13383
28. Cutts BA, Tran HA, Merriman E, et al. The utility of the Wells clinical prediction model and ventilation-perfusion scanning for pulmonary embolism diagnosis in pregnancy. *Blood Coagulation & Fibrinolysis: An International Journal in Haemostasis and Thrombosis* 2014;25(4):375-8. doi: https://dx.doi.org/10.1097/MBC.0000000000000054
29. Cutts BA, Tran H, Merriman E, et al. The utility of the wells clinical prediction model and ventilation-perfusion scanning for pulmonary embolism in pregnancy. *Journal of Thrombosis and Haemostasis* 2011;9(SUPPL. 2):631. doi: http://dx.doi.org/10.1111/j.1538-7836.2011.04380_3.x
30. Dargaud YG, Rugeri L, Fleury C, et al. Individually tailored prophylaxis using a risk score for the management of pregnant women with increased risk of venous thromboembolism. *Blood* 2015;126(23):889.
31. Dargaud Y, Rugeri L, Vergnes MC, et al. A risk score for the management of pregnant women with increased risk of venous thromboembolism: a multicentre prospective study. *British Journal of Haematology* 2009;145(6):825-35. doi: https://dx.doi.org/10.1111/j.1365-2141.2009.07698.x
32. Dargaud Y, Rugeri L, Vergnes MC, et al. Management of pregnancies with high risk of thrombosis: A multicenter prospective study. *Journal of Thrombosis and Haemostasis* 2009;7(S2):1060-61. doi: http://dx.doi.org/10.1111/j.1538-7836.2009.03473-2.x

For peer review only

33. Davis B, Hadpawat-Lee A. Venous thromboembolism: Comparing risk assessment tools for postpartum prophylaxis in a teaching hospital. *Obstetrics and Gynecology* 2017;129(Supplement 1):114S.

34. Dentali F, Fontanella A, Cohen AT, et al. Derivation and Validation of a Prediction Model for Venous Thromboembolism in Primary Care. *Thrombosis and Haemostasis* 2020;120(4):692-701. doi: http://dx.doi.org/10.1055/s-0040-1701483

35. Francis Kim AP, Saleh M, Melendez Torres A, et al. Age and body mass index can screen for VTE risk at labor and delivery admission. *Obstetrics and Gynecology* 2020;135(Supplement 1):39S.

36. Francis Kim AP, Saleh M, Torres AM, et al. Impact of delivery-related factors on venous thromboembolism risk during labor. *Obstetrics and Gynecology* 2020;135(Supplement 1):128S.

37. Fuller GW, Nelson-Piercy C, Hunt BJ, et al. Consensus-derived clinical decision rules to guide advanced imaging decisions for pulmonary embolism in pregnancy and the postpartum period. *European Journal of Emergency Medicine: Official Journal of the European Society for Emergency Medicine* 2018;25(3):221-22. doi: https://dx.doi.org/10.1097/MEJ.0000000000000477

38. Gassmann N, Viviano M, Fontana P, et al. Comparison of recommended postpartum thromboprophylaxis and of absolute risk thresholds according to the RCOG, ACCP and ACOG guidelines. *Research and Practice in Thrombosis and Haemostasis* 2020;4(SUPPL 1):32-33. doi: http://dx.doi.org/10.1002/rth2.12401

39. Gerhardt A, Scharf RE, Greer IA, et al. Hereditary risk factors for thrombophilia and probability of venous thromboembolism during pregnancy and the puerperium. *Blood* 2016;128(19):2343-49. doi: http://dx.doi.org/10.1182/blood-2016-03-703728

40. Gherghe M, Moss H, Gibson J, et al. Risk assessment to reduce thromboembolic disease in obstetric practice: A regional audit. *Archives of Disease in Childhood: Fetal and Neonatal Edition* 2012;97(SUPPL. 1):A49. doi: http://dx.doi.org/10.1136/fetalneonatal-2012-301809.156

41. Goffman D, Fisher N, Kowenski J, et al. Utilization of a checklist to evaluate risk for postpartum venous thromboembolism. *American Journal of Obstetrics and Gynecology* 2009;201(6 SUPPL. 1):S297. doi: http://dx.doi.org/10.1016/j.ajog.2009.10.848

42. Gomez D, Orfanelli T, Awomolo A, et al. A comparison of pregnancy-specific risk scoring systems for venous thromboembolic pharmacoprophylaxis in hospitalized maternity patients. *The Journal of Maternal-Fetal & Neonatal Medicine: The Official Journal of the European Association of Perinatal Medicine, the Federation of Asia and Oceania Perinatal Societies, the International Society of Perinatal Obstetricians* 2020:1-8. doi: https://dx.doi.org/10.1080/14767058.2020.1832072

For peer review only

43. Grille S, Castro V, Turcatti P, et al. Prophylaxis for venous thromboembolic disease in pregnancy and postpartum period. *Haematologica* 2015;100(SUPPL. 1):626.

44. Goodfellow A, Heath S, George L, et al. Perinatal venous thromboembolism state based prevention strategy: Midwifery risk assessment tool. *Thrombosis Research* 2017;151(Supplement 1):S132.

45. Grant GH, Merriman JB, Hoffman MK. Implementation and efficacy of a formalized venous thromboembolism prevention strategy in the peripartum population. *American Journal of Obstetrics and Gynecology* 2016;214(1 SUPPL. 1):S227-S28.

46. Handa S, Singh S, Bhandal N. Retrospective audit of venous thromboembolism risk assessment in obstetric postoperative patients. *Anaesthesia* 2015;70(SUPPL. 3):72. doi: http://dx.doi.org/10.1111/anae.13136

47. Harris C, Sulmers C, Groesch K, et al. Venous thromboembolism: Padua prediction score in the obstetric patient. *Obstetrics and Gynecology* 2016;127(Supplement 1):88S. doi: http://dx.doi.org/10.1097/01.AOG.0000483795.00678.28

48. Hayes-Ryan D, Byrne B. Thromboprophylaxis in pregnancy the practical implications of guidelines. *American Journal of Obstetrics and Gynecology* 2011;204(1 SUPPL.):S315-S16. doi: http://dx.doi.org/10.1016/j.ajog.2010.10.829

49. Hayes-Ryan D, Byrne BM. Prevention of thrombosis in pregnancy: how practical are consensus derived clinical practice guidelines? *Journal of Obstetrics and Gynaecology: The Journal of the Institute of Obstetrics and Gynaecology* 2012;32(8):740-2. doi: https://dx.doi.org/10.3109/01443615.2012.693982

50. Heath S, Goodfellow A. Maternal venous thromboembolism (VTE) risk assessment. *Journal of Paediatrics and Child Health* 2016;52(Supplement 2):69. doi: http://dx.doi.org/10.1111/jpc.13194

51. Henke PK, Pannucci CJ. Venous thromboembolism risk factor assessment and prophylaxis. *Phlebology* 2010;25(5):219-23. doi: http://dx.doi.org/10.1258/phleb.2010.010018

52. Kazi S, McLeod A, Berndl A. Approach to venous thromboembolism risk in women with physical disability in pregnancy-multidisciplinary survey. *Research and Practice in Thrombosis and Haemostasis* 2020;4(SUPPL 1):1283-84. doi: http://dx.doi.org/10.1002/rth2.12393

53. LaCoss E, Jheeta S. How accurate is information documented on inpatient electronic venous thromboembolism risk-assessment forms? *Journal of Pharmacy and Pharmacology* 2017;69(Supplement 1):13-14. doi: http://dx.doi.org/10.1111/jphp.12850

54. Li C, Zuo Y, Karp D, et al. Identifying clinical and epidemiological risk factors associated with thrombosis and pregnancy morbidity in a large cohort of Chinese APS patients. *International Journal of Rheumatic Diseases* 2018;21(Supplement 1):126-27. doi: http://dx.doi.org/10.1111/1756-185X.13361

For peer review only

55. Lindqvist PG. Re: Postpartum venous thromboembolism prophylaxis may cause more harm than benefit: a critical analysis of international guidelines through an evidence-based lens: Postpartum thromboprophylaxis is cost-effective using the Swedish thromboprophylaxis algorithm. *BJOG: An International Journal of Obstetrics and Gynaecology* 2018;125(9):1194-95. doi: http://dx.doi.org/10.1111/1471-0528.15266
56. Lindqvist PG, Hellgren M. Obstetric thromboprophylaxis: the Swedish guidelines. *Advances in hematology* 2011;2011:157483. doi: https://dx.doi.org/10.1155/2011/157483
57. Lindqvist PG, Kublikas M, Dahlback B. Individual risk assessment of thrombosis in pregnancy. *Acta Obstetricia et Gynecologica Scandinavica* 2002;81(5):412-6.
58. Lou Mercade AC, Saviron Cornudella R, Cornudella Lacasa R. AnticoagObs: An application for antenatal and postpartum risk assessment and prophylaxis of venous thromboembolism. *Thrombosis Research* 2017;151(Supplement 1):S127.
59. Marks D, Maiti S. Venous thromboembolism risk assessment and prescribing on the postnatal ward at north manchester general hospital: A clinical audit. *BJOG: An International Journal of Obstetrics and Gynaecology* 2018;125(Supplement 3):25. doi: http://dx.doi.org/10.1111/1471-0528.15493
60. McArthur L, Whittaker B, Rajasri A, et al. Venous thrombo-embolic risk in pregnancy and the puerperium: Can self-assessment identify risk and guide further care? *British Journal of Haematology* 2011;153(SUPPL. 1):84-85. doi: http://dx.doi.org/10.1111/j.13652141.2011.08609
61. Mpouzouki A, Bowles L, Beski S, et al. A retrospective review of risk factors for thrombosis in women presenting with venous thromboembolism (VTE) during pregnancy and the puerperium. *Thrombosis Research* 2013;131(SUPPL. 1):S82. doi: http://dx.doi.org/10.1016/S0049-3848%2813%2970070-7
62. Naidoo P, Mothilal R, Snyman LC. Assessment and management of venous thrombo-embolism risk during pregnancy and the puerperium (SAVE): The South African cohort. *South African Medical Journal* 2019;109(3):186-92. doi: https://dx.doi.org/10.7196/SAMJ.2019.v109i3.13487
63. NCT. Pregnancy and Risk of Venous Thromboembolism. *https://clinicaltrialsgov/show/NCT03659708* 2018
64. Noone CM, O'Shea S, Crowley MP, et al. Venous thromboembolism (VTE) risk assessment & prophylaxis in irish hospitalized obstetric patients: A nationwide cross-sectional study. *Blood* 2013;122(21)
65. O'Connor C, Moriarty J, Walsh J, et al. The application of a clinical risk stratification score may reduce unnecessary investigations for pulmonary embolism in pregnancy. *The Journal of Maternal-Fetal & Neonatal Medicine: The Official Journal of the European Association of Perinatal Medicine, the Federation of Asia and Oceania Perinatal Societies, the International*

For peer review only

*Society of Perinatal Obstetricians* 2011;24(12):1461-4. doi: https://dx.doi.org/10.3109/14767058.2011.614652

66. O'Keefe D, Hui L, Ho P, et al. Evaluation of global coagulation assays for assessment of clotting function and risk of venous thromboembolism in pregnancy and post-partum. *Australian and New Zealand Journal of Obstetrics and Gynaecology* 2019;59(Supplement 1):68-69. doi: http://dx.doi.org/10.1111/ajo.13067
67. Omunakwe HE, Roberts LN, Patel JP, et al. Re: A comparison of the recommendations for pharmacologic thromboembolism prophylaxis after caesarean delivery from the major guidelines: Impact on thromboprophylaxis rates of implementing Royal College of Obstetricians and Gynaecologists' guidance for reducing the risk of ante- and postnatal venous thromboembolism. *BJOG: An International Journal of Obstetrics and Gynaecology* 2017;124(5):831-32. doi: http://dx.doi.org/10.1111/1471-0528.14001
68. Orfanelli T, Awomolo A, Gomez D, et al. A comparison of peripartum scoring systems of anticoagulation for venous thromboembolic prophylaxis. *Obstetrics and Gynecology* 2017;129(Supplement 1):112S.
69. O'Shaughnessy F, Donnelly JC, Cooley SM, et al. Thrombocalc: implementation and uptake of personalized postpartum venous thromboembolism risk assessment in a high-throughput obstetric environment. *Acta obstetricia et gynecologica Scandinavica* 2017;96(11):1382-90. doi: https://dx.doi.org/10.1111/aogs.13206
70. O'Shaughnessy F, Donnelly J, Cooley S, et al. Initiating postpartum pharmacological thromboprophylaxis: A comparison of international recommendations. *American Journal of Obstetrics and Gynecology* 2018;218(1 Supplement 1):S493-S94.
71. O'Shaughnessy F, Ni Ainle F, Jennifer D, et al. Preventing postpartum venous thromboembolism; impact of systematic VTE risk assessment. *Research and Practice in Thrombosis and Haemostasis* 2019;3(Supplement 1):225-26. doi: http://dx.doi.org/10.1002/rth2.12227
72. O'Sullivan C, Christensen K, Gallagher G, et al. Peripartum vte risk assessment in a tertiary centre: An audit. *Journal of Paediatrics and Child Health* 2020;56(SUPPL 1):143. doi: http://dx.doi.org/10.1111/jpc.14868
73. O'Sullivan C, Moriarty J, Walsh J, et al. Application of a clinical risk stratification score in pregnancy and the puerperium - Can unnecessary investigations for pulmonary embolism be avoided? *American Journal of Obstetrics and Gynecology* 2009;201(6 SUPPL. 1):S68. doi: http://dx.doi.org/10.1016/j.ajog.2009.10.161
74. Ottawa Hospital Research Institute, LEO Pharma. The STOP CLOT Pilot Study: Study of Low Molecular Weight Heparin in High Risk Cesarean Section: https://ClinicalTrials.gov/show/NCT00225108, 2002.
75. Palmerola KL, D'Alton ME, Brock CO, et al. A comparison of recommendations for pharmacologic thromboembolism prophylaxis after caesarean delivery from three major

For peer review only

guidelines. *BJOG: An International Journal of Obstetrics and Gynaecology* 2016;123(13):2157-62. doi: https://dx.doi.org/10.1111/1471-0528.13706

76. Pannucci C, Fleming K. The electronic medical record underestimates venous thromboembolism risk using the 2005 caprini score compared with face-to-face interaction. *Journal of Vascular Surgery: Venous and Lymphatic Disorders* 2017;5(1):152-53. doi: http://dx.doi.org/10.1016/j.jvsv.2016.10.028

77. Pierce-Williams R, Cohen I, D'Adamo C, et al. Venous thromboembolism prophylaxis after cesarean section: A quality improvement project. *Obstetrics and Gynecology* 2018;131(Supplement 1):104S-05S.

78. Potdar N, Jabbar B, Burrell SJ. Thromboprophylaxis after vaginal delivery: a district general hospital experience. *Journal of Obstetrics and Gynaecology: The Journal of the Institute of Obstetrics and Gynaecology* 2006;26(1):24-6.

79. Rahim MN, Williamson C, Kametas NA, et al. Using pregnancy to assess risk and predict women's health. *EClinicalMedicine* 2020;20:100292. doi: http://dx.doi.org/10.1016/j.eclinm.2020.100292

80. Righini M, Jobic C, Boehlen F, et al. Predicting deep venous thrombosis in pregnancy: external validation of the LEFT clinical prediction rule. *Haematologica* 2013;98(4):545-8. doi: https://dx.doi.org/10.3324/haematol.2012.072009

81. Righini M, Le Gal G. Predicting deep venous thrombosis in pregnancy: External validation of the 'left' clinical prediction rule. *Journal of Thrombosis and Haemostasis* 2013;11(SUPPL. 3):91. doi: http://dx.doi.org/10.1111/jth.12443

82. Ryan N. Maternal thromboprophylaxis assessment tool: An audit. *Irish Journal of Medical Science* 2019;188(Supplement 7):S53. doi: http://dx.doi.org/10.1007/s11845-019-02053-0

83. Saad A, Tran JP, Stribling S, et al. Performance of risk assessment models for thromboprophylaxis in an obstetric population. *American Journal of Obstetrics and Gynecology* 2018;218(1 Supplement 1):S48-S49.

84. Santos R, Barros VV, Igai AK, et al. Maternal death and Venous Thromboembolism (VTE) in patients admitted in a maternity of high risk: Results pre and post application of a risk score. *Journal of Thrombosis and Haemostasis* 2015;13(SUPPL. 2):679. doi: http://dx.doi.org/10.1111/jth.12993

85. Schoenbeck D, Nicolle A, Newbegin K, et al. The use of a scoring system to guide thromboprophylaxis in a high-risk pregnant population. *Thrombosis* 2011;2011:652796. doi: https://dx.doi.org/10.1155/2011/652796

86. Sellappan KV, Farr R, Hill P. An audit on risk assessment of women at antenatal booking appointment. *International Journal of Gynecology and Obstetrics* 2012;119(SUPPL. 3):S703-S04. doi: http://dx.doi.org/10.1016/S0020-7292%2812%2961724-X

For peer review only

87. Shacaluga A, Wallace P, Rayment R. Abandoning RCOG Guideline 37a: A risk worth taking? *BJOG: An International Journal of Obstetrics and Gynaecology* 2017;124(Supplement 1):142. doi: http://dx.doi.org/10.1111/1471-0528.9_14572
88. Tan EK, Wisdom SJ. Thromboprophylaxis post vaginal delivery: are we forgetting it? Audit on thromboprophylaxis prescription post vaginal births. *Journal of Obstetrics and Gynaecology: The Journal of the Institute of Obstetrics and Gynaecology* 2006;26(1):27-9.
89. Tang Z, Marsden PJ. Risk assessment and management of venous thrombo-embolism in obstetric women. *Archives of Disease in Childhood: Fetal and Neonatal Edition* 2011;96(SUPPL. 1) doi: http://dx.doi.org/10.1136/adc.2011.300163.65
90. Taylor GM, McKenzie CA, Mires GJ. Use of a computerised maternity information system to improve clinical effectiveness: thromboprophylaxis at caesarean section. *Postgraduate Medical Journal* 2000;76(896):354-6.
91. Testa S, Paoletti O, Ronca E, et al. Health care program for thromboembolism prevention in pregnancy. *Pathophysiology of Haemostasis and Thrombosis* 2010;37(SUPPL. 1):A76. doi: http://dx.doi.org/10.1159/000318097
92. Testa S, Passamonti SM, Paoletti O, et al. The pregnancy health-care program: A model for the prevention of venous thromboembolism in pregnancy. *Journal of Thrombosis and Haemostasis* 2013;11(SUPPL. 2):606.
93. Touhami O, Marzouk SB, Bennasr L, et al. Are the Wells Score and the Revised Geneva Score valuable for the diagnosis of pulmonary embolism in pregnancy? *European Journal of Obstetrics, Gynecology, and Reproductive Biology* 2018;221:166-71. doi: https://dx.doi.org/10.1016/j.ejogrb.2017.12.049
94. Usoro E, Sakurai H, Aedla N. Investigation and management of venous thromboembolism in pregnancy. *European Journal of Obstetrics Gynecology and Reproductive Biology* 2019;234:e105. doi: http://dx.doi.org/10.1016/j.ejogrb.2018.08.390
95. Valdre L, Lambertini I, Palareti G, et al. Comparison of strategies for preventing venous thromboembolism in high risk pregnant women according to national and international guidelines: Results of a prospective cohort study. *Blood Transfusion* 2016;14(Supplement 5):s747.
96. Von Hawrylak F. Implementing a venous thromboembolism risk assessment in an abortion service. *European Journal of Contraception and Reproductive Health Care* 2018;23(Supplement 1):43. doi: http://dx.doi.org/10.1080/13625187.2018.1442911
97. Zhang W, Shen J, Sun J-L. Risk scores, prevention, and treatment of maternal venous thromboembolism. *World Journal of Clinical Cases* 2020;8(11):2210-18. doi: https://dx.doi.org/10.12998/wjcc.v8.i11.2210

For peer review only

**APPENDIX S3 SIMPLIFIED SUMMARY OF WIDELY EVALUATED GENERIC RAMS, THEIR ASSOCIATED CHARACTERISTICS AND COMPOSITE CLINICAL VARIABLES**

| Characteristics | Name of VTE risk assessment model | | | |
|---|---|---|---|---|
| | RCOG | ACOG | SFOG | Lyon score |
| **General** | | | | |
| Author, year | Royal College of Obstetricians and Gynaecologists, 2015[1] | James et al., 2018[2] | Lindquist et al., 2008[3] and Lindqvist & Hellgren, 2011[4] | Dargaud et al., 2017[5] |
| Applicable cohort | All pregnant and postpartum women | All pregnant and postpartum women at risk | Pregnant women with moderate-high risk of VTE | Pregnant women with high risk of thrombosis |
| Design | Risk factor based with cumulative score | Risk factor based | Risk factor based with cumulative score | Risk factor based with cumulative score |
| Number of VTE risk variables | 26 | Not specified | 23 | 15 |
| When is pharmacological thromboprophylaxis recommended? | • Score ≥ 4 antenatally (from first trimester)<br>• Score 3 antenatally (from 28 weeks)<br>• Score ≥ 2 postnatally (at least 10 days).<br>• Antenatal hospital admission<br>• Prolonged hospital admission (≥ 3 days) or readmission to hospital within puerperium | • All women with acute VTE during pregnancy, or women with history of thrombosis or those at significant risk of VTE during pregnancy or the postpartum period such as those with thrombophilia | • Very high risk (high dose antepartum and at least 12 weeks postpartum)[a]<br>• Score ≥ 4 (antepartum and 6 weeks postpartum)<br>• Score 3 (after delivery [6 weeks])<br>• Score 2 (after delivery [7 days] or during immobilisation) | • Score ≥ 6 antenatally or postnatally<br>• Score between 3 and 5, from third trimester |
| **Pre-existing risk factors** | | | | |

For peer review only

| | | | | |
|---|---|---|---|---|
| Previous VTE (personal) | Yes (except a single event related to major surgery) | Yes | Yes | Yes (pregnancy related, CVT or massive PE or VTE in childhood [<16 years]; unprovoked or oestrogen related; transient risk factor induced) |
| Recurrent VTE | No | Yes | Yes | Yes (personal history; residual venous thrombi with clinical signs of PTS, recent <2 years) |
| Previous VTE provoked by specific event | Yes (major surgery) | Yes (surgery, trauma or immobility AND additional major thrombotic risk factors)[b] | No | No |
| Family history of VTE | Yes (unprovoked or estrogen related) | Yes (first degree with thrombophilia) | Yes (first degree <60 years) | Yes (severe or recurrent) |
| Thrombophilia e.g. Factor V Leiden and Factor II mutations; protein C, protein S and antithrombin deficiency; antiphospholipid syndrome (with or without VTE) | Yes (various forms | Yes (various forms) | Yes (various forms) | Yes (various forms) |
| Medical comorbidities | Yes (3 points for any individual comorbidities) | No | Yes (inflammatory bowel disease) | No |
| Age | Yes (>35 years) | No | Yes (>40 years) | Yes (>35 years) |
| Obesity | Yes (≥30kg/m$^2$; ≥40kg/m$^2$) | No | Yes (>28kg/m$^2$ in early pregnancy) | Yes (≥30kg/m$^2$) |

| | | | | |
|---|---|---|---|---|
| Parity | Yes (≥3) | No | No | No |
| Smoker | Yes | No | No | No |
| Varicose veins | Yes (gross) | No | No | No |
| Hyperhomocysteinemia | No | No | Yes (homocysteine >8 $\mu$mol/L in pregnancy | No |
| Mechanical heart prosthesis | No | No | Yes | No |
| Chronic warfarin prophylaxis | No | No | Yes | No |
| **Obstetric** | | | | |
| Pre-eclampsia | Yes (current pregnancy) | No | Yes | No |
| ART/IVF | Yes (antenatal only) | No | No | No |
| Multiple pregnancy | Yes | No | No | Yes |
| Caesarean section | Yes (elective/in labour) | No | Yes | No |
| Mid-cavity or rotational operative delivery | Yes | No | No | No |
| Prolonged labour (> 24 hours) | Yes | No | No | No |
| Postpartum haemorrhage | Yes (>1 litre or transfusion) | No | No | No |
| Preterm birth | Yes (<37 weeks, current pregnancy) | No | No | No |
| Stillbirth | Yes (current pregnancy) | No | No | No |
| Abruptio placenta | No | No | Yes | No |
| **Transient factors** | | | | |
| Any surgical procedure | Yes (pregnancy or puerperium except immediate repair of the perineum) | No | No | No |
| Hyperemesis | Yes | No | No | No |

For peer review only

| | | | | |
|---|---|---|---|---|
| Ovarian hyperstimulation syndrome | Yes (first trimester only) | No | No | No |
| Systemic infection | Yes (current) | No | No | No |
| Immobility | Yes (current and dehydration) | No | Yes | Yes |
| **Other** | | | | |
| 'Other risk factors' | No | No | Yes (according to clinical decision) | No |

[a] Thromboprophylaxis initiated as early as possible (sometimes before pregnancy). Only women with antithrombin deficiency, chronic warfarin prophylaxis, recurrent VTE, antiphospholipid syndrome with VTE, and those with mechanical heart prosthesis are included in this group

[b] First-degree relative with a history of a thrombotic episode, or other major thrombotic risk factors (e.g., obesity, prolonged immobility, caesarean delivery

ACOG, American College of Obstetricians and Gynaecologists; ART/IVF Assisted reproductive technology/ In vitro fertilization; RCOG, Royal College of Obstetricians and Gynaecologists; SFOG, Swedish Society of Obstetrics and Gynecology; VTE, venous thromboembolism

## REFERENCES (APPENDIX S3)


1. Royal College of Obstetricians & Gynaecologists. Thrombosis and Embolism during Pregnancy and the Puerperium, Reducing the Risk (Green-top Guideline No. 37a) 2015 [Available from: https://www.rcog.org.uk/en/guidelines-research-services/guidelines/gtg37a/ accessed January 2022.
2. James A, Birsner M, Kaimal A, et al. ACOG Practice Bulletin No. 196. Summary: thromboembolism in pregnancy. *Obstet Gynecol* 2018;132:243-8.
3. Lindqvist PG, Torsson J, Almqvist A, et al. Postpartum thromboembolism: severe events might be preventable using a new risk score model. *Vascular Health and Risk Management* 2008;4(5):1081-7.
4. Lindqvist PG, Hellgren M. Obstetric thromboprophylaxis: the Swedish guidelines. *Advances in Hematology* 2011;2011:157483. doi: https://dx.doi.org/10.1155/2011/157483
5. Dargaud Y, Rugeri L, Fleury C, et al. Personalized thromboprophylaxis using a risk score for the management of pregnancies with high risk of thrombosis: a prospective clinical study. *Journal of Thrombosis and Haemostasis* 2017;15(5):897-906.


For peer review only

For peer review only

# PRISMA 2020 Checklist

| Section and Topic | Item # | Checklist item | Location where item is reported |
|---|---|---|---|
| **TITLE** | | | |
| Title | 1 | Identify the report as a systematic review. | Title page (page 1) |
| **ABSTRACT** | | | |
| Abstract | 2 | See the PRISMA 2020 for Abstracts checklist. | Abstract (pages 2-3) |
| **INTRODUCTION** | | | |
| Rationale | 3 | Describe the rationale for the review in the context of existing knowledge. | Introduction (pages 5-6) |
| Objectives | 4 | Provide an explicit statement of the objective(s) or question(s) the review addresses. | Introduction (pages 5-6) |
| **METHODS** | | | |
| Eligibility criteria | 5 | Specify the inclusion and exclusion criteria for the review and how studies were grouped for the syntheses. | Eligibility criteria (page 6) |
| Information sources | 6 | Specify all databases, registers, websites, organisations, reference lists and other sources searched or consulted to identify studies. Specify the date when each source was last searched or consulted. | Data sources and searches (pages 6-7) |
| Search strategy | 7 | Present the full search strategies for all databases, registers and websites, including any filters and limits used. | Appendix S1 |
| Selection process | 8 | Specify the methods used to decide whether a study met the inclusion criteria of the review, including how many reviewers screened each record and each report retrieved, whether they worked independently, and if applicable, details of automation tools used in the process. | Study selection (page 7) |
| Data collection process | 9 | Specify the methods used to collect data from reports, including how many reviewers collected data from each report, whether they worked independently, any processes for obtaining or confirming data from study investigators, and if applicable, details of automation tools used in the process. | Data abstraction and quality assessment (pages 7-8) |
| Data items | 10a | List and define all outcomes for which data were sought. Specify whether all results that were compatible with each outcome domain in each study were sought (e.g. for all measures, time points, analyses), and if not, the methods used to decide which results to collect. | Data abstraction and quality assessment (pages 7-8) |
| | 10b | List and define all other variables for which data were sought (e.g. participant and intervention characteristics, funding sources). Describe any assumptions made about any missing or unclear information. | Data abstraction and quality assessment (pages 7-8) |
| Study risk of bias assessment | 11 | Specify the methods used to assess risk of bias in the included studies, including details of the tool(s) used, how many reviewers assessed each study and whether they worked independently, and if applicable, details of automation tools used in the process. | Data abstraction and quality assessment (pages 7-8) |
| Effect measures | 12 | Specify for each outcome the effect measure(s) (e.g. risk ratio, mean difference) used in the synthesis or presentation of results. | Data synthesis and analysis (pages 8-9) |
| Synthesis methods | 13a | Describe the processes used to decide which studies were eligible for each synthesis (e.g. tabulating the study intervention characteristics and comparing against the planned groups for each synthesis (item #5)). | Data synthesis and analysis (pages 8-9) |
| | 13b | Describe any methods required to prepare the data for presentation or synthesis, such as handling of missing summary statistics, or data conversions. | Data synthesis and analysis (pages 8-9) |

For peer review only

# PRISMA 2020 Checklist

| Section and Topic | Item # | Checklist item | Location where item is reported |
|---|---|---|---|
| | 13c | Describe any methods used to tabulate or visually display results of individual studies and syntheses. | Data synthesis and analysis (pages 8-9) |
| | 13d | Describe any methods used to synthesize results and provide a rationale for the choice(s). If meta-analysis was performed, describe the model(s), method(s) to identify the presence and extent of statistical heterogeneity, and software package(s) used. | Data synthesis and analysis (pages 8-9) |
| | 13e | Describe any methods used to explore possible causes of heterogeneity among study results (e.g. subgroup analysis, meta-regression). | Data synthesis and analysis (pages 8-9) |
| | 13f | Describe any sensitivity analyses conducted to assess robustness of the synthesized results. | Data synthesis and analysis (pages 8-9) |
| Reporting bias assessment | 14 | Describe any methods used to assess risk of bias due to missing results in a synthesis (arising from reporting biases). | Data synthesis and analysis (pages 8-9) |
| Certainty assessment | 15 | Describe any methods used to assess certainty (or confidence) in the body of evidence for an outcome. | N/A |
| **RESULTS** | | | |
| Study selection | 16a | Describe the results of the search and selection process, from the number of records identified in the search to the number of studies included in the review, ideally using a flow diagram. | Study flow (pages 9-10) |
| | 16b | Cite studies that might appear to meet the inclusion criteria, but which were excluded, and explain why they were excluded. | Appendix S2 |
| Study characteristics | 17 | Cite each included study and present its characteristics. | Study and patient characteristics (pages 10-12) |
| Risk of bias in studies | 18 | Present assessments of risk of bias for each included study. | Risk of bias and applicability assessment (pages 13-15) |
| Results of individual studies | 19 | For all outcomes, present, for each study: (a) summary statistics for each group (where appropriate) and (b) an effect estimate and its precision (e.g. confidence/credible interval), ideally using structured tables or plots. | Predictive performance of VTE RAMs (summary of results) (pages 16-19) |
| Results of syntheses | 20a | For each synthesis, briefly summarise the characteristics and risk of bias among contributing studies. | Risk of bias and applicability assessment (pages 13-15) and Predictive performance of VTE RAMs (summary of results) (pages 16-19) |
| | 20b | Present results of all statistical syntheses conducted. If meta-analysis was done, present for each the summary estimate and its precision (e.g. confidence/credible interval) and measures of statistical heterogeneity. If comparing groups, describe the direction of the effect. | Predictive performance of VTE RAMs (summary of results) (pages 16-19) |
| | 20c | Present results of all investigations of possible causes of heterogeneity among study results. | Predictive performance of VTE RAMs (summary of results) (pages 16-19) |
| | 20d | Present results of all sensitivity analyses conducted to assess the robustness of the synthesized results. | N/A |
| Reporting biases | 21 | Present assessments of risk of bias due to missing results (arising from reporting biases) for each synthesis assessed. | Predictive performance of VTE RAMs (summary of results) (pages 16-19) |

For peer review only

For peer review only - http://bmjopen.bmj.com/site/about/guidelines.xhtml

# PRISMA 2020 Checklist

| Section and Topic | Item # | Checklist item | Location where item is reported |
|---|---|---|---|
| Certainty of evidence | 22 | Present assessments of certainty (or confidence) in the body of evidence for each outcome assessed. | N/A |
| **DISCUSSION** | | | |
| Discussion | 23a | Provide a general interpretation of the results in the context of other evidence. | Discussion – Summary of results (page 20); Interpretation of results (pages 20-21); and Comparison to the existing literature (page 21) |
| | 23b | Discuss any limitations of the evidence included in the review. | Discussion – Interpretation of results (pages 20-21); and Comparison to the existing literature (page 21) |
| | 23c | Discuss any limitations of the review processes used. | Discussion – Strengths and limitations (pages 21-23) |
| | 23d | Discuss implications of the results for practice, policy, and future research. | Discussion - Implications for patients, clinicians and policymakers and future research (pages 23-24) |
| **OTHER INFORMATION** | | | |
| Registration and protocol | 24a | Provide registration information for the review, including register name and registration number, or state that the review was not registered. | Page 3 and 6 |
| | 24b | Indicate where the review protocol can be accessed, or state that a protocol was not prepared. | Page 3 and 6 |
| | 24c | Describe and explain any amendments to information provided at registration or in the protocol. | N/A |
| Support | 25 | Describe sources of financial or non-financial support for the review, and the role of the funders or sponsors in the review. | Funding section (page 25) |
| Competing interests | 26 | Declare any competing interests of review authors. | Competing interest section (page 25) |
| Availability of data, code and other materials | 27 | Report which of the following are publicly available and where they can be found: template data collection forms; data extracted from included studies; data used for all analyses; analytic code; any other materials used in the review. | All data relevant to the study are included in the article or uploaded as Appendices (page 25– see data sharing statement) |

*From:* Page MJ, McKenzie JE, Bossuyt PM, Boutron I, Hoffmann TC, Mulrow CD, et al. The PRISMA 2020 statement: an updated guideline for reporting systematic reviews. BMJ 2021;372:n71. doi: 10.1136/bmj.n71

For more information, visit: http://www.prisma-statement.org/

For peer review only
